# Quantifying uncert-AI-nty: Testing the accuracy of LLMs’ confidence judgments

**DOI:** 10.3758/s13421-025-01755-4

**Published:** 2025-07-22

**Authors:** Trent N. Cash, Daniel M. Oppenheimer, Sara Christie, Mira Devgan

**Affiliations:** 1 Department of Social and Decision Sciences, Carnegie Mellon University, 5000 Forbes Ave., 224 Porter Hall, Pittsburgh, PA 15213 USA; 2 Department of Psychology, Carnegie Mellon University, Pittsburgh, PA USA

**Keywords:** Metacognition, Large Language Models, Artificial intelligence, Confidence judgments, Metacognitive accuracy

## Abstract

The rise of Large Language Model (LLM) chatbots, such as ChatGPT and Gemini, has revolutionized how we access information. These LLMs can answer a wide array of questions on nearly any topic. When humans answer questions, especially difficult or uncertain questions, they often accompany their responses with metacognitive confidence judgments indicating their belief in their accuracy. LLMs are certainly capable of providing confidence judgments, but it is currently unclear how accurate these confidence judgments are. To fill this gap in the literature, the present studies investigate the capability of LLMs to quantify uncertainty through confidence judgments. We compare the absolute and relative accuracy of confidence judgments made by four LLMs (ChatGPT, Bard/Gemini, Sonnet, Haiku) and human participants in both domains of aleatory uncertainty—NFL predictions (Study 1; *n* = 502) and Oscar predictions (Study 2; *n* = 109)—and domains of epistemic uncertainty—Pictionary performance (Study 3; *n* = 164), Trivia questions (Study 4; *n* = 110), and questions about life at a university (Study 5; *n* = 110). We find several commonalities between LLMs and humans, such as achieving similar levels of absolute and relative metacognitive accuracy (although LLMs tend to be slightly more accurate on both dimensions). Like humans, we also find that LLMs tend to be overconfident. However, we find that, unlike humans, LLMs—especially ChatGPT and Gemini—often fail to adjust their confidence judgments based on past performance, highlighting a key metacognitive limitation.

**Supplementary Information:**

The online version contains supplementary material available at 10.3758/s13421-025-01755-4.

## Introduction

In everyday interactions, it is common to ask others to evaluate their confidence in the accuracy of the claims they make. These confidence judgments are useful for determining the extent to which listeners should update their mental models to incorporate the speaker’s beliefs. Given the importance of confidence in behaviors and interpersonal communication, it is no surprise that psychologists and other social scientists have long been fascinated by confidence judgments and the accuracy with which they are provided (Einhorn & Hogarth, 1978; Fleming, 2024; Moore & Schatz, 2017).

Despite psychologists’ persistent interest in confidence judgments, the extant psychological literature has focused almost exclusively on confidence judgments made by humans, with some limited research on nonhuman animals, such as monkeys and dolphins (Shields et al., 2005; Smith et al., 1995). Since 2022, however, we have witnessed the emergence and mass popularization of generative artificial intelligence (AI) chatbots that can respond to questions using human-like language. As such, it is reasonable to wonder how the accuracy of AI chatbots’ confidence judgments compares with the accuracy of confidence judgments made by humans.

### Large Language Model chatbots

Researchers have long been interested in developing computer systems that can understand and respond to human language—an ability referred to as natural language processing (NLP; Chowdhary, 2020; Nadkarni et al., 2011). One way to achieve this is to feed the computer system (i.e., the model) a large amount of text so that it can learn relationships between aspects of language—such as which words frequently co-occur and how sentences are typically structured (Chowdhary, 2020; Nadkarni et al., 2011). In the early stages of NLP, these models functioned based on relatively simple handwritten rules, but modern NLP relies on advanced, statistically driven machine learning techniques, such as neural networks and deep learning, that allow models to be trained on vast amounts of data and to develop highly parameterized algorithms that can generate more complex and human-like responses (Hirschberg & Manning, 2015; Nadkarni et al., 2011; Schmidhuber, 2015; Wu et al., 2023). Because these models are trained on so much data, they are often referred to as Large Language Models (LLMs).

Generative AI chatbots—such as ChatGPT, Gemini, Claude, and LLaMA—are built on LLMs. The LLMs underlying these models were trained on vast swaths of the internet and contain billions, and in some cases trillions, of parameters that allow them to develop highly nuanced understandings of human language (OpenAI, 2024; Roose, 2023; Wu et al., 2023). Based on this understanding, the models can accept prompts and use their many parameters to predict what the most likely (or most logical) response to the prompt would be—which usually results in a sensible and relatively accurate output. Ultimately, however, generative AI chatbots are highly sophisticated machines that are extremely good at one task: predicting what text is likely to come next (Roose, 2023; Wolfram, 2023).

### LLM performance on experimental tasks

A great deal of scholarly work has sought to evaluate the capabilities of generative AI chatbots and their underlying LLMs. From a machine learning perspective, this research has largely consisted of benchmarking the performance of various LLMs on certain tasks—such as text comprehension (Bandarkar et al., 2024), mathematical reasoning (Lu et al., 2023), or knowledge about the world (Yu et al., 2023). Researchers have also sought to holistically evaluate LLM performance by benchmarking numerous domains at once (Bommasani et al., 2023; Liang et al., 2022; Spangher et al., 2024). Different models use different training sets and thus have different strengths and weaknesses (Banerjee et al., 2023; N. B. Brown, 2024).

Meanwhile, psychologists and other behavioral scientists have focused on investigating the extent to which generative AI chatbots and their underlying LLMs exhibit human-like cognitive abilities and how their performance in these domains compares to that of humans. For example, Hagendorff et al. (2023) tested various GPT models on cognitive reflection test (CRT) style questions and found that the advanced models (e.g., GPT-3.5, GPT-4) are less likely than humans to give incorrect intuitive responses. Strachan et al. (2024) tested GPT-4, GPT-3.5, and LLaMA2 on a battery of Theory of Mind (ToM) tasks and found that GPT-4 largely outperformed humans, while GPT-3.5 and LLaMA2 had more mixed results (but see Rodriguez & Oppenheimer, 2024, for counterexamples). Elyoseph et al. (2023) tested GPT performance on the Levels of Emotional Awareness Scale and found that GPT demonstrated greater emotional awareness than humans typically do. There is also emerging evidence suggesting that LLMs show many of the same social biases that humans do such as stereotyping based on race (Lee et al., 2024; Omiye et al., 2023) and gender (Wan et al., 2023).

Behavioral scientists have been particularly interested in LLM performance in the domain of judgment and decision-making. For example, Horton (2023) demonstrated that GPT-3 effectively simulates human performance on a variety of economic tasks, including tasks assessing social and fairness preferences. Similarly, Talboy and Fuller (2023) found that ChatGPT-3.5, ChatGPT-4, and Bard were susceptible to many of the same cognitive biases as humans, including representativeness and base rate neglect. Chen et al. (2023) tested GPT-3.5-turbo’s performance in a series of rational decision-making tasks and found that the LLM was better at making utility-maximizing decisions than humans were and that, like humans, their choices were sensitive to framing. Binz and Schulz (2023) found that GPT-3 performed as well or better than humans on a series of numeric reasoning tasks (e.g., the multi-armed bandit task), but that it routinely failed causal reasoning tasks. Relatedly, Zhu and Griffiths (2024) tested the coherence of probability judgments made by multiple LLMs and found that, like humans, their judgments demonstrated significant deviations from probability theory.

These findings represent only a subset of the literature evaluating the cognitive capacities of generative AI chatbots and their underlying LLMs. However, they all point to the conclusion that LLMs—despite the fact that they are simply predictive text models (Roose, 2023; Wolfram, 2023; Wu et al., 2023)—have emergently developed the ability to respond to a number of complex cognitive tasks in a human-like (or better-than-human) way. Whether these results reflect true cognition by the LLMs or just an excellent capacity to mirror human behavior is an open question.

### LLM metacognition

Though there has been great empirical interest in the cognitive capacities of generative AI chatbots and their underlying LLMs, far less attention has been paid to their metacognitive capacities—their ability to think about their own cognition (A. Brown, 1987; Flavell, 1979). The relative lack of attention is likely because it is believed that generative AI chatbots lack introspective self-awareness (Li et al., 2024; Long, 2023). However, the literature on human metacognition suggests that introspection is not a necessary prerequisite of metacognition—but rather that humans can also make metacognitive judgments based on cues they observe while completing the cognitive task (Ackerman & Thompson, 2017; Alter & Oppenheimer, 2009; Nisbett & Wilson, 1977; Pronin & Kugler, 2007; Thompson et al., 2013).

Koriat (1997) posits that humans have access to three types of cues that can be used to inform their metacognitive judgments. First, humans can evaluate *intrinsic* cues about the task itself, such as how difficult a certain type of word is to memorize. Second, humans can observe *extrinsic* cues about the environment or context in which the task was completed. For example, an individual may realize that they are more likely to recall a word learned at the end of a sequence than the middle (i.e., the recency effect; Castel, 2008). Finally, humans can observe *mnemonic* cues that reflect their internal experience while completing the task, such as how fluently they were able to recall a piece of relevant information or how uncomfortable they felt while completing the task (Ackerman & Thompson, 2017; Alter & Oppenheimer, 2009; Thompson et al., 2013). Although these cues are not always valid (Ackerman & Zalmanov, 2012; Finn & Tauber, 2015), they allow humans to make metacognitive judgments that are reasonably accurate (Cash & Oppenheimer, 2024; Oppenheimer, 2008; Thompson et al., 2011).

To our knowledge, there is no empirical evidence exploring the degree to which generative AI chatbots can take advantage of each type of metacognitive cue. However, Cash and Oppenheimer (2024) make a theoretical argument that generative AI chatbots almost certainly have access to *intrinsic* cues, which can be learned through statistical relationships in the training set (Wolfram, 2023; Wu et al., 2023), and almost certainly do not have access to *mnemonic* cues, which rely on experiential feelings that artificial agents do not have (Alter & Oppenheimer, 2009; Li et al., 2024; Long, 2023; Thompson et al., 2013). It is less clear whether generative AI chatbots have access to *extrinsic* cues. Many of the *extrinsic* cues that humans can use to inform their metacognitive judgments—such as serial position effects (Castel, 2008)—may not be relevant for artificial agents. However, it is possible that generative AI chatbots could be metacognitively responsive to extrinsic constraints provided by the user’s prompt engineering, such as requests to take their time or instructions to reconsider their responses before giving final answers (Federiakin et al., 2024; Y. Wang & Zhao, 2023; White et al., 2023).

While the literature lacks a comprehensive understanding of the degree to which generative AI chatbots utilize metacognitive cues, it is theoretically plausible that they could generate meaningful metacognitive judgments through some combination of *intrinsic* and *extrinsic* cues, despite their lack of introspective capacities (Li et al., 2024; Long, 2023). Here, we focus on the accuracy with which generative AI chatbots can make one type of metacognitive judgment: confidence judgments.

### Confidence judgments

Confidence judgments are a tool used by humans to communicate uncertainty (Einhorn & Hogarth, 1978; Fleming, 2024; Harvey, 1997). They can be presented either numerically (e.g., “I’m 90% sure) or verbally (e.g., “I’m pretty sure”) and provide insight to the listener about the extent to which they should trust the information provided by the speaker (Seale-Carlisle et al., 2024). For confidence judgments to be useful, they must be accurate (even if noisy) signals of the speaker’s level of uncertainty. The metacognition literature distinguishes between two forms of accuracy: absolute and relative.

*Absolute* metacognitive accuracy (also known as calibration) refers to the extent to which a judge’s objective performance aligns with their subjective beliefs about their performance, averaged across items (Fleming & Lau, 2014; Pieschl, 2009). This is typically measured by comparing the number of items a participant believes they correctly answered with the number of items they actually answered correctly and can be assessed at the individual level or the sample level. If a judge believes they performed (or will perform) better than they did, it is referred to as overestimation, a form of overconfidence (Moore & Healy, 2008; Moore & Schatz, 2017; West & Stanovich, 1997). Decades of research suggest that, across most domains, humans are generally biased towards overconfidence (Finn & Metcalfe, 2014; Johnson & Fowler, 2011; Moore & Healy, 2008; Moore & Schatz, 2017).

*Relative* metacognitive accuracy (also known as resolution) refers to the extent to which a judge can assign higher confidence judgments to the items they are more likely to answer correctly (Fleming & Lau, 2014; Schraw, 2009). The literature suggests that relative metacognitive accuracy for confidence judgments is highly dependent on the characteristics of the task. Relative metacognitive accuracy tends to be higher for easier tasks than harder tasks (Baranski & Petrusic, 1994), lower for cognitive tasks than physical tasks (Hildenbrand & Sanchez, 2022), and similar across tasks with similar structures (Ais et al., 2016). Relative metacognitive accuracy is typically measured by assessing gamma correlations between confidence and accuracy or through signal detection theoretic approaches, such as calculating the area under the Type 2 (metacognitive) receiver operating characteristic (ROC) curve (Fleming & Lau, 2014; Higham & Higham, 2019; Maniscalco & Lau, 2012).

### LLM confidence judgments

To produce an accurate confidence judgment, an LLM would first have to generate an internal representation of uncertainty. This can be achieved through a variety of advanced statistical approaches (X. Jiang et al., 2012; Kuhn et al., 2023; Kumar et al., 2019; Liang et al., 2022; Wang, 2023; Xiao et al., 2022). In state-of-the-art generative AI chatbots, internal confidence is often assessed through token likelihoods—variables that essentially indicate how surprised the model is by the answer that was generated (Jiang et al., 2020; Kadavath et al., 2022; Steyvers et al., 2025; Xiao et al., 2022). The literature suggests that these internal representations tend to be relatively accurate predictors of task performance (Kadavath et al., 2022). However, it is unlikely that a typical user would know how to access these internal representations or how to interpret them. Indeed, Steyvers et al. (2025) find that human users have greater confidence in the accuracy of LLMs’ responses than LLMs have in their own responses, as estimated from their token likelihoods. Similarly, Colombatto and Fleming (2025) find that humans attribute greater confidence to AI systems than they do to other humans, even when the same response is provided by each sample.

These challenges highlight the need for generative AI chatbots to transform their internal representations of uncertainty into linguistic confidence judgments that can be communicated to users. Early research suggests that LLMs can generate well-calibrated linguistic confidence judgments (Lin et al., 2022; Shrivastava et al., 2023; K. Tian et al., 2023; Xiong et al., 2023). However, the literature also suggests that LLMs, like humans, tend to be overconfident (Groot & Valdenegro-Toro, 2024; Xiong et al., 2023) and that the accuracy of LLMs’ confidence judgments varies by task, with lower accuracy in tasks that require expertise (Xiong et al., 2023). Though these studies provide initial evidence that generative AI chatbots can produce reasonably accurate confidence judgments, the literature is nascent and limited in two critical ways. First and foremost, many of the extant studies have not included a human sample for comparison—thus limiting our ability to understand how generative AI chatbots’ metacognitive capacities compare to those of humans. Second, much of the extant literature has failed to systematically assess both relative and absolute metacognitive accuracy, leaving gaps in our understanding of the metacognitive capacities of generative AI chatbots (Fleming & Lau, 2014).

### Current studies

In the studies presented here, we compare the absolute and relative accuracy of confidence judgments made by four generative AI chatbots—ChatGPT, Google Gemini (formerly Bard), Claude Sonnet (Studies 4–5 only), and Claude Haiku (Studies 4–5 only)—to those of humans across five domains. Exploratory comparisons between the LLMs are provided in the Supplemental Materials. We chose these four chatbots because they are widely accessible, well-known, and have free versions accessible by the public. In each study, we used the most advanced version of each model that was available for nonpaying users at the time of data collection. All LLM conversations were conducted manually by the authors using the chatbot interface.

The uncertainty literature has identified an important distinction between types of uncertainty: aleatory versus epistemic uncertainty. Aleatory uncertainty involves making judgments for which the answer cannot be known (e.g., predicting future events). Epistemic uncertainty involves judgments for which the answer could, in theory, be known but for which the judge does not have complete knowledge (e.g., answering trivia questions). This distinction has shown to be important in human confidence judgments as they lead to different cognitive representations and strategies for information search (Fox & Ülkümen, 2011). Crucially for the current discussion, epistemic uncertainty is monitored via *mnemonic* cues (e.g., “I am struggling to bring the information to mind”), which LLMs are presumed to lack (cf. Cash & Oppenheimer, 2024). While the two types of uncertainty are not mutually exclusive, making pure dissociation quite difficult (Fox & Ülkümen, 2011), the present studies separately explore domains that involve primarily aleatory (Studies 1 and 2) and primarily epistemic (Studies 3–5) uncertainty. In Studies 1 and 2, participants assessed their confidence in tasks where they predict the outcomes of future events. In Study 3, participants provided confidence judgments about their performance in a game of Pictionary. In Studies 4 and 5, participants provided confidence judgments about their ability to answer fact-based questions.

## Study 1

### Methods

In Study 1, we asked human participants, ChatGPT-3.5, and Google Bard to predict the outcome of each National Football League (NFL) game from weeks 9–18 (November 2nd–January 7th) of the 2023–2024 season.

#### Participants

Each week, we recruited 50 American Prolific participants. A total convenience sample of 502 participants completed the study (weekly *n*s = 48–52). A power analysis indicated that 502 participants would be sufficient to detect a small (*d* =.13) overconfidence effect with 80% power. Demographics are reported in the Supplemental Materials. No participants who completed the study were excluded from the analyses.

#### Procedure

At the start of each NFL week (Tuesday), a new set of Prolific participants was recruited. Participants predicted the outcome of every NFL game in the upcoming week (12—16 games, depending on the NFL schedule) with a $0.01 bonus for each correct prediction. Games were presented in a random order. For each game, participants were told which two teams were playing, the game’s location, each team’s record, and the outcome of the teams’ previous games that season. A sample task is provided in the Supplemental Materials.

After each prediction, participants rated how likely they thought it was that the prediction would come true, on a scale of 50% (“Toss-Up”) to 100% (“Guaranteed”). After making all their predictions, participants retrospectively estimated how many of their predictions would end up being accurate, incentivized with a $0.10 accuracy bonus. Participants then completed a demographic questionnaire.

ChatGPT and Bard each completed the same tasks as the human participants once per week, on Tuesdays. Data on the outcomes of previous games was uploaded in the form of a spreadsheet, and current matchups were provided as text. The script used to generate responses from the LLMs is provided in the Supplemental Materials. ChatGPT refused to read the data spreadsheet in Week 18, so analyses from ChatGPT are reported based on Weeks 9–17. Analyses for Bard and humans are reported based on Weeks 9–18. Robustness checks excluding Week 18 generated similar results (see Supplemental Materials).

### Results

#### Absolute metacognitive accuracy

**Fig. 1:**
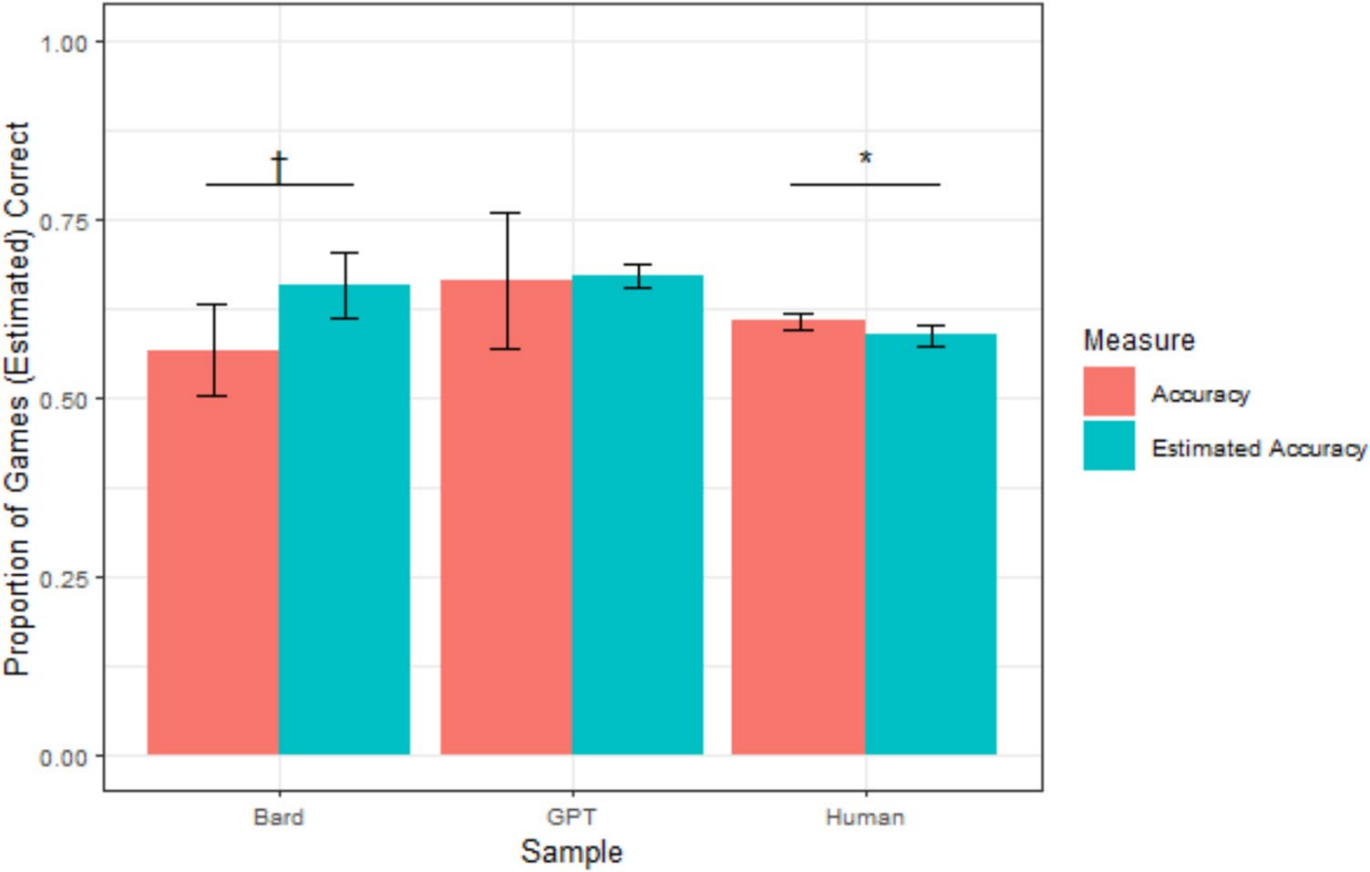
Study 1 mean accuracy and confidence by sample. *Note. *Error bars reflect 95% confidence intervals; ^†^*p* <.10; *p <.05, **p <.01, ***p <.001. (Color figure online)

Because there were a different number of games each week, we calculated accuracy and estimated accuracy rates for each participant (both humans and LLMs). These proportions were calculated by dividing the number of games a participant correctly predicted or estimated that they correctly predicted by the number of games that week. These proportions were treated as the units of analysis. Chi-squared tests evaluating total accuracy and total estimated accuracy (summed across participants/weeks) generated similar results and are reported in the Supplemental Materials. A graphical representation of each sample’s mean accuracy and confidence is depicted in Fig. 1.

On average, human participants accurately predicted 60.6% of games (*sd* = 12.6 percentage points [pp]) and estimated that they would correctly predict 58.7% of games (*sd* = 17.0 pp). A paired *t* test indicated that these two proportions were significantly different, *t*(501) = −2.20, *p =.*028, *d* = −0.10, suggesting that humans were slightly underconfident. However, measures of overconfidence do not fully capture absolute metacognitive accuracy, as they average across under- and overconfident participants, thus allowing their errors to cancel out. To more directly capture absolute metacognitive accuracy, we calculated the *calibration error* for each participant—that is, the absolute value of the distance between their accuracy and estimated accuracy. A lower calibration error suggests better absolute metacognitive accuracy. The average calibration error for humans was 15.73 percentage points (*sd* = 12.8 pp).

ChatGPT accurately predicted an average of 66.4% of games (*sd* = 12.5 pp), which was not significantly greater than human accuracy, *t*(8.30) = 1.37, *p* =.205. ChatGPT estimated that it would correctly predict 67.1% of games on average (*sd* = 2.2 pp), which was not significantly higher than its accuracy rate, suggesting that ChatGPT was well-calibrated, *t*(8) = 0.17, *p* =.870. ChatGPT’s average calibration error was 9.42 percentage points (*sd* = 7.3 pp), which was significantly lower than humans’ average calibration error, *t*(8.89) = 2.52, *p* =.03. This suggests that ChatGPT was better-calibrated than humans.

Bard accurately predicted an average of 56.7% of games across weeks (*sd* = 8.8 pp), which was not significantly different than the accuracy of humans, *t*(9.75) = −1.40, *p* =.193. Bard estimated that it would correctly predict an average of 65.7% of games (*sd* = 6.3 pp), making it marginally overconfident, *t*(9) = 2.24, *p* =.051, *d* = 0.71. Bard’s average calibration error was 11.19 percentage points (*sd* = 10.7 pp), which was not significantly different than humans’ average calibration error, *t*(9.52) = 1.32, *p* =.22. This suggests that Bard was neither better- nor worse-calibrated than humans.

#### Relative metacognitive accuracy

**Fig. 2:**
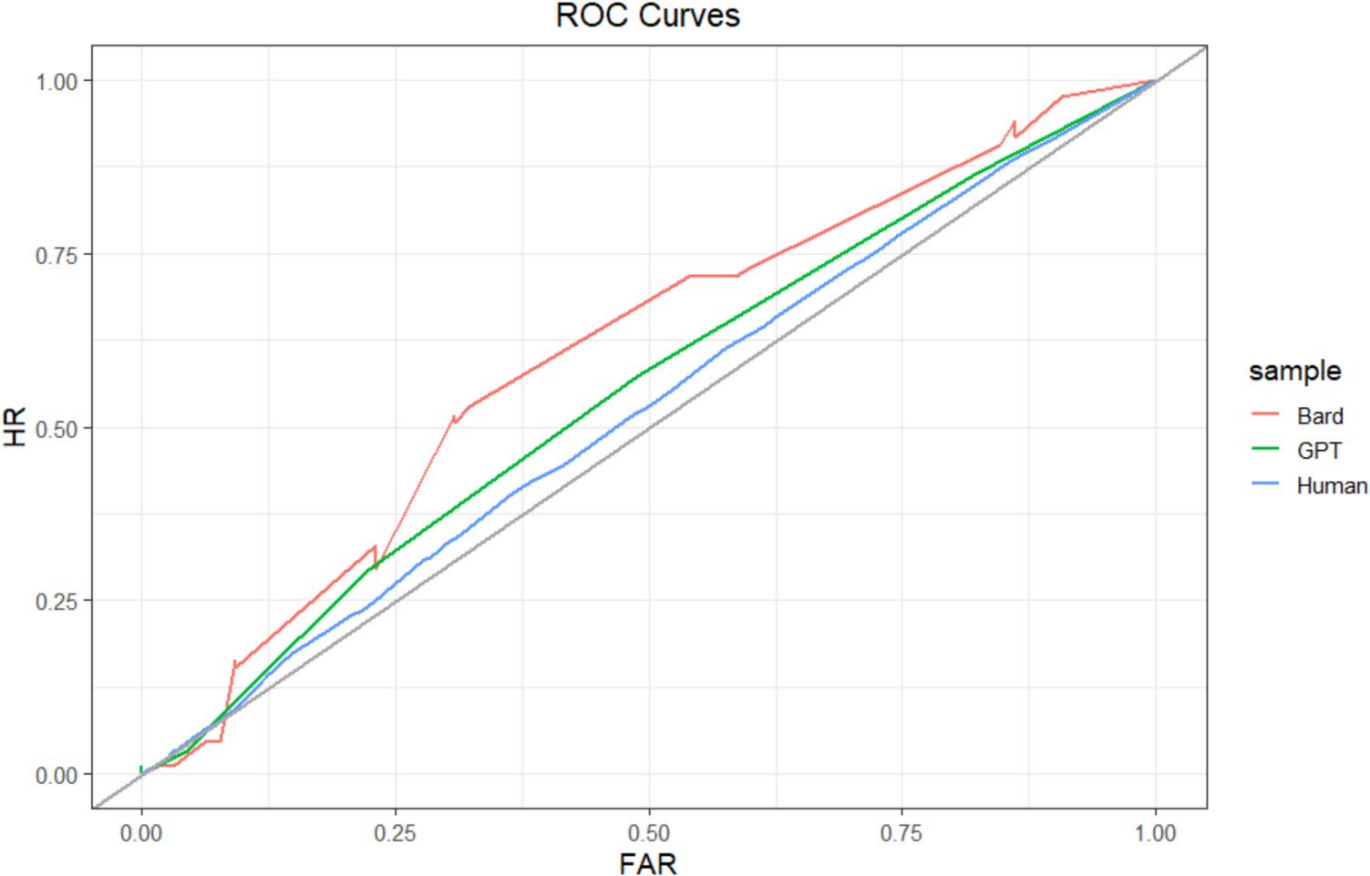
Study 1 ROC curves. Type 2 ROC curve for each sample. The gray identity line indicates chance. HR = hit rate; FAR = false-alarm rate. The curves are not completely smooth because of the relatively small number of observations. (Color figure online)

We first evaluated relative metacognitive accuracy at the sample level using principles from signal detection theory (Fleming & Lau, 2014; Higham & Higham, 2019; Maniscalco & Lau, 2012). To do so, we created a Type 2 (i.e., metacognitive) ROC curve for each sample (see Fig. 2). These curves plot the probability that correct predictions were identified as correct (i.e., the hit rate) against the probability that incorrect predictions were identified as correct (i.e., the false-alarm rate). When confidence is used as the metacognitive judgment, the points of the ROC curve are formed by determining the hit rate and false-alarm rate when confidence is at or above various confidence levels (e.g., 75%+ confidence). These points are then connected to form the ROC curve (Fleming & Lau, 2014). We generated our ROC curves using the *pROC* package in R (Robin et al., 2011).

We then calculated the area under the ROC curve (AUROC) for each sample. AUROCs function as measures of relative metacognitive accuracy, with greater values indicating higher metacognitive accuracy (Fleming & Lau, 2014; Higham & Higham, 2019). AUROC values range from 0 to 1, with 0.5 indicating random metacognitive judgments, 1.0 indicating perfect metacognitive accuracy, and 0 indicating inverted metacognitive accuracy (i.e., highest confidence when judgments are least accurate). We estimated AUROCs using the *pROC* package in R (Robin et al., 2011). AUROC is a less biased measure of relative metacognitive accuracy than correlations between accuracy and confidence, such as phi or gamma. However, it is worth noting that AUROCs, like most measures of relative metacognitive accuracy, are partially confounded by task performance (Fleming & Lau, 2014; Higham & Higham, 2019; Maniscalco & Lau, 2012).

At the sample level, humans (502 participants; 7,472 predictions; 58 missing confidence judgments) achieved an AUROC = 0.52, 95% CI [0.51, 0.54]. ChatGPT (134 predictions) achieved an AUROC = 0.55, 95% CI [0.45, 0.65]. A test for comparing the area under two ROC curves (DeLong et al., 1988) indicated that ChatGPT’s AUROC was not significantly different from humans’ (*D* = −0.52, *df* = 137.73, *p* =.60). Bard (150 predictions) achieved an AUROC = 0.60, 95% CI [0.51, 0.70], which was marginally greater than that of humans (*D* = −1.71, *df* = 155.47, *p* =.09). Sample-level ROCs are depicted in Fig. 2. Notably, each of these AUROC scores would be considered failing or poor by conventional guidelines (Nahm, 2022), suggesting that neither humans nor LLMs had high levels of relative metacognitive accuracy. Most importantly, however, we found that humans and the LLMs achieved similar levels of relative metacognitive accuracy.

As a robustness check, we reran these analyses at the participant level by calculating the AUROC for each participant and then averaging across participants. This is the more typical approach in the SDT literature (Higham & Higham, 2019), but we treated it as a secondary analysis because our participants did not complete as many trials as is typical in the SDT literature, making participant-level estimates less reliable. The average human AUROC was 0.62 (*sd* = 0.11). The average ChatGPT AUROC was 0.61 (*sd* = 0.07), which was not significantly different from humans, *t*(8.68) = 0.38, *p* =.71. The average Bard AUROC was 0.62 (*sd* = 0.09), which also was not significantly different from humans, *t*(9.48) = 0.14, *p* =.89. These results show a similar pattern to the sample-level results, but Bard’s greater accuracy than humans became nonsignificant.

### Study 1 discussion

Study 1 provided an initial evaluation of the absolute and relative metacognitive accuracy of LLMs’ confidence judgments relative to those of humans. In terms of absolute metacognitive accuracy, humans were slightly underconfident, ChatGPT was well-calibrated, and Bard was marginally overconfident. ChatGPT demonstrated better-calibrated absolute metacognitive accuracy than humans, whereas Bard was about as well-calibrated as humans. In contrast, Bard demonstrated marginally greater relative metacognitive accuracy than humans at the sample level (but not the participant level), while ChatGPT’s relative metacognitive accuracy was no different than that of humans at either level. Furthermore, we found that humans and LLMs alike failed to demonstrate high levels of relative metacognitive accuracy, perhaps reflecting the difficulty of the task itself. Overall, these results suggest that LLMs’ confidence judgments were not consistently more or less accurate than those of humans. However, results should also be interpreted with some caution, as LLMs were only prompted once per week, resulting in a small number of trials. In Study 2, we generalize these effects to another prediction domain: choosing Oscar winners.

## Study 2

### Methods

Study 2 was preregistered on AsPredicted. The preregistration adheres to the disclosure requirements of AsPredicted. All analyses were preregistered, unless noted. The preregistration can be found online (https://aspredicted.org/T1L_2J4).

#### Participants

A power analysis indicated that we would need 90 human participants to detect a small-medium overconfidence effect (*d* =.30) with 80% power. Rounding up, we sought data from 100 participants. To achieve this, we recruited a convenience sample of 110 American Prolific participants, 106 of whom completed the study (80% power to detect *d* =.28). This was a slight deviation from our preregistration, which suggested we would only recruit 100 participants. Demographics are reported in the Supplemental Materials. No participants who completed the study were excluded from analyses.

#### Procedure

All data (human and LLM) was collected on March 9, 2024, in anticipation of the Oscars (an American award show recognizing excellence in film) taking place on March 10, 2024.

Participants predicted the winner of nine Oscars categories (presented in a random order) and received a $0.02 bonus for each correct prediction. Each category had five nominees from which the participant could pick. We chose nine categories that were likely to be understood by a general audience (e.g., Best Actor) and excluded categories that were less accessible (e.g., Best Sound Design). For each category, participants were given a summary of the film, Rotten Tomatoes scores, box office revenue, and one additional category-specific piece of information (e.g., previous nominations for Best Actor nominees). A sample task is provided in the Supplemental Materials.

After each prediction, participants rated how likely they thought it was that the prediction would come true, on a scale of 20% (“Random Guess”) to 100% (“Guaranteed”). After making all their predictions, participants estimated how many of their predictions would end up being accurate (out of 9), incentivized with a $0.10 accuracy bonus. Participants then completed a demographic questionnaire.

ChatGPT-3.5 and Gemini 1.0 Ultra each completed the same tasks as the human participants 15 times. All data were uploaded to the LLMs as text. The script used to generate responses from the LLMs is provided in the Supplemental Materials.

### Results

#### Absolute metacognitive accuracy

On average, human participants accurately predicted the winners of 3.65 categories (*sd* = 1.60). They estimated that they would correctly predict an average of 4.29 categories (*sd* = 1.86). A paired *t* test indicated that these two proportions were significantly different, *t*(105) = 3.23, *p =.*002, *d* = 0.31, suggesting that humans were overconfident. The average calibration error for humans was 1.66 categories (*sd* = 1.34).

ChatGPT accurately predicted an average of 5.20 categories (*sd* = 1.37), which was significantly greater than the human participants, *t*(19.83) = 4.00, *p* =.001. ChatGPT estimated that it would correctly predict an average of 5.83 categories (*sd* = 0.45), making it slightly overconfident on average, but a paired *t* test indicated that the difference was not significant, *t*(14) = 1.70, *p* =.111. However, the effect size of ChatGPT’s overconfidence (*d* = 0.44) was similar to—and slightly larger than—the effect size of humans’ overconfidence (*d* = 0.31), suggesting that we were underpowered to detect ChatGPT’s overconfidence. ChatGPT’s average calibration error was 1.30 categories (*sd* = 0.84), which was not significantly different than humans’ average calibration error, *t*(25.51) = 1.42, *p* = 0.17. This suggests that ChatGPT was neither better nor worse calibrated than humans.

**Fig. 3:**
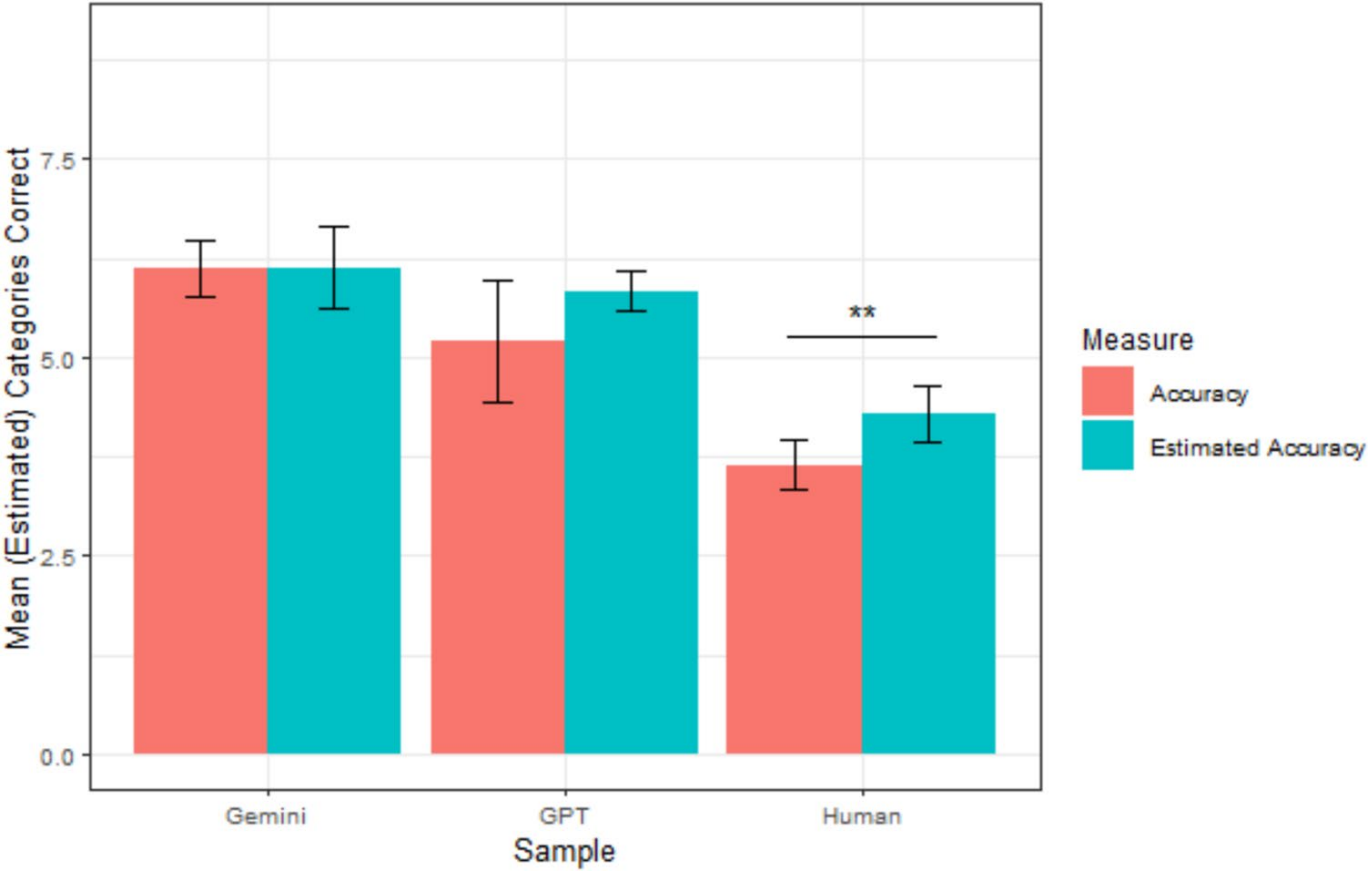
Study 2 mean accuracy and confidence by sample. *Note. *Error bars reflect 95% confidence intervals; ^†^*p* <.10; *p <.05, **p <.01, ***p <.001. (Color figure online)

Gemini accurately predicted an average of 6.13 categories (*sd* = 0.64), which was significantly greater than the average accuracy of the human participants, *t*(45.19) = 10.93, *p* <.001. Gemini estimated that it would correctly predict 6.13 categories on average (*sd* = 0.95), making it perfectly calibrated, *t*(14) = 0.00, *p* = 1.00. Gemini’s average calibration error was 0.80 categories (*sd* = 0.77), which was significantly lower than humans, *t*(27.81) = 3.60, *p* =.001. This suggests that Gemini was better calibrated than humans. A graphical representation of each sample’s mean accuracy and confidence is depicted in Fig. 3.

#### Relative metacognitive accuracy

**Fig. 4:**
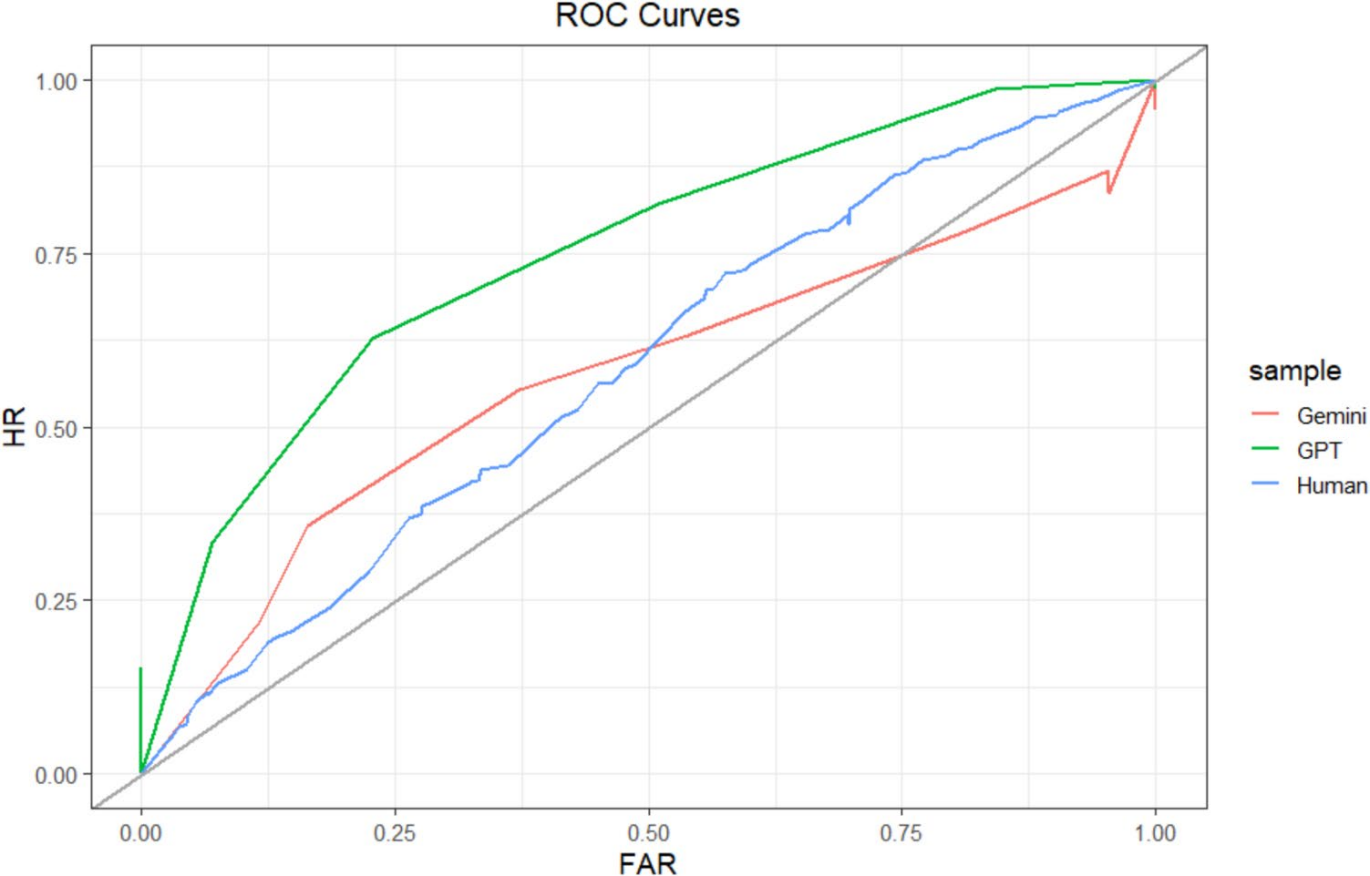
Study 2 ROC Curves. Type 2 ROC curve for each sample. The gray identity line indicates chance. HR = hit rate. FAR = false-alarm rate. The curves are not completely smooth because of the relatively small number of observations. (Color figure online)

We assessed relative metacognitive accuracy using the same methods as in Study 1. We initially preregistered that we would also analyze gamma correlations, but in response to reviewer feedback have not included those analyses. At the sample level, humans (106 participants; 954 predictions) achieved an AUROC of 0.58, 95% CI [0.55, 0.62]. ChatGPT (135 predictions) achieved an AUROC = 0.75, 95% CI [0.67, 0.83]. A test for comparing the area under two ROC curves (DeLong et al., 1988) indicated that ChatGPT’s AUROC was significantly greater than humans’ (*D* = −3.82, *df* = 195.80, *p* <.001). Gemini (135 predictions) achieved an AUROC = 0.58, 95% CI [0.48, 0.68], which was no different than that of humans (*D* = 0.16, *df* = 174.6, *p* =.87). Sample-level ROCs are depicted in Fig. 4. The AUROCs for Gemini and humans would be considered failing, whereas the AUROC for ChatGPT would be considered fair.

When assessed at the participant level, the average human AUROC was 0.68 (*sd* = 0.15). The average ChatGPT AUROC was 0.79 (*sd* = 0.12), which was significantly higher than humans, *t*(21.22) = −3.52, *p* =.002. The average Gemini AUROC was 0.63 (*sd* = 0.11), which was not significantly different from humans, *t*(21.05) = 1.45, *p* =.16. These results showed a similar pattern to the sample-level results, with ChatGPT being more accurate than humans and Gemini being about as accurate as humans.

### Study 2 discussion

Study 2 demonstrated that, compared to humans, ChatGPT showed similar absolute metacognitive accuracy, but significantly higher relative metacognitive accuracy. Gemini showed greater absolute accuracy than humans, but similar relative accuracy. Only humans were overconfident. Together, these results support the hypothesis that—despite their metacognitive limitations—LLMs can generate confidence estimates that are about as accurate as those of humans, and in some cases more accurate. Results for the LLMs should be interpreted with some caution due to the relatively low sample sizes. In Study 3, we generalize our findings to a domain where participants are asked not to predict an exogenous future event, but to predict and assess their own performance in a game of Pictionary. This provides a highly different domain, rooted in epistemic rather than aleatory uncertainty, in which to assess the robustness of our results. We also increase the sample size for LLMs and the number of trials to increase the power of our analyses.

## Study 3

### Methods

Study 3 was preregistered on AsPredicted. The preregistration adheres to the disclosure requirements of AsPredicted. All analyses were preregistered, except where noted. The preregistration can be found online (https://aspredicted.org/BJP_BZX).

#### Participants

We sought to recruit a minimum of 150 participants so that we would have sufficient responses (*n* = 30) to normalize data for each of five artists who created sketches for the game (see Stimuli). In total, a convenience sample of 165 American Prolific participants completed the study, but one was excluded for providing an answer for only one sketch, leaving a total of 164 participants (80% power to detect *d* =.22). Demographics are reported in the Supplemental Materials.

#### Procedure

Participants played a Pictionary-like game in which they were shown 25 sketches by the same artist and guessed what the drawings were meant to depict, with a time limit of 15 s per sketch. Participants first completed a practice round in which they made guesses for five drawings, with feedback about the correct answer. They then completed the main round, which contained 20 more drawings, without feedback. Participants earned a $0.01 bonus for each correct guess in the main round.

Participants were asked to rate how likely they thought it was that each guess was correct on a scale of 0% (“No Chance”) to 100% (“Guaranteed”). After the practice round, participants made a *prospective* overall confidence judgment in which they predicted how many of the 20 drawings they thought they would get right in the main round. After the main round, they made a *retrospective* overall confidence judgment estimating how many of the 20 drawings they thought they had correctly guessed in the main round. Each overall confidence judgment was incentivized with a $0.10 accuracy bonus.

We introduced the *prospective* confidence judgments so that we could assess changes in participants’ confidence before and after completing the task. Because humans’ metacognitive judgments are informed by their experiences (i.e., mnemonic cues), retrospective confidence judgments are often more accurate than prospective confidence judgments, reflecting a sort of metacognitive learning (Fleming et al., 2016; Siedlecka et al., 2016). Including both confidence judgments allowed us to test whether the same was true for LLMs.

ChatGPT-4 and Gemini 1.5 Flash were each asked to complete the same tasks as the human participants 30 times. Drawings were uploaded to the LLMs as images. This presented an interesting challenge for LLMs, since image recognition is a relatively newer capacity for LLMs compared to pure language processing. However, there is great interest in the AI literature in using LLMs to interpret a wide variety of visual inputs, including medical images (D. Tian et al., 2024), weather maps (Takasuka et al., 2024), and faces (Deandres-Tame et al., 2024), thus highlighting the importance of evaluating LLMs’ confidence in their ability to do so. The script used to generate responses from the LLMs is provided in the Supplemental Materials. All LLM data was collected between May 16–June 4, 2024.

#### Stimuli

**Fig. 5:**
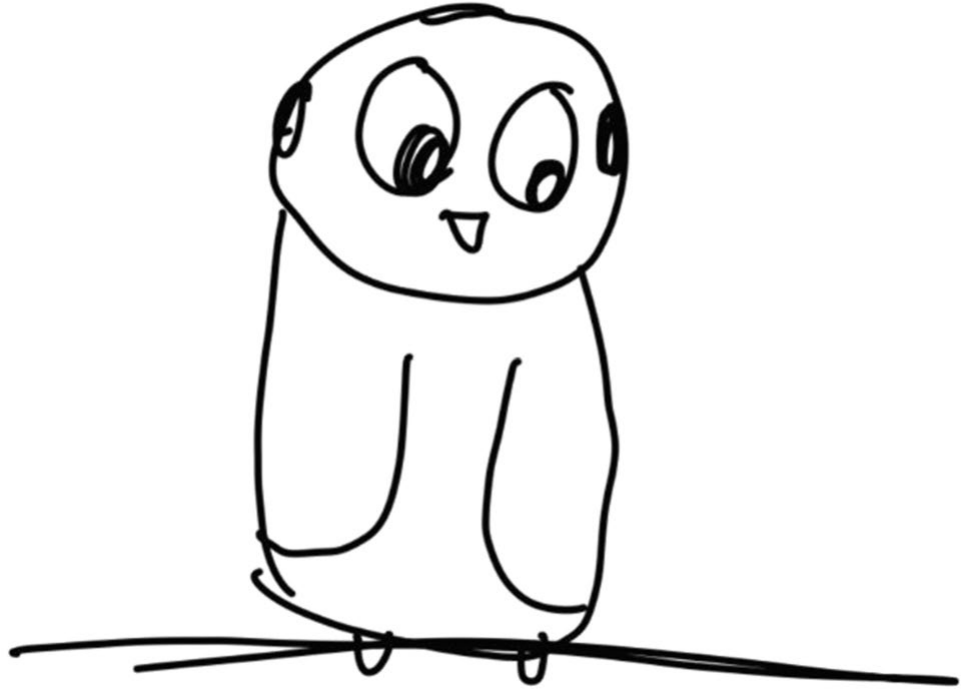
Sample sketch (drawn by Artist 2) depicting an owl

Sketches were generated by five nonprofessional artists who were given a series of words and allotted 20 s for each drawing. Sketches were hand drawn, then scanned into a digital format. The words were generated using a Random Word Generator for Pictionary, set to generate 12 easy-difficulty words and 13 medium-difficulty words (*Pictionary *Generator, 2024). Word-level mean accuracy and confidence are reported in the Supplemental Materials. Participants were randomly assigned to one of five artists. All participants saw sketches depicting the same 25 words. Within rounds, sketch order was randomized. A sample drawing is provided in Fig. 5.

### Results

We evaluated performance only in the Main Round. Undergraduate RAs coded participant responses (Human and LLM) as correct or incorrect. The coding guide is provided in the Supplemental Materials. Coders agreed on 99.6% of codes (*k* =.99). Disagreements (*n* = 19) were resolved by the first author.

#### Absolute metacognitive accuracy

On average, human participants made accurate guesses for 12.38 sketches (*sd* = 2.27). Prospectively, they predicted that they would make accurate guesses for an average of 13.10 sketches (*sd* = 3.50). Retrospectively, the average estimate was 12.87 sketches (*sd* = 2.97). Paired *t* tests indicated that humans were overconfident both prospectively, *t*(163) = 2.59, *p =.*011, *d* =.20, and retrospectively, *t*(163) = 2.09, *p =.*038, *d* =.16. Humans’ prospective average calibration error was 2.95 sketches (*sd* = 2.11), and their retrospective average calibration error was 2.36 (*sd* = 1.82). Their retrospective calibration was significantly better than their prospective calibration, *t*(163) = −3.05, *p* =.003, suggesting that they gained metacognitive insight from their experience during the task.

ChatGPT made accurate guesses for an average of 12.50 sketches (*sd* = 1.53), which was not significantly different than the human participants, *t*(55.72) = 0.35, *p* =.727. Prospectively, ChatGPT predicted that it would correctly guess an average of 13.23 sketches (*sd* = 1.65). Retrospectively, ChatGPT estimated that it correctly guessed an average of 16.07 sketches (*sd* = 1.41). Paired *t* tests indicated that ChatGPT was overconfident both prospectively, *t*(29) = 2.08, *p =.*046, and retrospectively, *t*(29) = 10.55, *p <.*001. ChatGPT’s prospective average calibration error was 1.47 (*sd* = 1.43), which was lower than its retrospective average calibration error of 3.67 (*sd* = 1.85). *t*(29) = 6.93, *p* <.001. ChatGPT’s average prospective calibration error was lower than humans’, *t*(55.06) = 4.80, *p* <.001, but its average retrospective calibration error was higher than humans’, *t*(39.94) = −3.29, *p* =.002—indicating better prospective accuracy, but worse retrospective accuracy.

Gemini made accurate guesses for 0.93 sketches on average (*sd* = 0.91), which was significantly worse than the average accuracy of the human participants, *t*(108.47) = −47.16, *p* <.001. Prospectively, Gemini predicted that it would correctly guess an average of 10.03 sketches (*sd* = 2.40). Retrospectively, Gemini estimated that it made accurate guesses for an average of 14.40 sketches (*sd* = 2.33). Paired *t* tests indicated that Gemini was overconfident both prospectively, *t*(29) = 17.83, *p <.*001, *d* = 3.25, and retrospectively, *t*(29) = 27.06, *p <.*001, *d* = 4.94. Gemini’s prospective average calibration error was 9.10 (*sd* = 2.80), which was lower than its retrospective average calibration error of 13.47 (*sd* = 2.73), *t*(29) = 12.34, *p* <.001. Gemini’s average calibration error was greater than humans’ both prospectively, *t*(35.29) = 11.46, *p* <.001, and retrospectively, *t*(33.88) = 21.46, *p* <.001, indicating worse absolute accuracy.

**Fig. 6:**
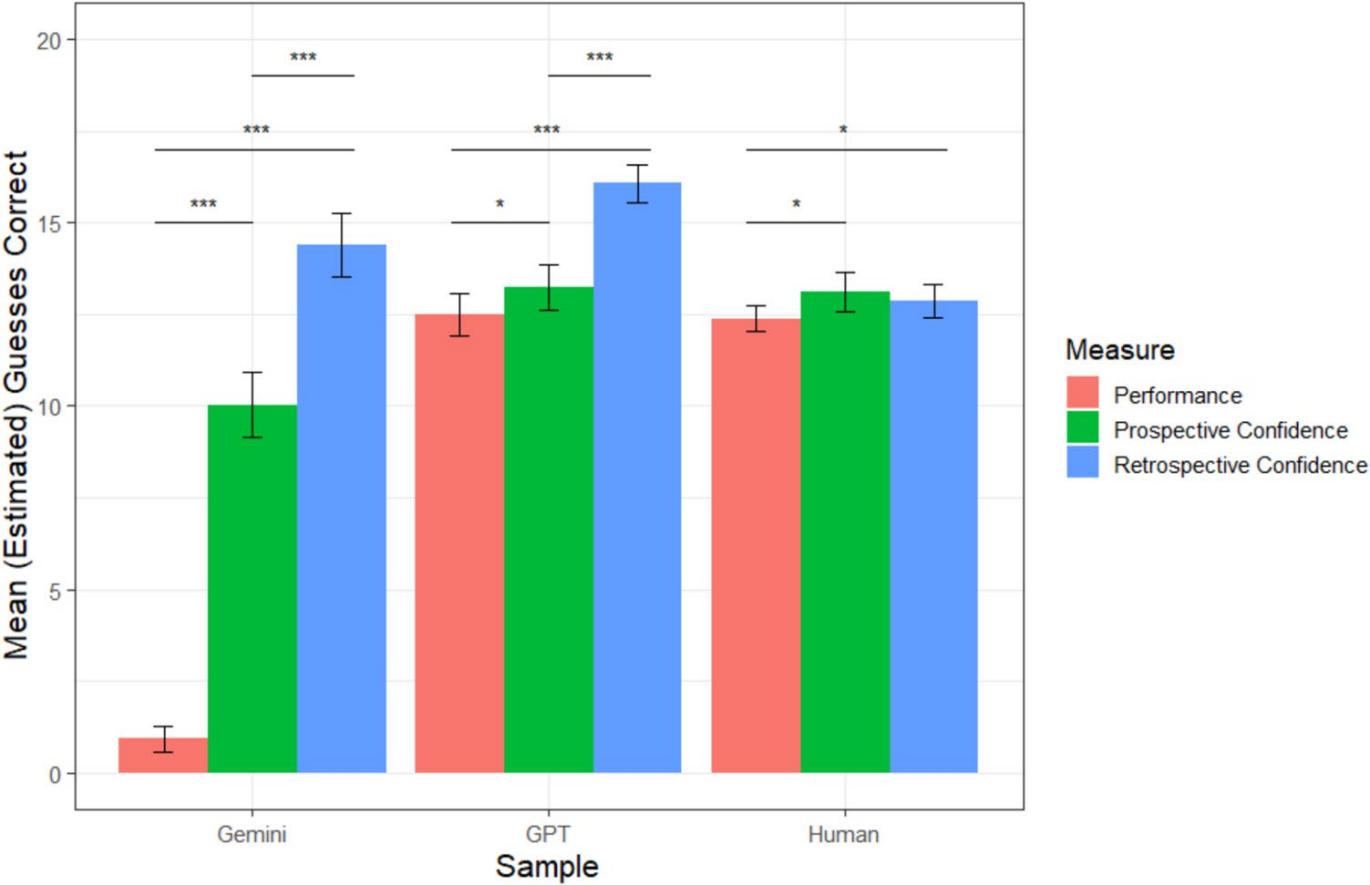
Study 3 mean accuracy and confidence by sample. *Note. *Error bars reflect 95% confidence intervals; ^†^*p* <.10; *p <.05, **p <.01, ***p <.001. (Color figure online)

A graphical representation of each sample’s mean accuracy, prospective confidence, and retrospective confidence is depicted in Fig. 6. Of note, we originally preregistered that we would compare all samples to one another using analyses of variance (ANOVAs), but decided to use *t* tests as our focus was comparing LLMs to humans, not one another.

#### Relative metacognitive accuracy

**Fig. 7:**
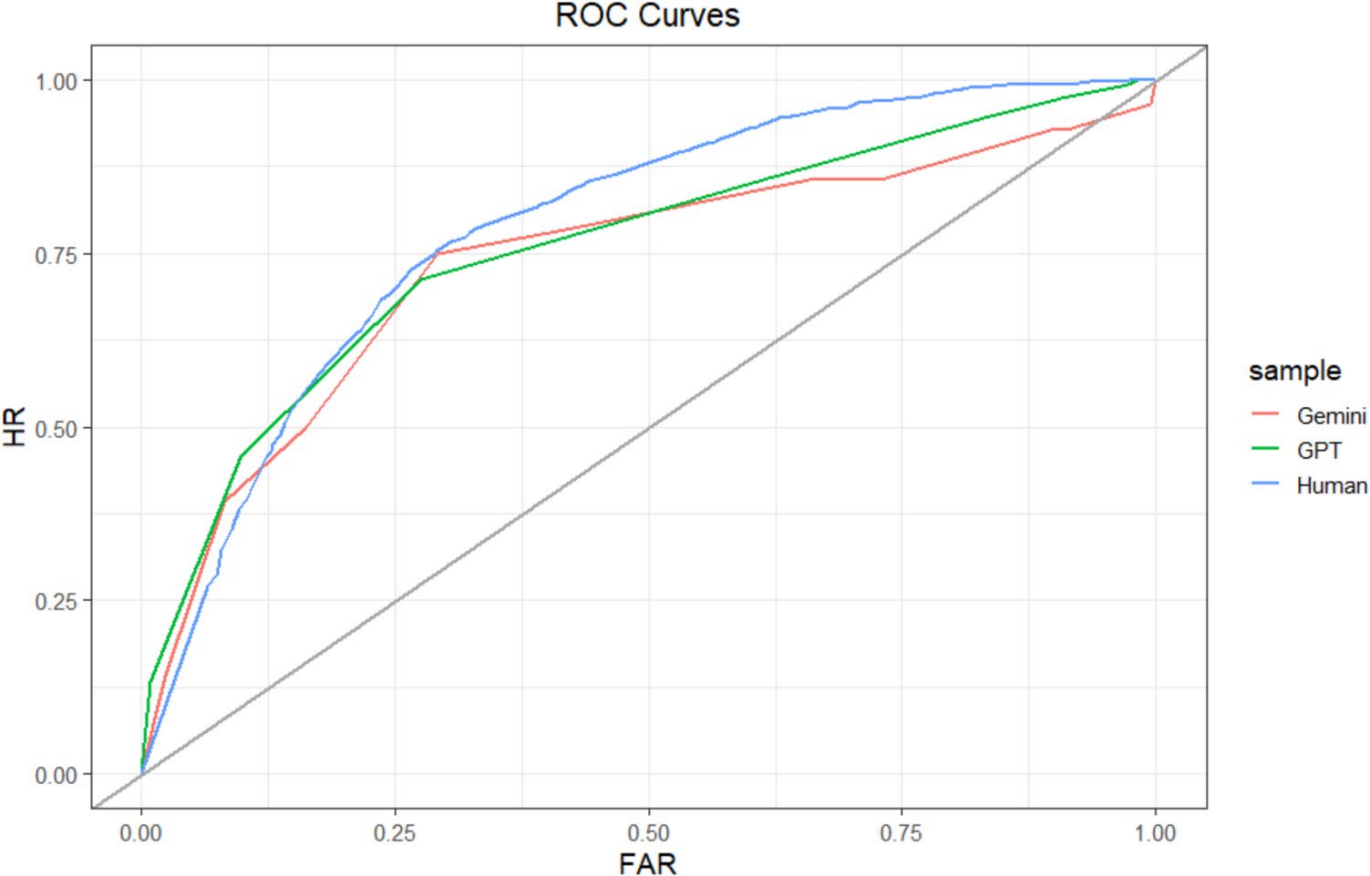
Study 3 ROC curves. Type 2 ROC curve for each sample. The gray identity line indicates chance. HR = hit rate; FAR = false-alarm rate. The curves are not completely smooth because of the relatively small number of observations. (Color figure online)

We assessed relative metacognitive accuracy using the same methods as in Studies 1 and 2. We initially preregistered that we would analyze gamma correlations, but in response to reviewer feedback instead report AUROCs. At the sample level, humans (164 participants; 3,218 guesses, 62 missing confidence judgments) achieved an AUROC of 0.79, 95% CI [0.77, 0.80]. ChatGPT (600 guesses) achieved an AUROC = 0.76, 95% CI [0.72, 0.79]. ChatGPT’s AUROC was no different than humans’ (*D* = 1.49, *df* = 844.98, *p* =.14). Gemini (600 guesses) achieved an AUROC = 0.74, 95% CI [0.62, 0.85], which was no different than that of humans (*D* = 0.91, *df* = 625.34, *p* =.37). Sample-level ROCs are depicted in Fig. 7. These results indicate that all samples had fair (and approaching good) relative metacognitive accuracy, and that accuracy was similar across each sample. However, the results for Gemini should be interpreted with skepticism due to its poor task accuracy.

When assessed at the participant level, the average human AUROC was 0.83 (*sd* = 0.11). The average ChatGPT AUROC was 0.77 (*sd* = 0.10), which was significantly lower than for humans, *t*(43.72) = 2.84, *p* =.01. The average Gemini AUROC was 0.83 (*sd* = 0.15), which was not significantly different from humans. *t*(20.53) = −0.16, *p* =.87. However, it is worth noting 11/30 Gemini participants had to be excluded because they answered zero items correctly, resulting in null ROC curves. The participant-level results are slightly different from the sample-level metrics, as they suggest that humans have slightly greater relative metacognitive accuracy than ChatGPT.

#### Item-level comparisons

We next conducted exploratory analyses in which we compared each sample’s mean accuracy, confidence, and overconfidence for each word, averaged across artists. These analyses allowed us to investigate whether humans and LLMs performed similarly on the same items. Humans’ word-level accuracy was strongly correlated with ChatGPT’s word-level accuracy (*r* =.87, *p* <.001) and moderately correlated with Gemini’s word-level accuracy (*r* =.52, *p* =.02). Word-level accuracy for the two LLMs was moderately correlated (*r* =.59, *p* = 006). Humans’ word-level confidence was strongly correlated with ChatGPT’s word-level confidence (*r* =.88, *p* <.001) and moderately correlated with Gemini’s (*r* =.48, *p* =.03). Word-level confidence was also moderately correlated between the two LLMs (*r* =.45, *p* =.045). Humans’ word-level overconfidence was strongly correlated with ChatGPT’s word-level overconfidence (*r* =.91, *p* <.001) and marginally correlated with Gemini’s (*r* =.41, *p* =.07). There was also a moderate correlation between word-level overconfidence for the two LLMs (*r* =.51, *p* =.02). Together, these results suggest that humans, ChatGPT, and Gemini found the same items difficult, were more confident on the same items, and were well-calibrated (or poorly calibrated) on the same items, suggesting some cognitive and metacognitive similarities.

### Study 3 discussion

Study 3 revealed several interesting trends. First, Gemini was terrible at the task itself—whereas humans and ChatGPT performed significantly better. In terms of absolute metacognitive accuracy, all samples were overconfident both prospectively and retrospectively. Gemini’s absolute metacognitive accuracy was much worse than that of humans. Interestingly, ChatGPT was better-calibrated than humans prospectively, but worse-calibrated than humans retrospectively. This was largely because humans became better-calibrated retrospectively than prospectively, while ChatGPT and Gemini became less well-calibrated retrospectively than prospectively. This suggests that humans have a unique ability to learn about their own performance that is not captured by LLMs, perhaps driven by their access to mnemonic metacognitive cues (Ackerman & Thompson, 2017; Alter & Oppenheimer, 2009; Cash & Oppenheimer, 2024; Thompson et al., 2013). Sample-level relative metacognitive accuracy was high for all samples and not significantly different across samples. Participant-level relative metacognitive accuracy was also high for all samples, and slightly higher for humans than ChatGPT. In Study 4, we test the absolute and relative metacognitive accuracy of LLMs in a text-based trivia game, which may be more reflective of typical LLM use cases.

## Study 4

### Methods

Study 4 was preregistered on AsPredicted. The preregistration adheres to the disclosure requirements of AsPredicted. All analyses were preregistered, except where noted. The preregistration can be found online (https://aspredicted.org/f33r-z39k.pdf).

#### Participants

A power analysis indicated that we would need a minimum of 90 participants to have 80% power to detect a small to medium effect size for a paired *t* test (*d* =.30). To ensure sufficient power, we rounded our target sample size up to 100. To account for potential data quality errors, we recruited 110 American Prolific participants to complete this study. All 110 participants completed the study, and no participants were excluded. Demographics are reported in the Supplemental Materials.

#### Stimuli

The research team created a novel set of 25 general knowledge trivia questions that asked highly specific questions about familiar concepts in popular culture (e.g., sports, history, video games). These questions were designed to be difficult to find the answer for on the internet—requiring the participant to integrate information from multiple websites or synthesize multiple pieces of information from an individual website. This was done to prevent the LLMs (or the humans) from simply looking up the answers and having a near-perfect accuracy rate. For example, one question asked: “522 Major League Baseball players hit at least 1 home run in the 2024 season. How many hit exactly 1 home run (no more and no less)?” Finding the answer to this question would require locating the dataset (Major League Baseball, 2025), sorting by number of home runs, excluding all participants who hit more than one home run, and counting individual players.

Each question had a numeric correct answer. To make the questions feasible for humans, participants (both human and LLM) were given a value and asked to guess whether the true value was “More Than” or “Fewer Than” the provided number. In the home run question, for example, the number provided was 75 and the true value was 69, so the correct answer was “Fewer than 75.” Participants were randomly assigned to one of four randomized question orders. The full set of questions—along with question-level mean accuracy and confidence—is provided in the Supplemental Materials.

#### Procedure

Participants played a trivia game in which they were asked to answer the 25 general knowledge questions described above. Participants first completed a practice round in which they answered three example questions, with feedback about the correct answer. They then completed the main round, which contained 22 more questions, without feedback. Participants earned a $0.01 bonus for each correct guess in the main round.

Participants were asked to rate how likely they thought it was that each guess was correct, on a scale of 50% (“Random Guess”) to 100% (“Certain”). After the practice round, participants made a *prospective* overall confidence judgment in which they predicted how many of the 22 questions they thought they would get right in the main round. After the main round, they made a *retrospective* overall confidence judgment estimating how many of the 22 questions they thought they had correctly guessed in the main round. Each overall confidence judgment was incentivized with a $0.10 accuracy bonus.

ChatGPT-4o, Gemini 1.5 Flash, Claude Sonnet 3.5, and Claude Haiku 3.5 were each asked to complete the same tasks as the human participants 100 times. The script used to generate responses from the LLMs is provided in the Supplemental Materials. All LLM data was collected between January 17–February 11, 2025.

### Results

We evaluated performance only in the Main Round.

#### Absolute metacognitive accuracy

On average, human participants correctly answered 11.52 questions (*sd* = 2.17). Prospectively, they predicted that they would correctly answer 11.15 questions (*sd* = 3.78). Retrospectively, the average estimate was 9.69 questions (*sd* = 3.78). Paired *t* tests indicated that humans were well-calibrated prospectively, *t*(109) = −0.81, *p =.*42, and underconfident retrospectively, *t*(109) = −4.28, *p <.*001, *d* =.41. Humans’ prospective average calibration error was 3.51 questions (*sd* = 3.15) and their retrospective average calibration error was 3.72 questions (*sd* = 3.08). Their retrospective calibration was not significantly different than their prospective calibration, *t*(109) = 0.80, *p* =.42.

ChatGPT correctly answered 13.81 questions on average (*sd* = 1.96), which was significantly higher than the human participants, *t*(208) = 8.04, *p* <.001. Prospectively, ChatGPT predicted that it would correctly answer an average of 17.14 questions (*sd* = 0.88). Retrospectively, ChatGPT estimated that it correctly answered an average of 17.21 questions (*sd* = 0.66). Paired *t* tests indicated that ChatGPT was overconfident both prospectively, *t*(99) = 15.48, *p* <.001, *d* = 1.55, and retrospectively, *t*(99)= 16.87, *p <.*001, *d* = 1.69. ChatGPT’s prospective average calibration error was 3.39 questions (*sd* = 2.05), which was not significantly different than its retrospective average calibration error of 3.42 questions (*sd* = 1.98), *t*(99) = 0.40, *p* =.69. ChatGPT’s average calibration error was not different than that of humans either prospectively, *t*(189.33) = 0.33, *p* =.74, or retrospectively, *t*(187.8) = 0.84, *p* =.40, suggesting similar levels of absolute metacognitive accuracy.

Gemini correctly answered 9.82 questions on average (*sd* = 1.06), which was significantly worse than the average accuracy of the human participants, *t*(161.45) = −7.32, *p* <.001. Prospectively, Gemini predicted that it would correctly answer an average of 14.76 questions (*sd* = 1.02). Retrospectively, Gemini estimated that it correctly answered an average of 16.47 questions (*sd* = 1.26). Paired *t* tests indicated that Gemini was overconfident both prospectively, *t*(99) = 34.96, *p <.*001, *d* = 3.50, and retrospectively, *t*(99) = 40.06, *p <.*001, *d* = 4.01. Gemini’s prospective average calibration error was 4.94 questions (*sd* = 1.41), which was significantly lower than its retrospective average calibration error of 6.65 questions (*sd* = 1.66), *t*(99) = 14.35, *p* <.001. Gemini’s average calibration error was greater than that of humans both prospectively, *t*(154.34) = 4.31, *p* <.001, and retrospectively, *t*(170.49) = 8.68, *p* <.001, suggesting worse absolute metacognitive accuracy.

Sonnet correctly answered 16.16 questions on average (*sd* = 1.21), which was significantly better than the average accuracy of the human participants, *t*(174.25) = 19.38, *p* <.001. Prospectively, Sonnet predicted that it would correctly answer an average of 14.11 questions (*sd* = 0.62). Retrospectively, Sonnet estimated that it correctly answered an average of 15.06 questions (*sd* = 0.81). Paired *t* tests indicated that Sonnet was underconfident both prospectively, *t*(99) = −15.43, *p <.*001, *d* = −1.54, and retrospectively, *t*(99) = −7.84, *p <.*001, *d* = −0.78. Sonnet’s prospective average calibration error was 2.15 questions (*sd* = 1.41), which was significantly higher than its retrospective average calibration error of 1.44 questions (*sd* = 1.05), *t*(99) = −6.91, *p* <.001. Sonnet’s average calibration error was lower than that of humans both prospectively, *t*(140.46) = −4.23, *p* <.001, and retrospectively, *t*(136.02) = −7.30, *p* <.001, suggesting better absolute accuracy.

**Fig. 8:**
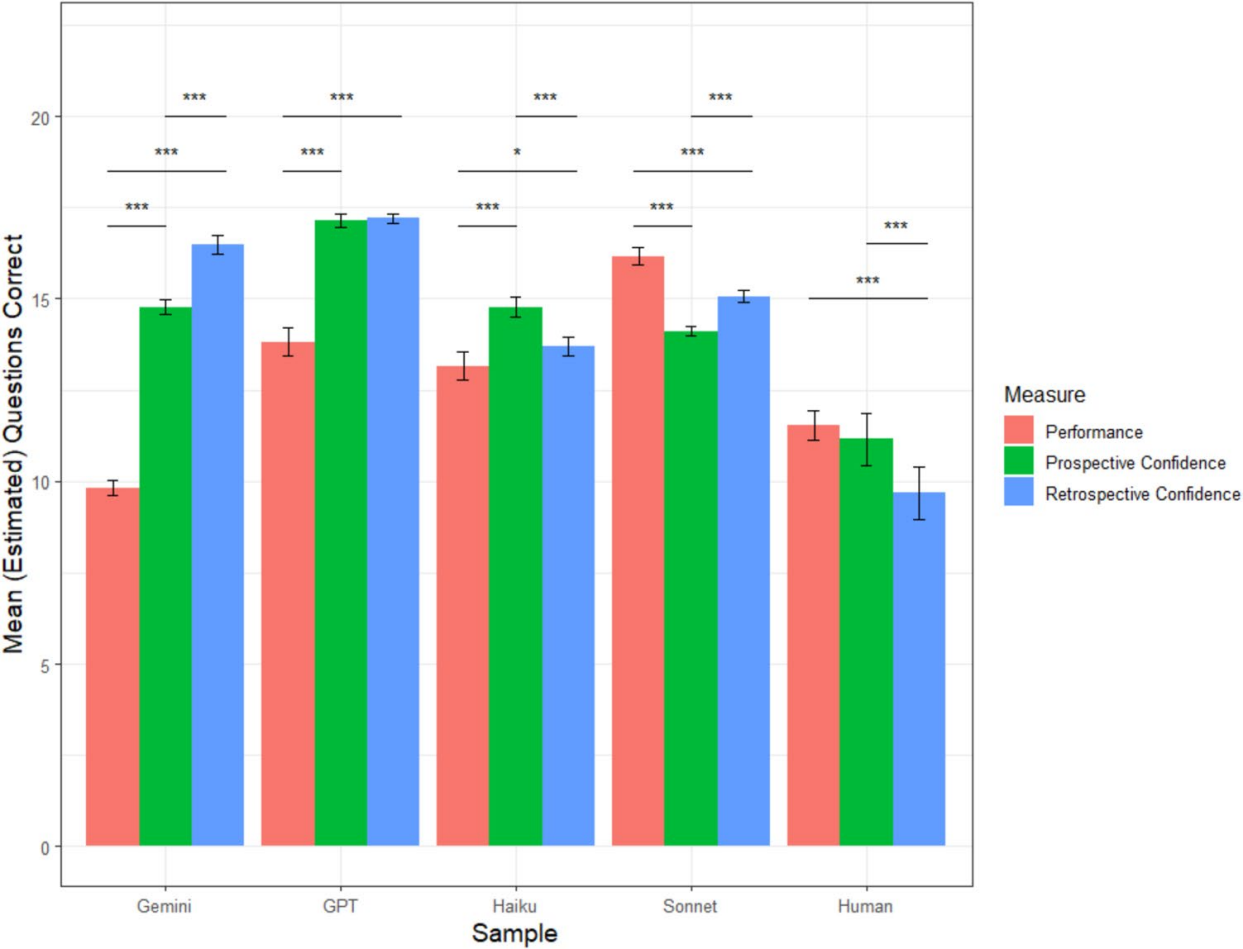
Study 4 mean accuracy and confidence by sample. *Note. *Error bars reflect 95% confidence intervals; ^†^*p* <.10; *p <.05, **p <.01, ***p <.001. (Color figure online)

Haiku correctly answered 13.16 questions on average (*sd* = 1.96), which was significantly better than the average accuracy of the human participants, *t*(207.99) = 5.77, *p* <.001. Prospectively, Haiku predicted that it would correctly answer an average of 14.76 questions (*sd* = 1.37). Retrospectively, Haiku estimated that it correctly answered an average of 13.69 questions (*sd* = 1.22). Paired *t* tests indicated that Haiku was overconfident both prospectively, *t*(99) = 6.14, *p <.*001, *d* = 0.61, and retrospectively, *t*(99) = 2.14, *p =.*04, *d* = 0.21. Haiku’s prospective average calibration error was 2.50 questions (*sd* = 1.75), which was significantly higher than its retrospective average calibration error of 2.07 questions (*sd* = 1.45), *t*(99) = −3.97, *p* <.001. Haiku’s average calibration error was lower than that of humans both prospectively, *t*(173.61) = −2.91, *p* =.004, and retrospectively, *t*(158.25) = −5.03, *p* <.001, suggesting better absolute accuracy. A graphical representation of each sample’s mean accuracy, prospective confidence, and retrospective confidence is depicted in Fig. 8.

#### Relative metacognitive accuracy

**Fig. 9:**
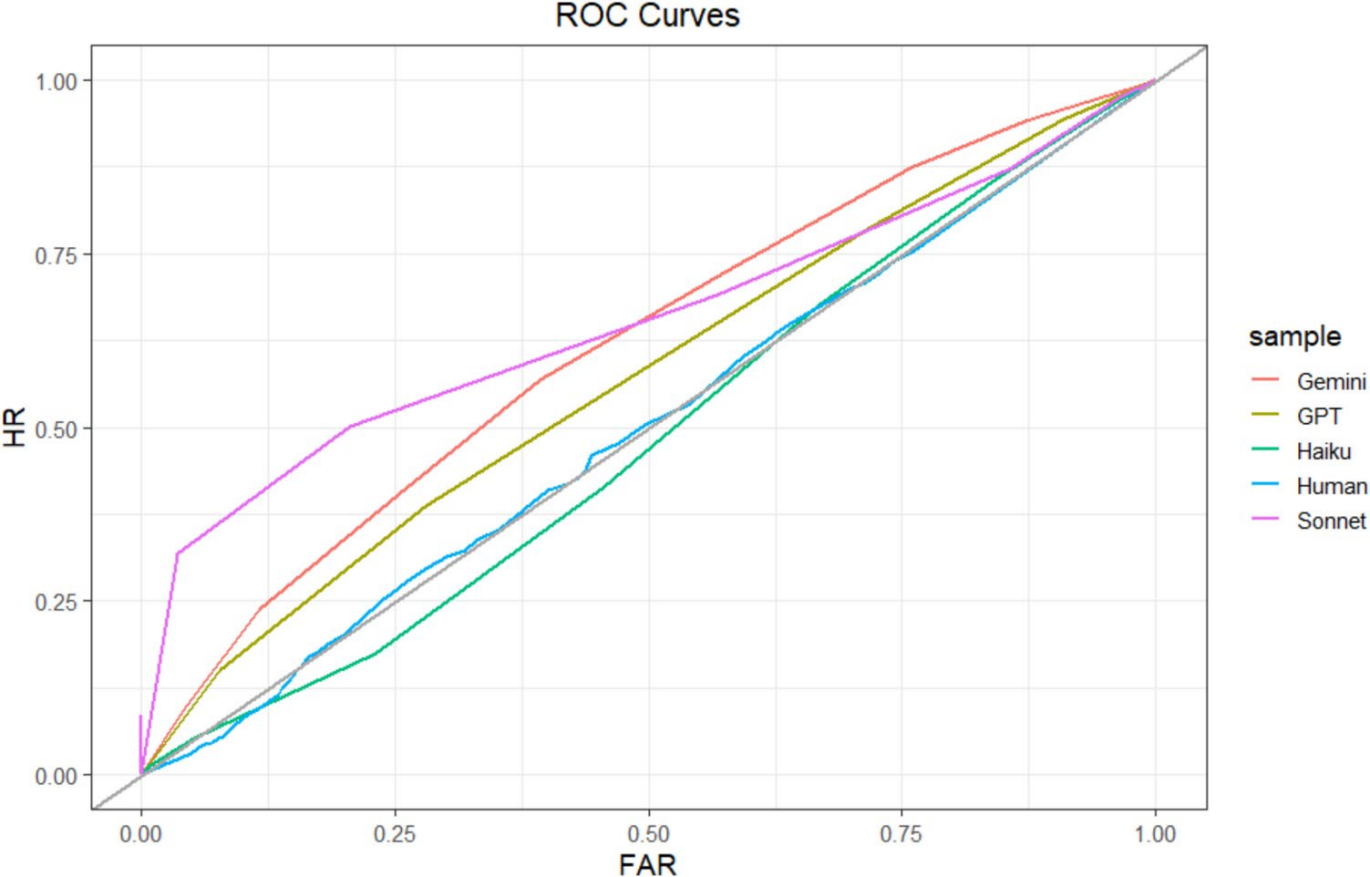
Study 4 ROC curves. Type 2 ROC curve for each sample. The gray identity line indicates chance. HR = hit rate; FAR = false-alarm rate. (Color figure online)

We assessed relative metacognitive accuracy using the same methods as in the previous studies. At the sample level, humans (110 participants; 2,420 guesses) achieved an AUROC of 0.50, 95% CI [0.48, 0.52]. ChatGPT, AUROC = 0.57, 95% CI [0.55, 0.60]; Gemini, AUROC = 0.62, 95% CI [0.59, 0.64]; and Sonnet, AUROC = 0.65, 95% CI [0.63, 0.68] achieved greater AUROCs than humans (*D* values > 4.23, *df* values > 4576.7; *p *values <.001). Haiku’s AUROC (AUROC = 0.48, 95% CI [0.46, 0.51]) was not significantly different than that of humans (*D* =0.93, *df* = 4558, *p* =.35). Sample-level ROCs are depicted in Fig. 9. These results indicate that humans and Haiku had very poor relative metacognitive accuracy, while the other LLMs were slightly better, though still far from excellent.

When assessed at the participant level, the average human AUROC (*M* = 0.55, *sd* = 0.10) was lower than the average AUROC for ChatGPT (*M* = 0.60, *sd* = 0.10), *t*(204.03) = −3.52, *p* =.001; Gemini (*M* = 0.62, *sd* = 0.08), *t*(206.95) = −5.91, *p* <.001; and Sonnet (*M* = 0.66, *sd* = 0.10), *t*(205.54) = −8.07, *p* <.001, but not significantly different than the average AUROC for Haiku (*M* = 0.57, *sd* = 0.09), *t*(207.94) = −1.27, *p* =.20. These results mirror the sample-level results, again suggesting that humans and Haiku had the worst relative metacognitive accuracy.

#### Item-level comparisons

Lastly, we conducted exploratory analyses in which we compared mean accuracy, confidence, and overconfidence for humans and LLMs at the item level (i.e., each question). Humans’ question-level accuracy was not significantly correlated with the question-level accuracy of any of the LLMs (*r* values = .05-.21, *p* values ≥.35). Question-level accuracy was marginally or significantly correlated for each pair of LLMs (*r* values =.41–.82, *p* values ≤.06), except Haiku and Gemini (*r* =.13, *p* =.56). Humans’ question-level confidence was marginally correlated with Gemini’s question-level confidence (*r* =.37, *p* =.09) and significantly correlated with Sonnet’s (*r* =.46, *p* =.03), but not significantly correlated with ChatGPT’s (*r* =.17, *p* =.45) or Haiku’s (*r* =.19, *p* =.39). Question-level confidence was significantly correlated for each pair of LLMs (*r* values =.45–.64, *p* values <.04) except Gemini and Sonnet (*r* =.29, *p* =.19). Humans’ question-level overconfidence was not significantly correlated with the question-level overconfidence of any of the LLMs (*r *values =.01–.11, *p* values >.63). Question-level overconfidence was marginally or significantly correlated for each pair of LLMs (*r* values =.41–.83, *p* values <.06), except Gemini and Haiku (*r* =.11, *p* =.62). In contrast to Study 3, these results indicate that humans and LLMs found different items difficult, were more confident on different items, and were well-calibrated (or poorly calibrated) on different items, suggesting cognitive and metacognitive differences.

### Study 4 discussion

Study 4 provided evidence of how humans and LLMs respond to a trivia task that is more akin to the types of questions that people may ask LLMs to answer. We found that humans were well-calibrated prospectively but became underconfident after completing the task. In fact, human participants became so underconfident that they predicted that they would perform worse than chance. The LLMs, on the other hand, were largely overconfident, both prospectively and retrospectively. The one exception was Sonnet, which was underconfident both prospectively and retrospectively. In terms of calibration errors, the two Claude models outperformed humans, ChatGPT performed as well as humans, and Gemini underperformed humans. Interestingly, the Claude models demonstrated improved calibration after completing the task, while none of the other samples (including humans) improved. Gemini became less well-calibrated after completing the task. In terms of relative metacognitive accuracy, humans performed worse than Gemini, ChatGPT, and Sonnet. These LLMs did not have particularly good relative metacognitive accuracy, but humans’ relative metacognitive accuracy was so low that it suggested that their item-level confidence judgments were essentially random. In all, these results suggest that in trivia tasks, humans largely have worse absolute and relative metacognitive accuracy than LLMs. Study 5 seeks to generalize these results to another trivia-based task, this time using questions derived from datasets that were not available on the internet.

## Study 5

### Methods

Study 5 was preregistered on AsPredicted. The preregistration adheres to the disclosure requirements of AsPredicted. All analyses were preregistered. The preregistration can be found online (https://aspredicted.org/yd7f-rjd5.pdf).

#### Participants

Following the same reasoning as Study 4, we recruited 110 American Prolific participants to complete this study. All 110 participants completed the study, and no participants were excluded. Demographics are reported in the Supplemental Materials.

#### Stimuli

The research team created a novel set of 33 trivia questions about life at a “mid-sized private university in the Midwest.” All questions were formatted such that the participant had to guess whether a certain value was higher or lower than another value. These questions were divided into three topic-based categories. In the *Sports GPA* category, questions asked about the average GPA of different sports teams at the university (e.g., “Did the women’s basketball team have a higher or lower GPA than the men’s basketball team?”). In the *Course Rating* category, questions asked about the average ratings that students gave to various sets of courses (e.g., “Did courses in the engineering school receive higher or lower average ratings than courses in the fine arts school?”). Finally, in the *Bookstore Prices* round, questions asked whether the most expensive item in a category cost more or less than a given value (e.g., “Is the price of the most expensive jacket in the university’s bookstore higher or lower than $125?”). Participants were told that all data was collected from the same university but were not told which university it was.

This unique question set was specifically designed such that the questions were impossible to look up, which required the underlying data not to be publicly available online. *Sports GPA* and *Course Rating* data were provided to the research team by official university sources and *Bookstore Price* data were collected manually by an undergraduate research assistant. We further reduced the risk of participants looking up the data by not disclosing the identity of the university. The order of the categories was randomized. The full set of questions—along with question-level mean accuracy and confidence—is provided in the Supplemental Materials.

#### Procedure

Participants played a game in which they answered the 33 trivia questions described above. Participants first completed a practice round in which they answered one example question from each category, with feedback about the correct answer. They then completed the three main rounds, one for each category. Each main round contained 10 questions. Participants earned a $0.01 bonus for each correct guess in the main rounds.

For each question, participants were asked to rate how likely they thought it was that their guess was correct, on a scale of 50% (“Random Guess”) to 100% (“Certain”). After the practice round, participants made three *prospective* overall confidence judgments in which they predicted how many of the 10 questions they thought they would get right in each main round. After each main round, participants made a *retrospective* overall confidence judgment estimating how many of the 10 questions they thought they had correctly guessed in that round. Each overall confidence judgment was incentivized with a $0.05 accuracy bonus.

ChatGPT-4o, Gemini Flash 2.0, Claude Sonnet 3.7, and Claude Haiku 3.5 were each asked to complete the same tasks as the human participants 100 times. The script used to generate responses from the LLMs is provided in the Supplemental Materials. All data was collected between March 1–March 25, 2025.

### Results

We evaluated performance only in the main rounds. For our primary analyses we aggregated data across categories. Data for each category are reported in the Supplemental Materials.

#### Absolute metacognitive accuracy

On average, human participants correctly answered 15.45 questions (*sd* = 2.49). Prospectively, they predicted that they would correctly answer 17.96 questions (*sd* = 4.81). Retrospectively, the average estimate was 16.72 questions (*sd* = 4.32). Paired *t* tests indicated that humans were overconfident both prospectively, *t*(109) = 4.71, *p <.*001, *d* = 0.45, and retrospectively, *t*(109) = 2.47, *p =.*02, *d* =.24. Humans’ prospective average calibration error was 4.84 questions (*sd* = 3.74), and their retrospective average calibration error was 4.28 (*sd* = 3.45). Their retrospective calibration was marginally better than their prospective calibration, *t*(109) = −1.80, *p* =.07.

ChatGPT correctly answered 16.30 questions on average (*sd* = 1.28), which was significantly higher than the human participants, *t*(166.39) = 3.13, *p* =.002. Prospectively, ChatGPT predicted that it would correctly answer an average of 22.69 questions (*sd* = 1.28). Retrospectively, ChatGPT estimated that it correctly answered an average of 22.92 questions (*sd* = 1.14). Paired *t* tests indicated that ChatGPT was overconfident both prospectively, *t*(99) = 32.80, *p* <.001, *d* = 3.28, and retrospectively, *t*(99) = 35.85, *p <.*001, *d* = 3.59. ChatGPT’s prospective average calibration error was 6.39 questions (*sd* = 1.95), which was significantly lower than its retrospective average calibration error of 6.62 questions (*sd* = 1.85), *t*(99) = −4.06, *p* <.001. ChatGPT’s average calibration error was significantly higher that of humans both prospectively, *t*(162.42) = 3.83, *p* <.001, and retrospectively, *t*(169.83) = 6.19, *p* <.001, suggesting worse absolute accuracy.

Gemini correctly answered 16.68 questions on average (*sd* = 1.50), which was significantly better than the average accuracy of the human participants, *t*(181.36) = 4.37, *p* <.001. Prospectively, Gemini predicted that it would correctly answer an average of 17.75 questions (*sd* = 1.09). Retrospectively, Gemini estimated that it correctly answered an average of 20.24 questions (*sd* = 1.12). Paired *t* tests indicated that Gemini was overconfident both prospectively, *t*(99) = 6.00, *p <.*001, *d* = 0.60, and retrospectively, *t*(99) = 20.10, *p <.*001, *d* = 2.01. Gemini’s prospective average calibration error was 1.57 questions (*sd* = 1.73), which was significantly lower than its retrospective average calibration error of 3.58 questions (*sd* = 1.73), *t*(99) = −4.06, *p* <.001. Gemini’s average calibration error was significantly lower than that of humans prospectively, *t*(139.69) = −8.56, *p* <.001, and marginally lower retrospectively, *t*(163.74) = −1.89, *p* =.06, indicating better absolute accuracy.

Sonnet correctly answered 19.28 questions on average (*sd* = 1.16), which was significantly better than the average accuracy of the human participants, *t*(157.71) = 14.47, *p* <.001. Prospectively, Sonnet predicted that it would correctly answer an average of 18.94 questions (*sd* = 0.87). Retrospectively, Sonnet estimated that it correctly answered an average of 20.26 questions (*sd* = 1.28). Paired *t* tests indicated that Sonnet was underconfident prospectively, *t*(99) = −2.45, *p =.*02, *d* = −0.25, but overconfident retrospectively, *t*(99) = 5.71, *p <.*001, *d* = 0.57. Sonnet’s prospective average calibration error was 1.08 questions (*sd* = 0.93), which was significantly lower than its retrospective average calibration error of 1.50 questions (*sd* = 1.28), *t*(99) = −3.15, *p* =.002. Sonnet’s average calibration error was significantly lower than that of humans both prospectively, *t*(123.67) = −10.20, *p* <.001, and retrospectively, *t*(141.03) = −7.87, *p* <.001, indicating better absolute accuracy

**Fig. 10:**
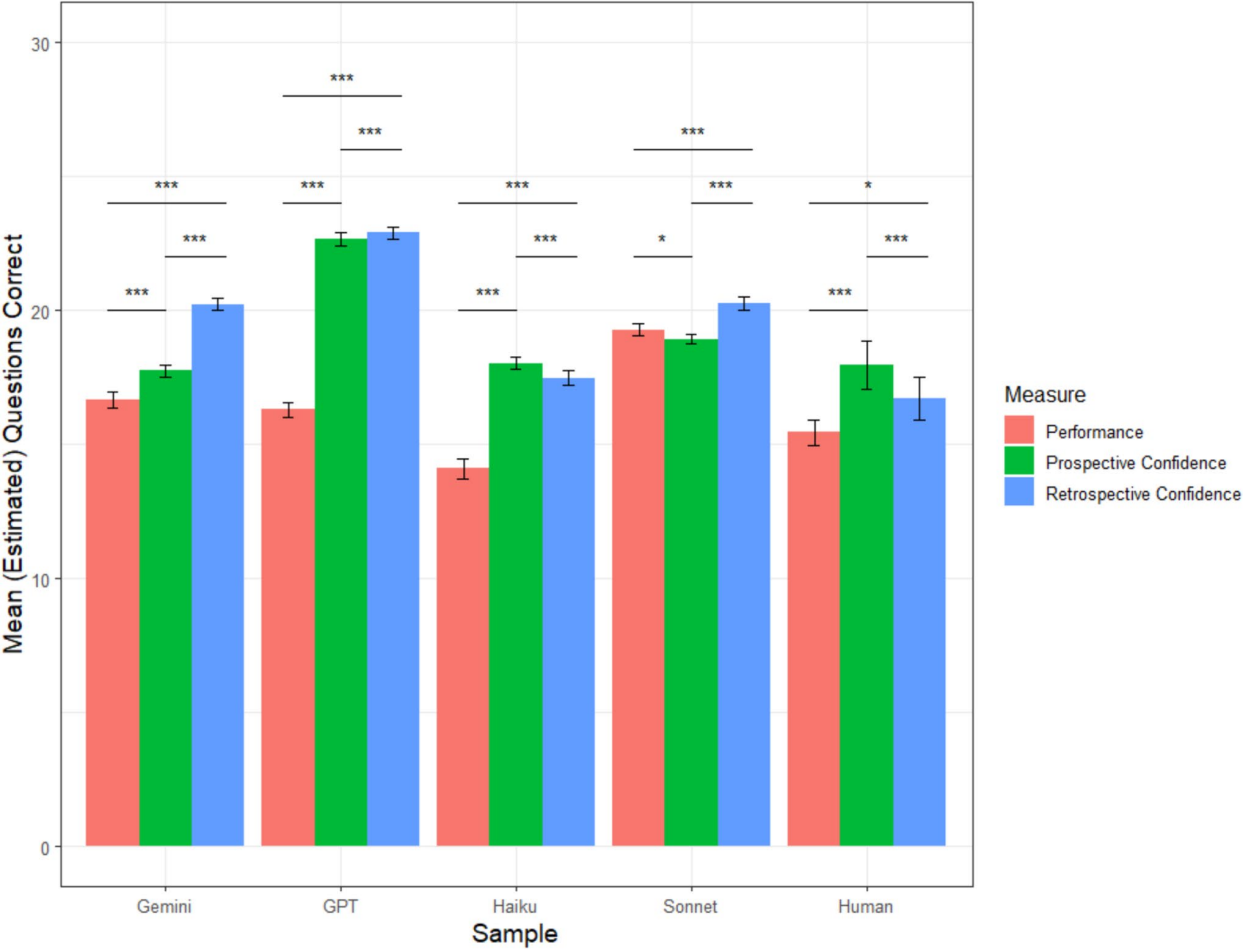
Study 5 mean accuracy and confidence by sample. *Note. *Error bars reflect 95% confidence intervals; ^†^*p* <.10; *p <.05, **p <.01, ***p <.001. (Color figure online)

Haiku correctly answered 14.10 questions on average (*sd* = 1.89), which was significantly worse than the average accuracy of the human participants, *t*(201.70) = −4.47, *p* <.001. Prospectively, Haiku predicted that it would correctly answer an average of 18.04 questions (*sd* = 1.09). Retrospectively, Haiku estimated that it correctly answered an average of 17.48 questions (*sd* = 1.42). Paired *t* tests indicated that Haiku was overconfident both prospectively, *t*(99) = 19.09, *p <.*001, *d* = 1.91, and retrospectively, *t*(99) = 13.99, *p <.*001, *d* = 1.40. Haiku’s prospective average calibration error was 3.98 (*sd* = 1.98), which was significantly higher than its retrospective average calibration error of 3.64 (*sd* = 2.00), *t*(99) = 2.81, *p* =.006. Haiku’s average calibration error was significantly lower than that of humans prospectively, *t*(169.17) = −2.10, *p* =.04, and marginally lower retrospectively, *t*(177.51) = −1.67, *p* =.097, suggesting better absolute accuracy. A graphical representation of each sample’s mean accuracy, prospective confidence, and retrospective confidence is depicted in Fig. 10.

#### Relative metacognitive accuracy

**Fig. 11:**
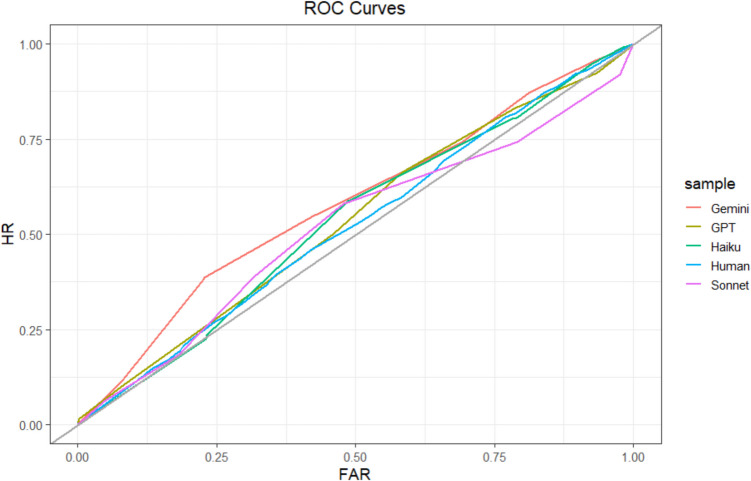
Study 5 ROC curves*. Note. *Type 2 ROC curve for each sample. The gray identity line indicates chance. HR = hit rate. FAR = false-alarm rate. (Color figure online)

We assessed relative metacognitive accuracy using the same methods as in the previous studies. At the sample level, humans (110 participants; 3,300 guesses) achieved an AUROC of 0.52, 95% CI [0.50, 0.54]. Gemini, (AUROC = 0.58; 95% CI [0.56, 0.60]) achieved a greater AUROC than humans (*D* = 3.97, *df* = 6267.4, *p* values <.001). ChatGPT (AUROC = 0.54; 95% CI [0.51, 0.56]), Sonnet (AUROC = 0.52; 95% CI [0.50, 0.54]), and Haiku (AUROC = 0.54; 95% CI [.52,.56]) achieved AUROCs that were not significantly different than humans (*D* values = −0.28–0.90, *df* values > 6225.90, *p* values >.37). Sample-level ROCs are depicted in Fig. 11. These results indicate that humans and LLMs had rather poor relative metacognitive accuracy, though Gemini was slightly better than humans.

When assessed at the participant level, the average human AUROC (*M* = 0.57, *sd* = 0.08) was marginally higher than the average AUROC for ChatGPT (*M* = 0.55, *sd* = 0.06), *t*(196.42) = 1.95, *p* =.05, and significantly higher than the average AUROC for Sonnet (*M* = 0.53, *sd* = 0.06), *t*(196.24) = 3.75, *p* <.001, and Haiku (*M* = 0.53, *sd* = 0.08), *t*(202.39) = 3.29, *p* =.001. The average human AUROC was not significantly different than the average AUROC for Gemini (*M* = 0.57, *sd* = 0.08), *t*(203.74) = 0.37, *p* =.71. These results paint a slightly better picture of humans’ relative metacognitive accuracy—putting them on par with Gemini and above the other LLMs—but average AUROCs were still quite low for each sample, including humans.

#### Item-level comparisons

Finally, we conducted exploratory analyses in which we compared mean accuracy, confidence, and overconfidence for humans and LLMs at the item level (i.e., each question). Humans’ question-level accuracy was significantly correlated with the question-level accuracy of each LLM (*r* values =.50–.71, *p* values ≤.005). Question-level accuracy was significantly correlated for each pair of LLMs as well (*r* values =.45–.69, *p* values ≤.01). Humans’ question-level confidence was significantly correlated with the question-level confidence of each LLM (*r* values =.46–.71, *p* values ≤.01). Question-level confidence was marginally or significantly correlated for each pair of LLMs (*r* values =.35–.83, *p* values <.06) except Haiku and ChatGPT (*r* =.26, *p* =.16). Lastly, humans’ question-level overconfidence was significantly correlated with the question-level overconfidence of each LLM (*r* values =.51–.67, *p* values ≤.004). Question-level overconfidence was significantly correlated for each pair of LLMs (*r* values =.42–.67, *p* values <.02). Like Study 3, and in contrast to Study 4, these results suggest that humans and LLMs found the same items difficult, were more confident on the same items, and were well-calibrated (or poorly calibrated) on the same items, suggesting some cognitive and metacognitive similarities.

### Study 5 discussion

Study 5 provided evidence of how humans and LLMs respond to fact-based questions that cannot be looked up by using datasets that were not publicly available. When evaluating absolute metacognitive accuracy, we found that all samples except Sonnet were overconfident, both prospectively and retrospectively. Sonnet was underconfident prospectively, but overconfident retrospectively. All models except ChatGPT demonstrated greater absolute metacognitive accuracy than humans. Haiku and humans demonstrated greater calibration retrospectively than prospectively, while the other samples demonstrated worse calibration retrospectively—thus replicating our result from Study 3 that LLMs often fail to learn from their experiences. When relative metacognitive accuracy was assessed at the sample level, Gemini was slightly more accurate than humans, while all other models were equally as accurate as humans. When relative metacognitive accuracy was assessed at the participant level, humans were more accurate than all models except Gemini. However, all samples’ relative metacognitive accuracy would be considered poor or failing, given how close their performance was to chance (Nahm, 2022).

## General discussion

Across five different domains, we evaluated the ability of LLMs—including ChatGPT, Bard/Gemini, Claude Haiku, and Claude Sonnet—to provide aggregate and item-level confidence judgments. We then compared their confidence judgments with their performance to assess their absolute and relative metacognitive accuracy (Fleming & Lau, 2014; Higham & Higham, 2019; Maniscalco & Lau, 2012). The most obvious conclusion is that the accuracy of LLMs’ confidence judgments varied by domain and by model; there was no overwhelming pattern that would lead us to conclude that LLMs were more or less accurate than humans across the board. However, there were some interesting trends in the data.

### Absolute metacognitive accuracy

When evaluating participants’ absolute metacognitive accuracy—measured via calibration errors—we found mixed results across models and tasks. Both Claude models had consistently better absolute accuracy than humans, while ChatGPT and Bard/Gemini were more variable in their performance relative to humans. In Table 1, we summarize whether each LLM achieved better, equal, or worse absolute accuracy than humans in each study. Out of 24 comparisons, LLMs were more accurate than humans 13 times, equally as accurate as humans four times, and less accurate than humans seven times. In general, these results suggest that LLMs may have slightly greater absolute accuracy than humans, but that the differences are highly variable by domain and model.

### Overconfidence

Another pattern we found is that both LLMs and humans were largely overconfident. When making prospective confidence judgments (Studies 3–5 only), ChatGPT, Gemini, and Haiku were overconfident in every instance. In contrast, Sonnet was prospectively underconfident in both studies in which it appeared. Humans were prospectively overconfident in Studies 3 and 5, and well-calibrated in Study 4. Overall, this suggests that LLMs, like humans (Moore & Healy, 2008; Moore & Schatz, 2017), are generally biased towards overconfidence, rather than underconfidence—with Sonnet being a notable exception.

When making retrospective confidence judgments, ChatGPT was overconfident in all studies except Study 1 and Study 2 (in which we were too underpowered to make claims), Bard/Gemini was overconfident in all studies except Study 2, and Haiku was overconfident in both studies in which it appeared (Studies 4 and 5). Sonnet was underconfident in Study 4 and overconfident in Study 5. In contrast, humans were a mixed bag—they were retrospectively overconfident in Studies 2, 3, and 5, but retrospectively underconfident in Studies 1 and 4. This suggests that LLMs may be more consistently overconfident than humans when making retrospective confidence judgments.

### Developing metacognitive insight from experience

We were also interested in exploring the extent to which humans and LLMs improved their absolute metacognitive accuracy after completing each task, which would indicate that they were learning from their experience and updating their metacognitive judgments accordingly. This would be reflected in retrospective calibration errors that were lower than prospective calibration errors. Humans followed this pattern in Study 3 and Study 5. In Study 4, humans’ retrospective errors were not significantly different in magnitude than their prospective errors. In contrast, we found that ChatGPT and Gemini tended to have larger retrospective errors than prospective errors—indicating not only that they were failing to learn from their performance but that they were actively becoming less well-calibrated. Sonnet achieved a lower retrospective error than prospective error in Study 4 but did the opposite in Study 5. Haiku was the only LLM that consistently achieved retrospective errors that were lower than prospective errors—doing so in both Study 4 and Study 5.

These results demonstrate a contrast between humans and some LLMs. Humans generally improved their metacognitive accuracy after completing a task—and at minimum, never became significantly less accurate. This aligns with the prior metacognition literature, which suggests that humans improve their metacognitive calibration through experience (Fleming et al., 2016; Siedlecka et al., 2016). In contrast, the LLMs demonstrated more random performance, such that their retrospective errors were sometimes better than their prospective errors and sometimes worse. This insensitivity to performance (i.e., lack of learning) suggests that LLMs do not have the capacity to learn from their own experience—supporting the hypothesis that LLMs do not have access to mnemonic metacognitive cues (Cash & Oppenheimer, 2024), although Haiku merits further study as it was out of alignment with other LLMs on this dimension.

### Relative metacognitive accuracy

In terms of relative metacognitive accuracy, we found that accuracy was quite low for all samples in Study 1, Study 4, and Study 5. In contrast, relative metacognitive accuracy was quite high for all samples in Study 3. Relative metacognitive accuracy in Study 2 was more variable across samples, with ChatGPT performing well while humans and Gemini performed poorly.

When comparing across samples, we found that LLMs generally demonstrated equal or better relative accuracy than humans. This trend was particularly consistent when relative accuracy was assessed at the sample level. In Table 2, we summarize whether each LLM achieved better, equal, or worse relative accuracy—measured via AUROCs—than humans in each study. Sample-level and participant-level results are reported separately. Out of 28 total comparisons, LLMs were more accurate 10 times, equally accurate 14 times, and less accurate four times.

Another interesting—albeit highly speculative—pattern is that, in terms of relative metacognitive accuracy, LLMs always performed as well or better than humans in the studies in which uncertainty was primarily aleatory (Studies 1 and 2), but achieved more mixed results in the studies in which uncertainty was primarily epistemic (Studies 3–5; Fox & Ülkümen, 2011). This effect was particularly notable for the participant-level AUROCs, which are the most typical measure of relative metacognitive accuracy (Higham & Higham, 2019). This may suggest that humans have a comparative advantage on tasks in which uncertainty is epistemic—likely as a result of their access to mnemonic cues (Cash & Oppenheimer, 2024)—but that this advantage disappears when uncertainty is aleatory. However, this finding was highly confounded by time, given that the epistemic uncertainty studies were conducted later than the aleatory uncertainty studies. As such, we encourage future research into how LLMs respond to different forms of uncertainty.

### Item-level comparisons

In exploratory analyses, we investigated whether humans and LLMs were similarly accurate, confident, and overconfident on the same items. In Studies 3 and 5, we found significant correlations between humans and LLMs for all three item-level measures, indicating some cognitive and metacognitive similarities. However, in Study 4 we found small and inconsistent correlations between humans and LLMs. Correlations between pairs of LLMs were high and generally significant, with only a few exceptions. This pattern of results suggests that LLMs generally engage in similar cognitive and metacognitive processes, whereas the similarity between humans and LLMs is domain dependent. Further research is needed to make substantial claims about why certain domains promote cognitive and metacognitive similarity between humans and LLMs, while others induce divergence.

## Summary

In conjunction, these results provide interesting insights into the accuracy of confidence judgments made by LLMs. First, we find that LLMs do not consistently show better or worse absolute metacognitive accuracy than humans, though they perform slightly better on average. The two Claude models tended to outperform humans, while ChatGPT and Gemini had more variable performance. Second, we show that LLMs, like humans (Moore & Healy, 2008; Moore & Schatz, 2017), tend to be overconfident. Third, we demonstrate that—unlike humans— LLMs largely fail to learn from their experiences and often provide retrospective confidence judgments that are *less* accurate than their prospective confidence judgments (although Haiku diverges from this pattern), perhaps reflecting a lack of metacognitive insight (Ackerman & Thompson, 2017; Alter & Oppenheimer, 2009; Cash & Oppenheimer, 2024; Thompson et al., 2013). Finally, we find that humans and LLMs achieve similar levels of relative metacognitive accuracy, with LLMs having perhaps a slight advantage over humans. This was true in both tasks where relative metacognitive accuracy was high on average and tasks where it was low on average. In all, LLMs’ confidence judgments are not uniformly better or worse than humans’ confidence judgments but tend to be slightly more accurate.

However, it is critical to acknowledge that similar outcomes do not necessarily imply similar processes. LLMs and humans likely generate confidence judgments using different statistical and metacognitive processes. For example, humans have access to experiential (i.e., mnemonic) metacognitive cues that can inform their confidence judgments (Ackerman & Thompson, 2017; Koriat, 1997; Thompson et al., 2013). Indeed, this interpretation is reinforced by the differences between prospective and retrospective calibration. After experiencing the task, humans became less confident and typically better calibrated, while LLMs did not consistently show that pattern. In contrast, LLMs have a much larger dataset to inform their judgments and can process far more statistical information than a human participant (Wu et al., 2023). These different approaches may suggest that humans are using true metacognitive processes, whereas LLMs may be merely parroting the confidence judgments made by humans in their training set (Cash & Oppenheimer, 2024; Roose, 2023; Wolfram, 2023). Furthermore, we found that LLMs and humans were not consistently well-calibrated on the same items. In Study 4, item-level overconfidence values were not significantly correlated between humans and LLMs, suggesting dissimilar metacognitive processes. In Studies 3 and 5, item-level overconfidence values were significantly correlated for humans and LLMs, but most of the correlations were not high enough to suggest identical processes (e.g., only one correlation surpassed the gold standard of *r* = 0.7 for test–retest reliability). These exploratory findings suggest, at minimum, that humans and LLMs do not use identical metacognitive processes. Future research should delve more deeply into the mechanisms underlying linguistic confidence judgments provided by LLMs and compare them to the mechanisms underlying humans’ confidence judgments.

## Limitations

The studies presented here were exploratory in nature and reflect only a small fraction of the domains in which LLMs may be asked to make confidence judgments. Future research should seek to generalize our findings in other domains and to explore the conditions under which LLMs’ confidence judgments may more drastically diverge from those made by humans. For example, it is unclear whether LLMs’ confidence judgments would be as accurate as humans’ in low-information environments, when LLMs lose their information-processing advantage (Murayama et al., 2016). Another open question is whether LLMs may experience a boost (or decline) in metacognitive accuracy if prompted to respond using a certain persona (Schoenegger et al., 2024; Shanahan et al., 2023).

Another limitation of the studies presented here is that they only assess four of the many LLMs that have become available to users. Future research should evaluate additional models. However, it is worth noting that the data presented here was collected over the course of a year and a half, using different generations of each LLM. This suggests that our findings were unlikely to be an artifact of the models selected and that technological advances seemingly have not improved the metacognitive capacities of the LLMs studied here. Despite this, it is of empirical interest to continue benchmarking the metacognitive capacities of future generations of LLMs. From a research perspective, it may be particularly valuable to explore open-source models (e.g., LLaMa, Mixtral) as their nonproprietary nature allows for greater replicability.

Another limitation of our design—and a challenge of LLM research more broadly—is that individual conversations with a generative AI chatbot are unlikely to be purely independent observations in the way that different human participants are. When you ask an LLM the same question repeatedly, it tends to give similar answers, which may result in biased statistical analyses. This concern could be mitigated by increasing the temperature hyperparameter on the model—which increases the variance in the model’s responses (Renze, 2024). However, we chose to use the default hyperparameters (including temperature) for each model, since that is how users are most likely to experience the LLMs. It would be of great empirical interest to evaluate how changing hyperparameters like temperature influence the accuracy of LLMs’ confidence judgments.

A final limitation of our design is that we compared average humans with the best LLMs. Just like there are individual differences in humans’ metacognitive abilities (Ackerman & Levontin, 2024; West & Stanovich, 1997), there are likely to be individual differences in the metacognitive abilities of LLMs (indeed, we observed some—with Haiku outperforming other models). However, only the best LLMs make it to market—thus making the poor-performing LLMs inaccessible to researchers. It would be interesting to compare the metacognitive abilities of the most metacognitively capable humans—such as domain experts (Cash & Oppenheimer, 2024; Han & Dunning, 2024)—with the LLMs. This may be a fairer comparison, since it would be comparing the best humans to the best LLMs.

## Conclusion

The studies presented here provided evidence that LLMs’ confidence judgments are about as accurate as those provided by humans. We do find some evidence that LLMs—particularly those produced by Claude—are perhaps slightly more accurate than humans when estimating their overall performance. Similarly, LLMs may be slightly more accurate than humans when estimating item-level confidence. Interestingly, however, we find that LLMs are not consistently capable of updating their metacognitive judgments based on their experiences. We also find that, like humans, LLMs tend to be overconfident. We believe that these conclusions can help human users better understand the extent to which they should trust LLMs’ confidence judgments and hope that these results spark new interest in studying the metacognitive capacities of LLMs. When we asked the LLMs whether this research would inspire future research on LLM metacognition, they were highly confident that it would (see Supplemental Materials).

**Table 1:** LLM versus human calibration errors

	Study 1	Study 2	Study 3	Study 4	Study 5
*Prospective calibration error*					
ChatGPT	–	**–**	Better	Equal	Worse
Bard/Gemini	**–**	**–**	Worse	Worse	Better
Sonnet	**–**	**–**	**–**	Better	Better
Haiku	**–**	**–**	**–**	Better	Better
*Retrospective calibration error*					
ChatGPT	Better	Equal	Worse	Equal	Worse
Bard/Gemini	Equal	Better	Worse	Worse	Better^†^
Sonnet	**–**	**–**	**–**	Better	Better
Haiku	**–**	**–**	**–**	Better	Better^†^

^**†**^Marginally significant

**Table 2:** LLM versus human relative accuracy

	Study 1	Study 2	Study 3	Study 4	Study 5
*Sample-level AUROCs*					
ChatGPT	Equal	Better	Equal	Better	Equal
Bard/Gemini	Better^†^	Equal	Equal	Better	Better
Sonnet	–	–	–	Better	Equal
Haiku	–	–	–	Equal	Equal
*Participant-level AUROCs*					
ChatGPT	Equal	Better	Worse	Better	Worse^†^
Bard/Gemini	Equal	Equal	Equal	Better	Equal
Sonnet	–	–	–	Better	Worse
Haiku	–	–	–	Equal	Worse

^**†**^Marginally significant.

## Supplementary Information

Below is the link to the electronic supplementary material.

Supplementary file1 (PDF 1525 KB)

## Data Availability

The code for all studies is available at: https://osf.io/b6qhx/?view_only=219cc3ad034542f6bd4271457f87ef1f

## Declarations

### Open practices statement

The data and materials for all studies are available [https://osf.io/b6qhx/?view_only=219cc3ad034542f6bd4271457f87ef1f]. Study 2 [https://aspredicted.org/T1L_2J4], Study 3 [https://aspredicted.org/BJP_BZX], Study 4 [https://aspredicted.org/f33r-z39k.pdf], and Study 5 [https://aspredicted.org/yd7f-rjd5.pdf] were preregistered on AsPredicted.
