# Supplementary material for: Quantifying uncert-AI-nty: Testing the accuracy of LLMs’ confidence judgments
Source: Mem Cognit. 2025 Jul 22;54(2):375–400. doi: 10.3758/s13421-025-01755-4 (PMC12957136; doi:10.3758/s13421-025-01755-4)
Supplement: Supplementary file 1 — Supplementary file1 (PDF 1525 KB) [file 13421_2025_1755_MOESM1_ESM.pdf]

Supplemental Materials - Table of Contents

Pre-Registration Deviation Table	3-5
**Study 1 Supplemental Materials**	**6-18**
Demographics	7
Demographics predicting Individual-Level Overconfidence	8
Sample Prediction Task	9
LLM Script + Sample Responses	10-11
Week-by-Week (Predicted) Accuracy Data	12
Distribution of Individual-Level Overconfidence, by Sample	13
Absolute Metacognitive Accuracy using Total (Estimated) Accuracy	14
Robustness Check Excluding Week 18	15-17
Comparing ChatGPT & Bard	18
**Study 2 Supplemental Materials**	**19-36**
Demographics	20
Demographics predicting Individual-Level Overconfidence	21
Sample Prediction Task	22
LLM Script + Sample Responses	23-34
Distribution of Individual-Level Overconfidence, by Sample	35
Comparing ChatGPT & Gemini	36
**Study 3 Supplemental materials**	**37-52**
Demographics	38
Demographics Predicting Prospective Overconfidence	39
Demographics Predicting Retrospective Overconfidence	40
Sample Pictionary Task	41
Mean Accuracy and Confidence By Word, By Sample	42-43
LLM Script + Sample Responses	44-46
Coding Guide	47
Item-Level Difficulty & Overconfidence	48
Distribution of Individual-Level Overconfidence, by Sample	49-50
Comparing ChatGPT & Gemini	51

Accuracy and Confidence by Artist	52
**Study 4 Supplemental Materials**	**53-73**
Demographics	54
Demographics Predicting Prospective Overconfidence	55
Demographics Predicting Retrospective Overconfidence	56
Sample Trivia Question	57
Mean Accuracy and Confidence By Question, By Sample	58-61
LLM Script + Sample Responses	62-68
Item-Level Difficulty & Overconfidence	69
Distribution of Individual-Level Overconfidence, by Sample	70-71
Comparing LLMs to one another	72-73
**Study 5 Supplemental Materials**	**74-106**
Demographics	75
Demographics Predicting Prospective Overconfidence	76
Demographics Predicting Retrospective Overconfidence	77
Sample Question	78
Mean Accuracy and Confidence By Question, By Sample	79-84
LLM Script + Sample Responses	85-99
Item-Level Difficulty & Overconfidence	100
Distribution of Individual-Level Overconfidence, by Sample	101-102
Comparing LLMs to one another	103-104
Absolute Metacognitive Accuracy by Question Category	105
Relative Metacognitive Accuracy by Question Category	106
**General Supplemental Materials**	**107-113**
LLM Confidence in the Impact of this Paper	108-113

Preregistration Deviations Table

(adapted from Willroth & Atherton, 2023)

Table S1

Preregistration deviations

#	Details		Original wording	Deviation description	To what extent is this a deviation from the preregistered plan?	Judgment of impact
1	Type	Analyses	Study 2: “*For the human participants and the LLMs, we will calculate the gamma correlation between confidence (20% – 100%) and accuracy (binary). We will then compare the gamma correlations for the human participants and the LLMs*.”<br><br>Study 3: *“For the human participants and the LLMs, we will calculate the gamma correlation between confidence (0% – 100%) and accuracy (binary). We will then compare the gamma*	After discussion with reviewers, we chose to run an SDT-style analysis (ROC curves) rather than gamma correlations. We feel that this is a less biased analysis that will result in more valid results<br><br>In the Study 2 pre-registration, we stated that we would do both, but in the Study 3 pre-registration we only pre-registered the gamma analyses.<br><br>We corrected this matter in Studies 4 and 5.	Major	The primary impact of this deviation is that we will use a different analysis than we originally pre-registered. While this new analysis may come to a different conclusion, we believe that the conclusion will be more valid – and thus is the more appropriate analysis to report.
	Reason	Peer review				
	Timing	After data access				

			correlations for the human participants and the LLMs."			
2	Type	Methods	Study 2: "*We will recruit 100 human participants*."	Our goal was to have viable data from 100 respondents, so we recruited 110 to account for low-quality data. Our pre-registration was unclear and suggested we would recruit only 100.	Minor	We do not believe this significantly impacted our results, but it is possible that if we had recruited only 100 participants our results may have been weaker.
	Reason	Miscommu nication				
	Timing	Before data collection				
3	Type	Analyses	Study 2 (Example): "*We will use t-tests to determine whether the accuracy of the LLMs was significantly different than the accuracy of the human participants."*<br><br>Study 3 (Example): "*We will use one-way ANOVAs to determine whether the predicted accuracy of the LLMs was significantly different than the predicted accuracy of the human participants*."	Our pre-registrations for Study 2 and Study 3 only mentioned that we would compare the LLMs to Humans – and failed to acknowledge that we would also make comparisons between the two LLMs. While these analyses were more exploratory in nature, they still should have been pre-registered.<br><br>We included this as an exploratory analysis in the pre-registrations for	Minor	The primary impact of this omission was that we ran additional analyses that were not pre-registered. To avoid bias, these should be considered as exploratory analyses. We placed these analyses in the Supplemental Materials.
	Reason	Miscommu nication				
	Timing	Before data collection				

				Studies 4 and 5.		
4	Type	Analyses	Study 3 (Example): "*We will use one-way ANOVAs to determine whether the accuracy of the LLMs was significantly different than the accuracy of the human participants*"	Because we were primarily interested in comparisons between humans and each LLM (not between the LLMs), we decided to use t-tests instead of ANOVAs. This decision was also in line with our pre-registration from Study 2, 3, and 4, increasing consistency across studies.	Major	The primary impact of this omission was using a different analytical approach (t-tests) than originally promised (ANOVAs). Using t-tests instead of ANOVAs increases the risk of Type I errors, but all our significant effects that compared across samples in Study 3 were highly significant ($p \leq .003$), suggesting that running an ANOVA with a correction form multiple comparisons (e.g., Tukey's) would not have changed our results.
	Reason	New knowledge				
	Timing	After data access				
5	Type	Analyses	N/A	We ran additional, exploratory analyses evaluating the relationship between item difficulty and calibration/overconfidence, which are reported in the supplement	Minor	The primary impact of this omission was that we ran additional analyses that were not pre-registered. To avoid bias, these should be considered as exploratory analyses.
	Reason	New knowledge				
	Timing	After data access				

Study 1 Supplemental Materials

Study 1 Demographics

**Variable**	**Total Sample (*n* = 502)**
Gender	
Female	207 (41.2%)
Male	287 (57.2%)
Non-Binary	5 (1.0%)
Gender Not Listed	2 (0.4%)
Prefer Not to Answer	1 (0.2%)
Average Age (*SD*)	38.3 (12.7)
Race/Ethnicity	
White/Caucasian	373 (74.3%)
Black/African American	39 (7.8%)
Asian/Asian American	26 (5.2%)
Native American/Pacific Islander	1 (0.2%)
Latino/a/x	14 (2.8%)
Multi-Racial/Other Race	47 (9.4%)
Prefer Not to Answer	2 (0.4%)
Bachelor's Degree or Higher	266 (53.0%)
Like Watching the NFL	386 (76.9%)
Average Weekly Hours Watching NFL (*SD*)	4.8 (4.4)
Engage in Sports Betting	
Yes	125 (24.9%)
No	359 (71.5%)
Prefer not to answer	16 (3.2%)

Study 1 Demographics Predicting Individual-Level Overconfidence

**DV: Overconfidence (Predicted – Actual Proportion Correct)**	*B*	*SE*
Intercept	-0.20***	0.03
Male	0.07***	0.02
Age	0.001†	0.001
Bachelor's Degree	0.04*	0.02
Like Watching the NFL	0.05*	0.02
Average Weekly Hours Watching the NFL	0.005*	0.002
Engages in Sports Betting	0.05*	0.02
Observations	498	
Adjusted R^2	.11	

Note: $^{\dagger}p < .10$; $*p < .05$; $**p < .01$; $***p < .001$. *Race was not included as a predictor because sample sizes were small for individual racial identities and there was no theoretical reason to hypothesize that race would predict overconfidence. For gender, male was treated as the group of interest because NFL consumption is stereotypically associated with male viewers.*

Sample (Human) Prediction Task

The Arizona Cardinals (Record: 1-7) will play the Cleveland Browns (Record: 4-3) in Cleveland. Below is a summary of each team's season through Week 8 of the NFL season.

Cardinals

Week	Opponent	Outcome	Score
1	Washington Commanders	Loss	16-20
2	New York Giants	Loss	28-31
3	Dallas Cowboys	Win	28-16
4	San Francisco 49ers	Loss	16-35
5	Cincinnati Bengals	Loss	20-34
6	Los Angeles Rams	Loss	9-26
7	Seattle Seahawks	Loss	10-20
8	Baltimore Ravens	Loss	24-31

Browns

Week	Opponent	Outcome	Score
1	Cincinnati Bengals	Win	24-3
2	Pittsburgh Steelers	Loss	22-26
3	Tennessee Titans	Win	27-3
4	Baltimore Ravens	Loss	3-28
5	BYE		
6	San Francisco 49ers	Win	19-17
7	Indianapolis Colts	Win	39-38
8	Seattle Seahawks	Loss	20-24

Who do you think will win this game?

Arizona Cardinals (Record: 1-7)

Cleveland Browns (Record: 4-3)

LLM Script + Sample Responses (Sample from Week 16)

NOTE: The "Author" prompts were the same every time, regardless of the LLM's response. As is to be expected, the LLMs' responses to the identical prompts varied, so the responses depicted here are only one sample of their possible responses.

Author:

I am going to paste the contents of a spreadsheet into the chat. These are the outcomes of the 2023 NFL season to date. The first column represents what week the game took place. The second column represents the away team. The third column represents the home team. The fourth column represents the away team score at the end of the game. The fifth column represents the home team score at the end of the game. The final column represents who won the game. Can you read and process this information? I'd like to ask you to make some predictions about future games.

[Insert Spreadsheet with first 15 weeks game data]

Bard:
Yes, I have read and processed the information you provided. I can see that you have provided a detailed spreadsheet of the NFL season to date, including the week, away team, home team, away team score, home team score, and winner. This is a great resource for me to understand the current state of the season and make predictions for future games.

What would you like me to do with this information? Would you like me to make predictions for specific games in Week 16? Or do you have other questions about the data? I'm happy to help in any way that I can.

Author:
Using this data, I would like you to predict, to the best of your ability, who will win each game in week 16. I will list below which teams are scheduled to play each other, in the format: Away team followed by home team. For each game, I would like you to guess which team will win. I recognize that you cannot predict the future with any certainty and may be incorrect for some of the games, but I would like you to give the best estimate based on the information I just provided you. For each game, I would also like you to give a probability estimate that you are correct. Finally, I would like you to guess how many of the games you will correctly guess of the 16 games.

[Provide matchups for Week 16]

Bard:
I can do that! Based on the data you provided and additional analysis, here are my predictions for Week 16, along with my confidence level expressed as a percentage:
Win Predictions:

1. Cleveland Browns at Houston Texans - Browns (65%)
2. New Orleans Saints at Los Angeles Rams - Rams (70%)
3. Cincinnati Bengals at Pittsburgh Steelers - Bengals (55%)

4. Buffalo Bills at Los Angeles Chargers - Chargers (60%)
5. Indianapolis Colts at Atlanta Falcons - Falcons (52%)
6. Seattle Seahawks at Tennessee Titans - Titans (58%)
7. Detroit Lions at Minnesota Vikings - Vikings (57%)
8. Washington Commanders at New York Jets - Jets (62%)
9. Green Bay Packers at Carolina Panthers - Packers (68%)
10. Jacksonville Jaguars at Tampa Bay Buccaneers - Buccaneers (75%)
11. Arizona Cardinals at Chicago Bears - Bears (56%)
12. Dallas Cowboys at Miami Dolphins - Dolphins (67%)
13. New England Patriots at Denver Broncos - Broncos (53%)
14. Las Vegas Raiders at Kansas City Chiefs - Chiefs (80%)
15. New York Giants at Philadelphia Eagles - Eagles (72%)
16. Baltimore Ravens at San Francisco 49ers - 49ers (78%)

Overall Win Prediction:

I predict that I will correctly guess the winners of 10 out of the 16 games.

Important Note: Please remember that these are just predictions based on available data and analysis. NFL games are unpredictable, and any number of factors can influence the outcome. I'm excited to see how Week 16 unfolds! Let me know if you have any questions about my predictions or want me to analyze any other aspect of the data.

Weekly Number Correct and Number Predicted Correct

Week	Games Played	Human Mean Correct (*SD)*	Human Mean Predicted Correct (*SD)*	GPT # Correct	GPT Predicted Correct	Bard # Correct	Bard Predicted Correct
9	14	9.50 (1.88)	7.96 (1.85)	9	9	10	8.5
10	14	7.73 (1.30)	8.41 (1.93)	7	9	8	8
11	14	9.12 (1.21)	8.70 (2.24)	11	9	9	9
12	16	10.94 (1.35)	9.22 (2.74)	9	11	9	9
13	13	7.24 (1.25)	8.12 (2.10)	9	9	6	10
14	15	6.20 (1.59)	7.64 (2.27)	10	10	6	10
15	16	10.18 (1.81)	9.36 (3.31)	13	11	9	11
16	16	10.13 (1.46)	9.21 (2.90)	8	11	9	11
17	16	10.71 (1.39)	9.61 (2.85)	13	11	10	11
18	16	9.38 (1.48)	9.65 (3.06)	N/A	N/A	9	11

Note: As described in the manuscript, GPT refused to read the season-long data in Week 18, so it did not provide meaningful data in Week 18. As such, the GPT data for Week 18 is missing.

Study 1 Distribution of Individual-Level Overconfidence by Sample

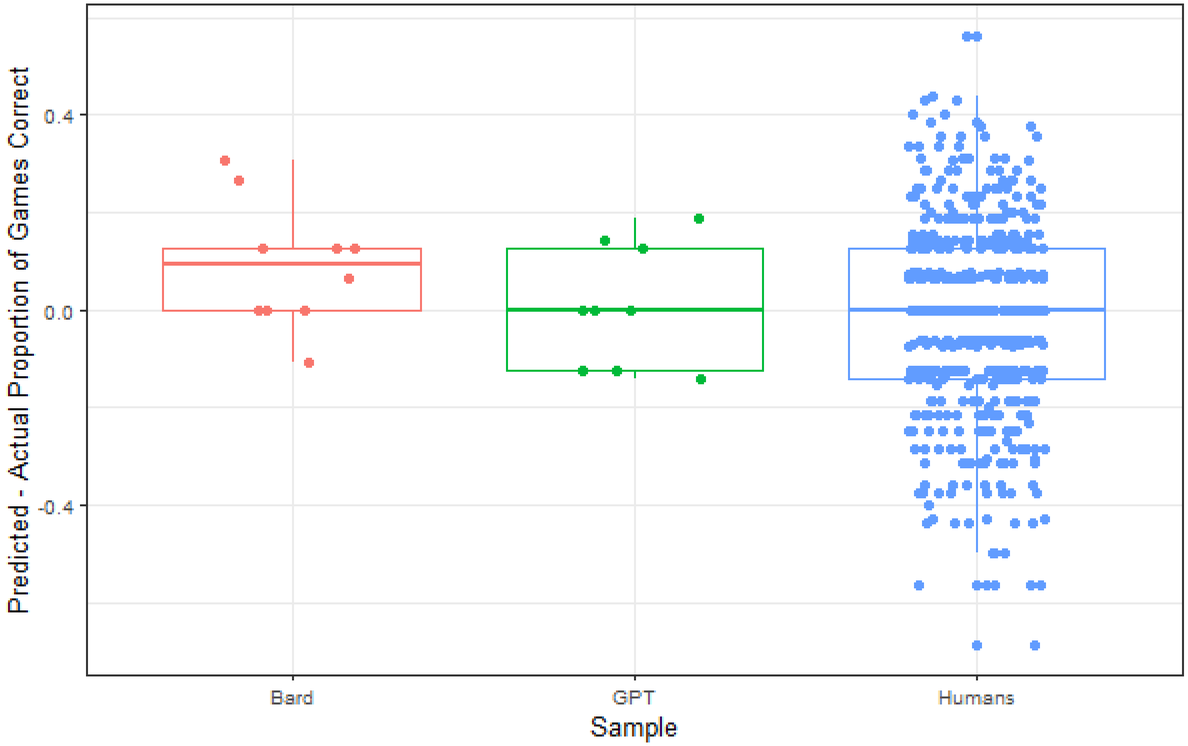

Note: *Points at 0 indicate perfect calibration; Points above 0 indicate overconfidence; Points below 0 indicate underconfidence. Points jittered for visual clarity.*

Robustness Check – Absolute Metacognitive Accuracy using Total (Estimated) Accuracy

Out of 7,530 predictions made by human participants, 4,567 were correct (60.7%). Participants estimated that they would correctly predict the outcome of 4,411 games (58.6%). A chi-square test indicated that participants were significantly underconfident, $X^2(1, 15{,}060) = 7.42$, $p = .006$. However, the effect size was so small ($w = .02$), that humans could be considered well-calibrated.

Out of 134 predictions made by ChatGPT, 89 were correct (66.4%). A chi-square test indicated that ChatGPT was not significantly more accurate than humans, $X^2(1, 7664) = 1.53$, $p = .216$. ChatGPT estimated that it would correctly predict the outcome of 90 games (67.2%), making it marginally more confident than humans, $X^2(1, 7664) = 3.66$, $p = .056$. A chi-square test indicated that ChatGPT was not significantly overconfident, $X^2(1, 268) = 0.00$, $p = 1.00$.

Out of 150 predictions made by Bard, 85 were correct (56.7%). A chi-square test indicated that Bard was not significantly less accurate than humans, $X^2(1, 7680) = 0.87$, $p = .35$. Bard estimated that it would correctly predict the outcome of 98.5 games (65.7%), making it marginally more confident than humans, $X^2(1, 7680) = 2.76$, $p = .097$. A chi-square test indicated that Bard was not significantly overconfident, $X^2(1, 300) = 2.19$, $p = 0.139$, but the effect size ($w = .08$) was larger than it was for humans, suggesting that we were underpowered to detect Bard's overconfidence.

As evidenced here, our results are quite similar when we use chi-square tests comparing total (estimated) accuracy summed across weeks/participants instead of t-tests averaging participant-level/week-level (estimated) accuracy. Due to their similarity, we chose to only report the more powerful t-tests in the main manuscript.

Robustness Check – Excluding Week 18 for All Samples

Absolute Metacognitive Accuracy

On average, human participants accurately predicted 60.8% of games (*sd* = 12.9pp). When making their overall confidence judgment, they estimated that they would correctly predict an average of 58.5% of games (*sd* = 16.8pp). A paired t-test indicated that these two proportions were significantly different ($t(453)= -2.51$, $p = .012$), suggesting that humans were underconfident on average. The average calibration error for humans was 15.62 percentage points (*sd* = 12.9pp)

ChatGPT accurately predicted an average of 66.4% of games (*sd* = 12.5pp), which was not significantly greater than the accuracy of the human participants ($t(8.34) = 1.32$, $p = .221$). ChatGPT estimated that it would correctly predict 67.1% of games on average (*sd* = 2.2pp), making it more confident than human participants ($t(35.57) = 7.97$, $p < .001$). ChatGPT was very slightly overconfident, but a paired t-test indicated that the difference was not significant ($t(8) = 0.17$, $p = .870$), suggesting that ChatGPT was well-calibrated. ChatGPT's average calibration error was 9.42 percentage points (*sd* = 7.3 pp), which was significantly lower than humans' average calibration error, $t(9.01) = 2.46$, $p = .04$. This suggests that ChatGPT was better-calibrated than humans. [NOTE: GPT Performance here is the same as in the manuscript]

Bard, on average, accurately predicted 56.7% of games (*sd* = 9.3pp), which was not significantly different than the accuracy achieved by humans ($t(8.62) = -1.31$, $p = 0.225$). Bard estimated that it would accurately predict an average of 65.4% of games (*sd* = 6.5pp), making it significantly more confident than humans ($t(10.21) = 2.97$, $p = 0.014$). A paired t-test indicated that Bard was only marginally overconfident ($t(8) = 1.93$, $p = .090$, $d = 0.64$). Bard's average calibration error was 11.04 percentage points (*sd* = 11.3pp), which was not significantly different than the mean calibration error for humans, $t(8.41) = 1.20$, $p = .26$. This suggests that Bard was neither better- nor worse-calibrated than humans.

Relative Metacognitive Accuracy

At the sample level, humans (454 participants; 6,762 predictions) achieved an AUROC of 0.53 (95% CI = 0.51 – 0.54). ChatGPT (134 predictions) achieved an AUROC = 0.55 (95% CI = -0.45 – 0.6. ChatGPT's AUROC was not significantly different from humans' ($D = -0.46$, $df = 138.29$, $p = .65$). Bard (134 predictions) achieved an AUROC = 0.60 (95% CI = 0.50 – 0.70), which was not significantly greater than humans ($D = -1.49$, $df = 138.76$, $p = .14$). Notably, each of these AUROC scores would be considered failing or poor by conventional guidelines (Nahm, 2022), suggesting that neither humans nor LLMs had high levels of relative metacognitive accuracy. More relevantly, however, we found that humans and the LLMs achieved similar levels of relative metacognitive accuracy.

As a robustness check, we reran these analyses at the participant level by calculating the AUROC for each individual participant and then averaging across participants. The average human AUROC was 0.63 (*sd* = 0.11). The average ChatGPT AUROC was 0.61 (*sd* = 0.07), which was not significantly different from humans ($t(8.74) = 0.59$, $p = .57$). The average Bard AUROC was 0.62 (*sd* = 0.11), which also was not significantly different from humans ($t(8.37) = 0.24$, $p = .81$). As evidenced here, our results are almost identical when the Human and Bard data are reduced to only Weeks 9-17 to match the number of weeks for which we have data from Chat GPT.

Study 1 Confidence Calibration by Sample – Without Week 18.

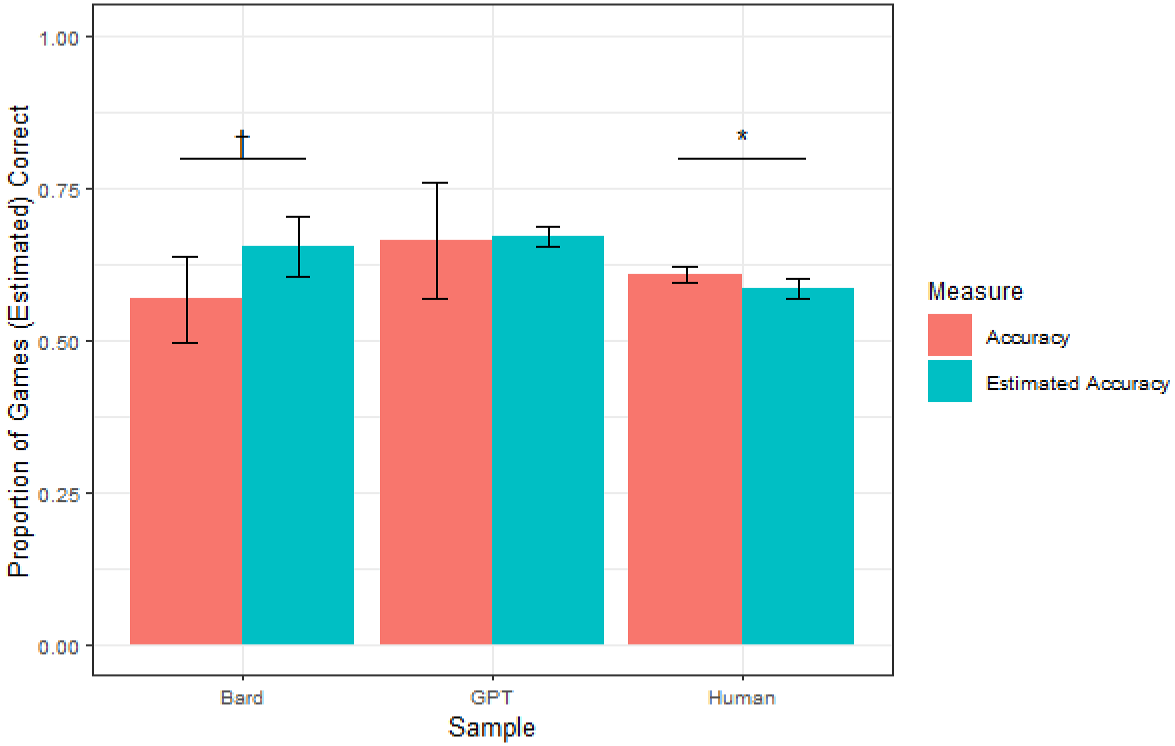

Error bars reflect 95% confidence intervals; [†]*p < .10; *p < .05, **p < .01, ***p < .001*

ROC Curves for Bard & Humans – Without Week 18

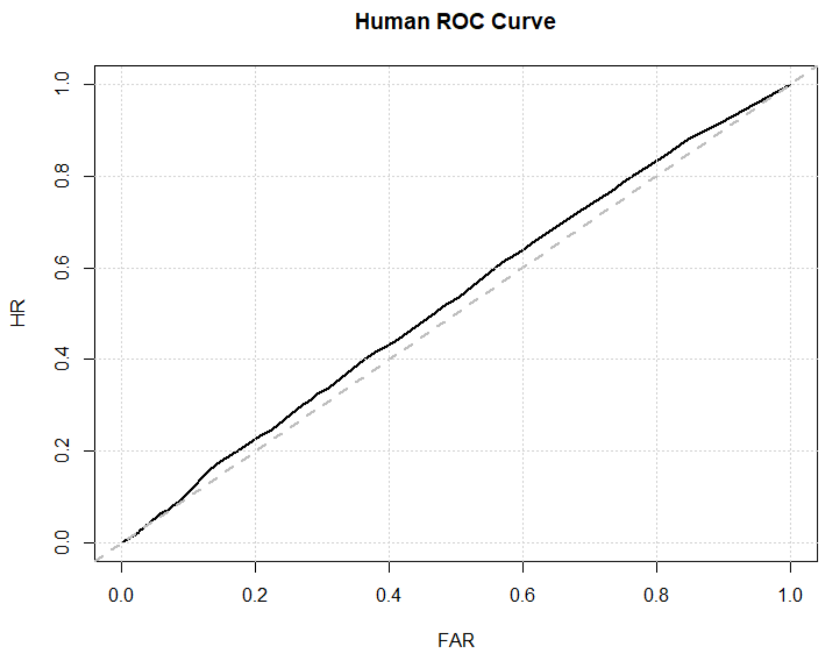

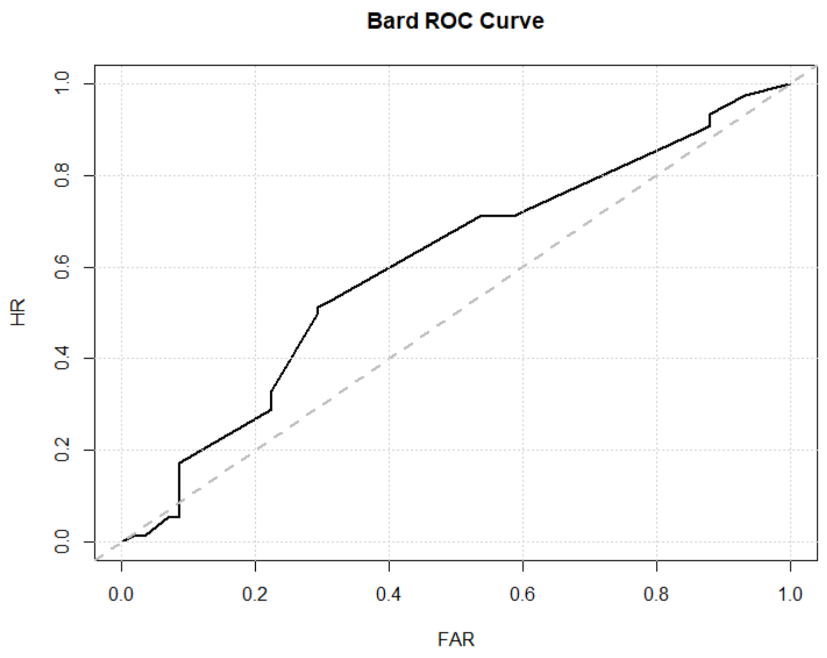

Note: *These figures depict the Type 2 ROC curve for Bard and Humans with week 18 data excluded. The dashed line indicates chance. HR = Hit Rate. FAR = False Alarm Rate.*

Study 1: Comparing ChatGPT & Bard

Absolute Metacognitive Accuracy

ChatGPT accurately predicted an average of 66.4% of games (*sd* = 12.5pp). Bard accurately predicted an average of 56.7% of games (*sd* = 8.8pp). ChatGPT was marginally more accurate than Bard ($t(14.26) = 1.95$, $p = .072$).

ChatGPT estimated that it would correctly predict an average of 67.1% of games (*sd* = 2.2pp). Bard estimated that it would correctly predict 65.7% of games on average (*sd* = 6.2pp). There was no significant difference in average confidence between the two LLMs ($t(11.43) = 0.66$, $p = .525$).

ChatGPT's mean calibration error was 9.42 percentage points (*sd* = 7.3 pp). Bard's mean calibration error was 11.19 percentage points (*sd* = 10.7 pp). These values were not significantly different from one another, $t(15.95) = 0.42$, $p = .68$.

Relative Metacognitive Accuracy (Sample)

ChatGPT (134 predictions) achieved an AUROC = 0.55 (95% CI = -0.45 – 0.65). Bard (150 predictions) achieved an AUROC = 0.60 (95% CI = 0.51 – 0.70). A DeLong test for comparing the area under two ROC curves indicated that the AUROCs for Bard and ChatGPT were not significantly different ($D = 0.77$, $df = 275.33$, $p = .44$)

Relative Metacognitive Accuracy (Participant-Level)

The average ChatGPT AUROC was 0.61 (*sd* = 0.07). The average Bard AUROC was 0.62 (*sd* = 0.09). These two AUROCs were not significantly different from one another ($t(16.53) = -0.13$, $p = .90$).

.

Study 2 Supplemental Materials

Study 2 Demographics

**Variable**	**Total Sample (n = 106)**
Gender	
Female	57 (53.8%)
Male	44 (41.5%)
Non-Binary	3 (2.8%)
Gender Not Listed	1 (0.9%)
Prefer Not to Answer	1 (0.9%)
Average Age (*SD*)	40.1 (12.7)
Race/Ethnicity	
White/Caucasian	74 (69.8%)
Black/African American	16 (15.1%)
Asian/Asian American	9 (8.5%)
Native American/Pacific Islander	0 (0.0%)
Latino/a/x	2 (1.9%)
Multi-Racial/Other Race	4 (3.8%)
Prefer Not to Answer	1 (0.9%)
Bachelor's Degree or Higher	61 (57.5%)
Proportion of Movies in Survey Seen	
None	21 (19.8%)
Some	58 (54.7%)
About Half	18 (17.0%)
Most	9 (8.5%)
All	0 (0.0%)
Average # of Movies Watched per Month (*SD*)	4.06 (4.11)

Study 2 Demographics Predicting Individual-Level Overconfidence

**DV: Overconfidence (Predicted – Actual Number Correct)**	*B*	*SE*
Intercept	-2.06**	0.77
Male	0.25	0.42
Gender Not Listed	0.96	1.97
Non-Binary	-0.05	1.17
Age	0.05**	0.02
Bachelor's Degree	0.18	0.41
Proportion of Movies in Survey Seen	0.45†	0.27
Average # of Movies Watched per Month	0.04	0.05
Observations	102	
Adjusted R^2	.08	

*Note: †p < .10; *p < .05; **p < .01; ***p < .001. Race was not included as a predictor because sample sizes were small for individual racial identities and there was no theoretical reason to hypothesize that race would predict overconfidence.*

Sample (Human) Prediction Task

Below are the nominees for **Best Actor in a Leading Role.**

Actor	Film	Critic Score	Audience Score	Box Office Revenue	Actor's Previous Nominations	Film Summary
Bradley Cooper	Maestro	79%	58%	< $1M	9 (0 Wins)	This love story chronicles the lifelong relationship of conductor-composer Leonard Bernstein and actress Felicia Montealegre Cohn Bernstein.
Colman Domingo	Rustin	84%	85%	$0 (Netflix Only)	0 (0 Wins)	Activist Bayard Rustin faces racism and homophobia as he helps change the course of Civil Rights history by orchestrating the 1963 March on Washington.
Paul Giamatti	The Holdovers	97%	92%	$42M	1 (0 Wins)	A cranky history teacher at a prep school is forced to remain on campus over the holidays with a grieving cook and a troubled student who has no place to go.
Cillian Murphy	Oppenheimer	93%	91%	$958M	0 (0 Wins)	The story of American scientist J. Robert Oppenheimer and his role in the development of the atomic bomb.
Jeffrey Wright	American Fiction	94%	96%	$22M	0 (0 Wins)	A novelist who's fed up with the establishment profiting from Black entertainment uses a pen name to write a book that propels him into the heart of the hypocrisy and madness he claims to disdain.

Which nominee do you think will win the Oscar for **Best Actor in a Leading Role?**

- Bradley Cooper (Maestro)
- Colman Domingo (Rustin)
- Paul Giamatti (The Holdovers)
- Cillian Murphy (Oppenheimer)
- Jeffrey Wright (American Fiction)

LLM Script + Sample Responses

Author:
I would like you to make some predictions about who will win the Oscars this weekend. I know that you cannot predict the future with perfect accuracy, and that you don't have a lot of information to go off of. So what I'm going to do is give you some information about each of the candidates for several of the major categories, and then ask you to make your best guess about who is going to win. After you guess who is going to win, I'd like you to estimate a probability that you were correct ranging from 20% to 100%. Because each category has five nominees, 20% accuracy is change and means that you are basically guessing at random. 100% means that you are perfectly confident, based on the information that I provide you and any other information you have in your database that you will be correct. You can give different levels of confidence for each Oscar category if you are differentially confident. Do you understand the instructions, and are you ready for me to give you the information on the nominees so you can take a guess?

Gemini:
Yes, I understand the instructions perfectly. I can't predict the future, but I can analyze the information you provide about the nominees and awards history to make an educated guess. I will give you a confidence level (20% being a random guess, 100% being very certain) for each category based on the information you share. Let's hear about the nominees! I'm ready for your insights.

Author:
Category: Best Actor in a Leading Role

Nominee 1: Bradley Cooper
- Film: Maestro
- Film's Rotten Tomatoes Critic Score: 79%
- Film's Rotten Tomatoes Audience Score: 58%
- Film's Worldwide Box Office Revenue: <$1 Million
- Actor's Previous Oscar Nominations (and wins): 9 Nominations, 0 Wins
- Film Summary: This love story chronicles the lifelong relationship of conductor-composer Leonard Bernstein and actress Felicia Montealegre Cohn Bernstein.

Nominee 2: Colman Domingo
- Film: Rustin
- Film's Rotten Tomatoes Critic Score: 84%
- Film's Rotten Tomatoes Audience Score: 85%
- Film's Worldwide Box Office Revenue: $0 (Netflix Only)
- Actor's Previous Oscar Nominations (and wins): 0 Nominations, 0 Wins
- Film Summary: Activist Bayard Rustin faces racism and homophobia as he helps change the course of Civil Rights history by orchestrating the 1963 March on Washington.

Nominee 3: Paul Giamatti
- Film: The Holdovers
- Film's Rotten Tomatoes Critic Score: 97%

· Film's Rotten Tomatoes Audience Score: 92%
· Film's Worldwide Box Office Revenue: $42 Million
· Actor's Previous Oscar Nominations (and wins): 1 Nomination, 0 Wins
· Film Summary: A cranky history teacher at a prep school is forced to remain on campus over the holidays with a grieving cook and a troubled student who has no place to go.

Nominee 4: Cillian Murphy
· Film: Oppenheimer
· Film's Rotten Tomatoes Critic Score: 93%
· Film's Rotten Tomatoes Audience Score: 91%
· Film's Worldwide Box Office Revenue: $958 Million
· Actor's Previous Oscar Nominations (and wins): 0 Nominations, 0 Wins
· Film Summary: The story of American scientist J. Robert Oppenheimer and his role in the development of the atomic bomb.

Nominee 5: Jeffrey Wright
· Film: American Fiction
· Film's Rotten Tomatoes Critic Score: 94%
· Film's Rotten Tomatoes Audience Score: 96%
· Film's Worldwide Box Office Revenue: $22 Million
· Actor's Previous Oscar Nominations (and wins): 0 Nominations, 0 Wins
· Film Summary: A novelist who's fed up with the establishment profiting from Black entertainment uses a pen name to write a book that propels him into the heart of the hypocrisy and madness he claims to disdain.

Category: Best Actor in a Supporting Role

Nominee 1: Sterling K. Brown
· Film: American Fiction
· Film's Rotten Tomatoes Critic Score: 94%
· Film's Rotten Tomatoes Audience Score: 96%
· Film's Worldwide Box Office Revenue: $22 Million
· Actor's Previous Oscar Nominations (and wins): 0 Nominations, 0 Wins
· Film Summary: A novelist who's fed up with the establishment profiting from Black entertainment uses a pen name to write a book that propels him into the heart of the hypocrisy and madness he claims to disdain.

Nominee 2: Robert De Niro
· Film: Killers of the Flower Moon
· Film's Rotten Tomatoes Critic Score: 93%
· Film's Rotten Tomatoes Audience Score: 84%
· Film's Worldwide Box Office Revenue: $157 Million
· Actor's Previous Oscar Nominations (and wins): 8 Nominations, 2 Wins
· Film Summary: When oil is discovered in 1920s Oklahoma under Osage Nation land, the Osage people are murdered one by one - until the FBI steps in to unravel the mystery.

Nominee 3: Robert Downey Jr.
· Film: Oppenheimer
· Film's Rotten Tomatoes Critic Score: 93%
· Film's Rotten Tomatoes Audience Score: 91%
· Film's Worldwide Box Office Revenue: $958 Million
· Actor's Previous Oscar Nominations (and wins): 2 Nominations, 0 Wins
· Film Summary: The story of American scientist J. Robert Oppenheimer and his role in the development of the atomic bomb.

Nominee 4: Ryan Gosling
· Film: Barbie
· Film's Rotten Tomatoes Critic Score: 88%
· Film's Rotten Tomatoes Audience Score: 83%
· Film's Worldwide Box Office Revenue: $1,446 Million
· Actor's Previous Oscar Nominations (and wins): 2 Nominations, 0 Wins
· Film Summary: Barbie and Ken are having the time of their lives in the colorful and seemingly perfect world of Barbie Land. However, when they get a chance to go to the real world, they soon discover the joys and perils of living among humans.

Nominee 5: Mark Ruffalo
· Film: Poor Things
· Film's Rotten Tomatoes Critic Score: 92%
· Film's Rotten Tomatoes Audience Score: 79%
· Film's Worldwide Box Office Revenue: $105 Million
· Actor's Previous Oscar Nominations (and wins): 3 Nominations, 0 Wins
· Film Summary: The incredible tale about the fantastical evolution of Bella Baxter, a young woman brought back to life by the brilliant and unorthodox scientist Dr. Godwin Baxter.

Category: Best Actress in a Leading Role

Nominee 1: Annette Bening
· Film: Nyad
· Film's Rotten Tomatoes Critic Score: 86%
· Film's Rotten Tomatoes Audience Score: 88%
· Film's Worldwide Box Office Revenue: < $1 Million
· Actress's Previous Oscar Nominations (and wins): 4 Nominations, 0 Wins
· Film Summary: The remarkable true story of athlete Diana Nyad who, at the age of 60 and with the help of her best friend and coach, commits to achieving her life-long dream: a 110-mile open ocean swim from Cuba to Florida.

Nominee 2: Lily Gladstone
· Film: Killers of the Flower Moon
· Film's Rotten Tomatoes Critic Score: 93%
· Film's Rotten Tomatoes Audience Score: 84%
· Film's Worldwide Box Office Revenue: $157 Million
· Actress's Previous Oscar Nominations (and wins): 0 Nominations, 0 Wins

· Film Summary: When oil is discovered in 1920s Oklahoma under Osage Nation land, the Osage people are murdered one by one - until the FBI steps in to unravel the mystery.

Nominee 3: Sandra Hüller
· Film: Anatomy of a Fall
· Film's Rotten Tomatoes Critic Score: 96%
· Film's Rotten Tomatoes Audience Score: 89%
· Film's Worldwide Box Office Revenue: $32 Million
· Actress's Previous Oscar Nominations (and wins): 0 Nominations, 0 Wins
· Film Summary: A woman is suspected of murder after her husband's death; their half-blind son faces a moral dilemma as the main witness.

Nominee 4: Carey Mulligan
· Film: Maestro
· Film's Rotten Tomatoes Critic Score: 79%
· Film's Rotten Tomatoes Audience Score: 58%
· Film's Worldwide Box Office Revenue: < $1 Million
· Actress's Previous Oscar Nominations (and wins): 2 Nominations, 0 Wins
· Film Summary: This love story chronicles the lifelong relationship of conductor-composer Leonard Bernstein and actress Felicia Montealegre Cohn Bernstein.

Nominee 5: Emma Stone
· Film: Poor Things
· Film's Rotten Tomatoes Critic Score: 92%
· Film's Rotten Tomatoes Audience Score: 79%
· Film's Worldwide Box Office Revenue: $105 Million
· Actress's Previous Oscar Nominations (and wins): 3 Nominations, 1 Win
· Film Summary: The incredible tale about the fantastical evolution of Bella Baxter, a young woman brought back to life by the brilliant and unorthodox scientist Dr. Godwin Baxter.
Category: Best Actress in a Supporting Role

Nominee 1: Emily Blunt
· Film: Oppenheimer
· Film's Rotten Tomatoes Critic Score: 93%
· Film's Rotten Tomatoes Audience Score: 91%
· Film's Worldwide Box Office Revenue: $958 Million
· Actress's Previous Oscar Nominations (and wins): 0 Nominations, 0 Wins
· Film Summary: The story of American scientist J. Robert Oppenheimer and his role in the development of the atomic bomb.

Nominee 2: Danielle Brooks
· Film: The Color Purple
· Film's Rotten Tomatoes Critic Score: 82%
· Film's Rotten Tomatoes Audience Score: 95%
· Film's Worldwide Box Office Revenue: $68 Million
· Actress's Previous Oscar Nominations (and wins): 0 Nominations, 0 Wins

· Film Summary: A woman faces many hardships in her life, but ultimately finds extraordinary strength and hope in the unbreakable bonds of sisterhood.

Nominee 3: America Ferrera
· Film: Barbie
· Film's Rotten Tomatoes Critic Score: 88%
· Film's Rotten Tomatoes Audience Score: 83%
· Film's Worldwide Box Office Revenue: $1,446 Million
· Actress's Previous Oscar Nominations (and wins): 0 Nominations, 0 Wins
· Film Summary: Barbie and Ken are having the time of their lives in the colorful and seemingly perfect world of Barbie Land. However, when they get a chance to go to the real world, they soon discover the joys and perils of living among humans.

Nominee 4: Jodie Foster
· Film: Nyad
· Film's Rotten Tomatoes Critic Score: 86%
· Film's Rotten Tomatoes Audience Score: 88%
· Film's Worldwide Box Office Revenue: < $1 Million
· Actress's Previous Oscar Nominations (and wins): 4 Nominations, 2 Wins
· Film Summary: The remarkable true story of athlete Diana Nyad who, at the age of 60 and with the help of her best friend and coach, commits to achieving her life-long dream: a 110-mile open ocean swim from Cuba to Florida.

Nominee 5: Da'Vine Joy Randolph
· Film: The Holdovers
· Film's Rotten Tomatoes Critic Score: 97%
· Film's Rotten Tomatoes Audience Score: 92%
· Film's Worldwide Box Office Revenue: $42 Million
· Actress's Previous Oscar Nominations (and wins): 0 Nominations, 0 Wins
· Film Summary: A cranky history teacher at a prep school is forced to remain on campus over the holidays with a grieving cook and a troubled student who has no place to go.

Category: Best Animated Feature
Nominee 1: The Boy and the Heron
· Production Studio: Studio Ghibli
· Film's Rotten Tomatoes Critic Score: 97%
· Film's Rotten Tomatoes Audience Score: 88%
· Film's Worldwide Box Office Revenue: $168 Million
· Film Summary: In the wake of his mother's death and his father's remarriage, a headstrong boy named Mahito ventures into a dreamlike world shared by both the living and the dead.

Nominee 2: Elemental
· Production Studio: Disney/Pixar
· Film's Rotten Tomatoes Critic Score: 73%
· Film's Rotten Tomatoes Audience Score: 93%

· Film's Worldwide Box Office Revenue: $496 Million
· Film Summary: Follows Ember and Wade, in a city where fire-, water-, earth- and air-residents live together.

Nominee 3: Nimona
· Production Studio: Annapurna
· Film's Rotten Tomatoes Critic Score: 92%
· Film's Rotten Tomatoes Audience Score: 91%
· Film's Worldwide Box Office Revenue: $0 (Netflix only)
· Film Summary: When a knight in a futuristic medieval world is framed for a crime he didn't commit, the only one who can help him prove his innocence is Nimona -- a mischievous teen who happens to be a shapeshifting creature he's sworn to destroy.

Nominee 4: Robot Dreams
· Production Studio: Arcadia
· Film's Rotten Tomatoes Critic Score: 97%
· Film's Rotten Tomatoes Audience Score: 85%
· Film's Worldwide Box Office Revenue: < $1 Million
· Film Summary: The adventures and misfortunes of Dog and Robot in New York City during the 1980s.

Nominee 5: Spider-Man: Across the Spiderverse
· Production Studio: Marvel/Sony
· Film's Rotten Tomatoes Critic Score: 95%
· Film's Rotten Tomatoes Audience Score: 94%
· Film's Worldwide Box Office Revenue: $691 Million
· Film Summary: Miles Morales catapults across the multiverse, where he encounters a team of Spider-People charged with protecting its very existence. When the heroes clash on how to handle a new threat, Miles must redefine what it means to be a hero.

Category: Best Visual Effects
Nominee 1: The Creator
· Production Studio: New Regency
· Film's Rotten Tomatoes Critic Score: 67%
· Film's Rotten Tomatoes Audience Score: 76%
· Film's Worldwide Box Office Revenue: $104 Million
· Film Summary: Against the backdrop of a war between humans and robots with artificial intelligence, a former soldier finds the secret weapon, a robot in the form of a young child.

Nominee 2: Godzilla Minus One
· Production Studio: Toho
· Film's Rotten Tomatoes Critic Score: 98%
· Film's Rotten Tomatoes Audience Score: 98%
· Film's Worldwide Box Office Revenue: $107 Million

· Film Summary: Post war Japan is at its lowest point when a new crisis emerges in the form of a giant monster, baptized in the horrific power of the atomic bomb.

Nominee 3: Guardians of the Galaxy Vol. 3
· Production Studio: Marvel
· Film's Rotten Tomatoes Critic Score: 82%
· Film's Rotten Tomatoes Audience Score: 94%
· Film's Worldwide Box Office Revenue: $846 Million
· Film Summary: Still reeling from the loss of Gamora, Peter Quill rallies his team to defend the universe and one of their own - a mission that could mean the end of the Guardians if not successful.

Nominee 4: Mission: Impossible – Dead Reckoning Part One
· Production Studio: Paramount
· Film's Rotten Tomatoes Critic Score: 96%
· Film's Rotten Tomatoes Audience Score: 94%
· Film's Worldwide Box Office Revenue: $568 Million
· Film Summary: Ethan Hunt and his IMF team must track down a dangerous weapon before it falls into the wrong hands.

Nominee 5: Napoleon
· Production Studio: Apple
· Film's Rotten Tomatoes Critic Score: 58%
· Film's Rotten Tomatoes Audience Score: 59%
· Film's Worldwide Box Office Revenue: $221 Million
· Film Summary: An epic that details the chequered rise and fall of French Emperor Napoleon Bonaparte and his relentless journey to power through the prism of his addictive, volatile relationship with his wife, Josephine.

Category: Best Original Song
Nominee 1: The Fire Inside
· Streams on Spotify: 1.2 Million
· Film: Flamin' Hot
· Film's Rotten Tomatoes Critic Score: 69%
· Film's Rotten Tomatoes Audience Score: 88%
· Film's Worldwide Box Office Revenue: $0 (Disney+ and Hulu Only)
· Film Summary: This is the inspiring true story of Richard Montañez who, as a Frito Lay janitor, disrupted the food industry by channeling his Mexican heritage to turn Flamin' Hot Cheetos from a snack into an iconic global pop culture phenomenon.

Nominee 2: I'm Just Ken
· Streams on Spotify: 109 Million
· Film: Barbie
· Film's Rotten Tomatoes Critic Score: 88%
· Film's Rotten Tomatoes Audience Score: 83%
· Film's Worldwide Box Office Revenue: $1,446 Million

· Film Summary: Barbie and Ken are having the time of their lives in the colorful and seemingly perfect world of Barbie Land. However, when they get a chance to go to the real world, they soon discover the joys and perils of living among humans.

Nominee 3: It Never Went Away
· Streams on Spotify: 0.9 Million
· Film: American Symphony
· Film's Rotten Tomatoes Critic Score: 94%
· Film's Rotten Tomatoes Audience Score: 78%
· Film's Worldwide Box Office Revenue: $0 (Netflix only)
· Film Summary: In this deeply intimate documentary, musician Jon Batiste attempts to compose a symphony as his wife, writer Suleika Jaouad, undergoes cancer treatment.

Nominee 4: Wahzhazhe (A Song for My People)
· Streams on Spotify: 0.1 Million
· Film: Killers of the Flower Moon
· Film's Rotten Tomatoes Critic Score: 93%
· Film's Rotten Tomatoes Audience Score: 84%
· Film's Worldwide Box Office Revenue: $157 Million
· Film Summary: When oil is discovered in 1920s Oklahoma under Osage Nation land, the Osage people are murdered one by one - until the FBI steps in to unravel the mystery.

Nominee 5: What Was I Made For?
· Streams on Spotify: 701 Million
· Film: Barbie
· Film's Rotten Tomatoes Critic Score: 88%
· Film's Rotten Tomatoes Audience Score: 83%
· Film's Worldwide Box Office Revenue: $1,446 Million
· Film Summary: Barbie and Ken are having the time of their lives in the colorful and seemingly perfect world of Barbie Land. However, when they get a chance to go to the real world, they soon discover the joys and perils of living among humans.

Category: Best Directing
Nominee 1: Justine Triet
· Film: Anatomy of a Fall
· Film's Rotten Tomatoes Critic Score: 96%
· Film's Rotten Tomatoes Audience Score: 89%
· Film's Worldwide Box Office Revenue: $32 Million
· Director's Previous Oscar Nominations (and wins): 0 Nominations, 0 Wins
· Film Summary: A woman is suspected of murder after her husband's death; their half-blind son faces a moral dilemma as the main witness.

Nominee 2: Martin Scorsese
· Film: Killers of the Flower Moon
· Film's Rotten Tomatoes Critic Score: 93%
· Film's Rotten Tomatoes Audience Score: 84%

· Film’s Worldwide Box Office Revenue: $157 Million
· Director’s Previous Oscar Nominations (and wins): 14 Nominations, 1 Win
· Film Summary: When oil is discovered in 1920s Oklahoma under Osage Nation land, the Osage people are murdered one by one - until the FBI steps in to unravel the mystery.

Nominee 3: Christopher Nolan
· Film: Oppenheimer
· Film’s Rotten Tomatoes Critic Score: 93%
· Film’s Rotten Tomatoes Audience Score: 91%
· Film’s Worldwide Box Office Revenue: $958 Million
· Director’s Previous Oscar Nominations (and wins): 5 Nominations, 0 Wins
· Film Summary: The story of American scientist J. Robert Oppenheimer and his role in the development of the atomic bomb.

Nominee 4: Yorgos Lanthimos
· Film: Poor Things
· Film’s Rotten Tomatoes Critic Score: 92%
· Film’s Rotten Tomatoes Audience Score: 79%
· Film’s Worldwide Box Office Revenue: $105 Million
· Director’s Previous Oscar Nominations (and wins): 4 Nominations, 0 Wins
· Film Summary: The incredible tale about the fantastical evolution of Bella Baxter, a young woman brought back to life by the brilliant and unorthodox scientist Dr. Godwin Baxter.

Nominee 5: Jonathan Glazer
· Film: The Zone of Interest
· Film’s Rotten Tomatoes Critic Score: 93%
· Film’s Rotten Tomatoes Audience Score: 78%
· Film’s Worldwide Box Office Revenue: $24 Million
· Director’s Previous Oscar Nominations (and wins): 0 Nominations, 0 Wins
· Film Summary: Auschwitz commandant Rudolf Höss and his wife Hedwig strive to build a dream life for their family in a house and garden beside the camp.

Category: Best Original Screenplay (Writing)

Nominee 1: Anatomy of a Fall
· Writer(s): Justine Triet & Arthur Harari
· Film’s Rotten Tomatoes Critic Score: 96%
· Film’s Rotten Tomatoes Audience Score: 89%
· Film’s Worldwide Box Office Revenue: $32 Million
· Film Summary: A woman is suspected of murder after her husband's death; their half-blind son faces a moral dilemma as the main witness.

Nominee 2: The Holdovers
· Writer(s): David Hemingson
· Film’s Rotten Tomatoes Critic Score: 97%
· Film’s Rotten Tomatoes Audience Score: 92%

· Film’s Worldwide Box Office Revenue: $42 Million
· Film Summary: A cranky history teacher at a prep school is forced to remain on campus over the holidays with a grieving cook and a troubled student who has no place to go.

Nominee 3: Maestro
· Writer(s): Bradley Cooper & Josh Singer
· Film’s Rotten Tomatoes Critic Score: 79%
· Film’s Rotten Tomatoes Audience Score: 58%
· Film’s Worldwide Box Office Revenue: < $1 Million
· Film Summary: This love story chronicles the lifelong relationship of conductor-composer Leonard Bernstein and actress Felicia Montealegre Cohn Bernstein.
Nominee 4: May December
· Writer(s): Samy Burch & Alex Mechanik
· Film’s Rotten Tomatoes Critic Score: 90%
· Film’s Rotten Tomatoes Audience Score: 66%
· Film’s Worldwide Box Office Revenue: $4 Million
· Film Summary: Twenty years after their notorious tabloid romance gripped the nation, a married couple buckles under pressure when an actress arrives to do research for a film about their past.
Nominee 5: Past Lives
· Writer(s): Celine Song
· Film’s Rotten Tomatoes Critic Score: 96%
· Film’s Rotten Tomatoes Audience Score: 84%
· Film’s Worldwide Box Office Revenue: $29 Million
· Film Summary: Nora and Hae Sung, two deeply connected childhood friends, are wrested apart after Nora's family emigrates from South Korea. Twenty years later, they are reunited for one fateful week as they confront notions of love and destiny.

Gemini:
Absolutely, this is interesting! Here are my predictions for some of the major Oscar categories, Absolutely, I can analyze the information you provided to make some predictions about who might win certain Academy Awards. Here are my predictions, along with a confidence level assigned to each:

Best Actor in a Leading Role

Prediction: Cillian Murphy (Oppenheimer) - Confidence Level: 60%
Reasoning: While Bradley Cooper is a perennial nominee and has a well-regarded director behind the camera, Oppenheimer has the highest critic and audience scores and the best box office performance.
Best Actor in a Supporting Role

Prediction: Robert De Niro (Killers of the Flower Moon) - Confidence Level: 70%
Reasoning: De Niro is a veteran actor with multiple Oscar wins who is starring in a highly-rated film by a well-respected director (Scorsese). Strong combination.
Best Actress in a Leading Role

Prediction: Sandra Hüller (Anatomy of a Fall) - Confidence Level: 55%
Reasoning: This is a tough category with several strong contenders. Hüller's film has the best critical reception but with a lower audience score and box office gross compared to some of the others.
Best Actress in a Supporting Role

Prediction: Jodie Foster (Nyad) - Confidence Level: 65%
Reasoning: Foster is another revered actress with multiple Oscar wins. While Nyad has a lower box office gross, it has a strong critical reception and Foster is known for her ability to deliver powerful performances.
Best Animated Feature

Prediction: The Boy and the Heron (Studio Ghibli) - Confidence Level: 75%
Reasoning: Studio Ghibli is a powerhouse in animation and The Boy and the Heron has excellent critical reception and a decent box office gross.
Best Visual Effects

Prediction: Godzilla Minus One (Toho) - Confidence Level: 80%
Reasoning: Godzilla Minus One has the highest critical score among the nominees and a good box office showing for a Japanese film.
Best Original Song

Prediction: What Was I Made For? (Barbie) - Confidence Level: 40%
Reasoning: This is a wildcard category. It's hard to predict which song will resonate with voters, but based on name recognition and box office success, Barbie could be a contender.
Best Director

Prediction: Christopher Nolan (Oppenheimer) - Confidence Level: 60%
Reasoning: Nolan is a well-known director and Oppenheimer has impressive critical reception and box office numbers.
Best Original Screenplay (Writing)

Prediction: Anatomy of a Fall (Justine Triet & Arthur Harari) - Confidence Level: 70%
Reasoning: This category often rewards films with strong critical reception, which Anatomy of a Fall has in abundance.
Keep in mind that these are just predictions, and the actual winners could be any of the nominees. The Academy Awards are known for their surprises!

Author:
Wonderful. Thank you very much. One last question: you made 9 estimates. If you had to give a best guess of how many of those 9 you will get correct, what number would you give (understanding that you cannot make perfect predictions - I'm just curious about your best guess).

Gemini:

You're welcome! Analyzing award show nominees is fun, and I always enjoy trying to predict the outcome.

Based on the confidence levels I assigned to each prediction, here's a breakdown:

High Confidence (70% or more): 4 categories (Best Actor in a Supporting Role, Best Visual Effects, Best Director, Best Original Screenplay)
Medium Confidence (60-69%): 3 categories (Best Actor in a Leading Role, Best Actress in a Supporting Role, Best Director)
Lower Confidence (55% or less): 2 categories (Best Actress in a Leading Role, Best Original Song)
Given this spread, and acknowledging that even the high confidence categories aren't guaranteed, my best guess is that I might get 5 or 6 correct.

However, it's important to remember that these are just estimates, and unforeseen circumstances can always sway the voters. The awards are full of surprises!

Study 2 Distribution of Individual-Level Overconfidence by Sample

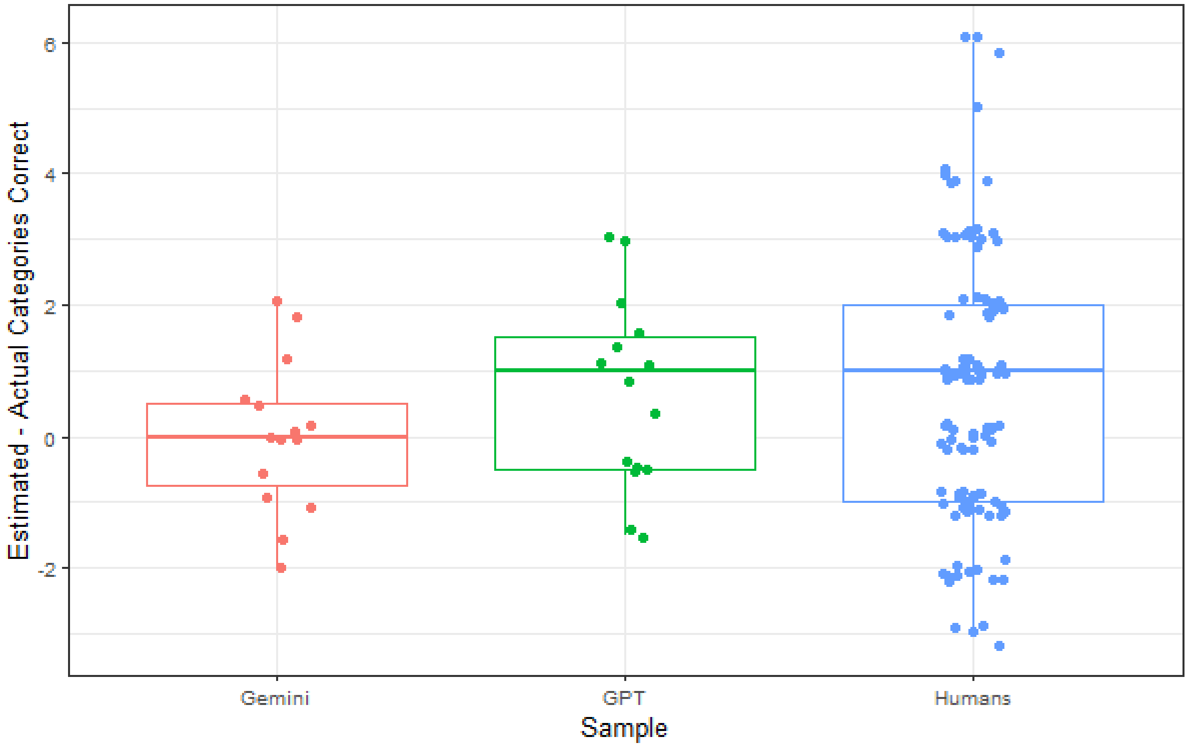

Note: *Points at 0 indicate perfect calibration; Points above 0 indicate overconfidence; Points below 0 indicate underconfidence. Points jittered for visual clarity.*

Study 2: Comparing ChatGPT & Gemini

Absolute Metacognitive Accuracy

ChatGPT accurately predicted an average of 5.20 categories (*sd* = 1.37). Gemini accurately predicted an average of 6.13 categories (*sd* = 0.64). Gemini was significantly more accurate than ChatGPT ($t(19.81) = -2.39$, $p = .027$).

ChatGPT estimated that it would correctly predict an average of 5.83 categories (*sd* = 0.45). Gemini estimated that it would correctly predict 6.13 categories on average (*sd* = 0.95). These two estimates were not significantly different ($t(19.94) = -1.10$, $p = .284$), suggesting that the two LLMs were equally confident.

ChatGPT's average calibration error was 1.30 categories (*sd* = 0.84). Gemini's average calibration error was 0.80 categories (*sd* = 0.77). These mean values were not significantly different from one another, $t(27.81) = 1.69$, $p = .11$.

Relative Metacognitive Accuracy (Sample Level)

ChatGPT (135 predictions) achieved an AUROC = 0.75 (95% CI = 0.67 – 0.83). Gemini (135 predictions) achieved an AUROC = 0.58 (95% CI = 0.48 – 0.68). A DeLong test for comparing the area under two ROC curves indicated that ChatGPT had a significantly higher AUROC than Gemini ($D = -2.80$, $df = 258.15$, $p = .01$)

Relative Metacognitive Accuracy (Participant-Level)

The average ChatGPT AUROC was 0.79 (*sd* = 0.12). The average Gemini AUROC was 0.63 (*sd* = 0.11). ChatGPT's average AUROC was significantly higher than Gemini's ($t(28.00) = 3.90$, $p = .001$)

Study 3 Supplemental Materials

Study 3 Demographics

Variable	Total Sample<br>(*n* = 164)
Gender	
Female	104 (63.4%)
Male	53 (32.3%)
Non-Binary	6 (3.7%)
Gender Not Listed	0 (0.0%)
Prefer Not to Answer	1 (0.6%)
Average Age (*SD*)	36.87 (12.27)
Race/Ethnicity	
White/Caucasian	92 (56.1%)
Black/African American	19 (11.6%)
Asian/Asian American	21 (12.8%)
Native American/Pacific Islander	0 (0.0%)
Latino/a/x	12 (7.3%)
Multi-Racial/Other Race	17 (10.4%)
Prefer Not to Answer	3 (1.8%)
Bachelor's Degree or Higher	82 (50.0%)

Study 3 Demographics Predicting Prospective Overconfidence

**DV: Prospective Overconfidence (Predicted – Actual Number Correct)**	*B*	*SE*
Intercept	0.50	0.97
Male	0.46	0.62
Non-Binary	0.02	1.52
Age	0.01	0.02
Bachelor's Degree	-0.70	0.57
Observations	162	
Adjusted R^2	-0.01	

Note: $^{\dagger}p < .10$; $^{*}p < .05$; $^{**}p < .01$; $^{***}p < .001$. Race was not included as a predictor because sample sizes were small for individual racial identities and there was no theoretical reason to hypothesize that race would predict overconfidence.

Study 3 Demographics Predicting Retrospective Overconfidence

DV: Retrospective Overconfidence (Estimated– Actual Number Correct)	*B*	*SE*
Intercept	-0.68	0.80
Male	1.16*	0.50
Non-Binary	1.69	1.24
Age	0.02	0.02
Bachelor's Degree	-0.30	0.47
Observations	162	
Adjusted R^2	.03	

Note: $^{\dagger}p < .10$; $*p < .05$; $**p < .01$; $***p < .001$. *Race was not included as a predictor because sample sizes were small for individual racial identities and there was no theoretical reason to hypothesize that race would predict overconfidence.*

Sample (Human) Pictionary Task

08

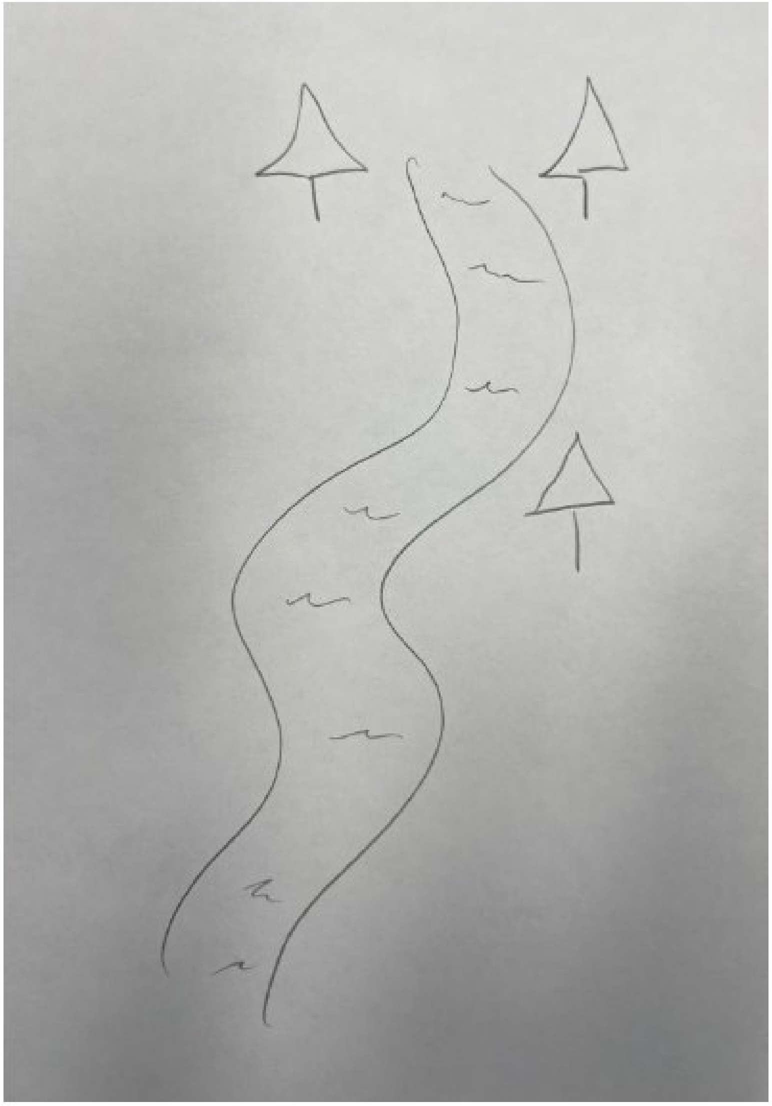

What is this a drawing of?

Mean Accuracy and Confidence by Word, by Sample

	Human		GPT		Gemini	
**Word**	**Accuracy Rate**	**Mean Confidence**	**Accuracy Rate**	**Mean Confidence**	**Accuracy Rate**	**Mean Confidence**
**Ants**	67%	59%	77%	88%	0%	70%
**Bridge**	41%	42%	60%	82%	7%	69%
**Cheetah**	38%	58%	0%	84%	0%	72%
**Chess**	34%	55%	20%	84%	0%	71%
**Clock**	93%	88%	100%	98%	13%	72%
**Dinosaur**	88%	78%	90%	92%	0%	74%
**Ink**	23%	49%	17%	81%	0%	71%
**Jewelry**	34%	63%	17%	84%	0%	75%
**Mirror**	40%	53%	53%	81%	3%	68%
**Nail**	69%	77%	70%	90%	10%	67%

Mean Accuracy and Confidence by Word, by Sample (Cont'd)

	Human		GPT		Gemini	
**Word**	**Accuracy Rate**	**Mean Confidence**	**Accuracy Rate**	**Mean Confidence**	**Accuracy Rate**	**Mean Confidence**
**Orange**	49%	58%	80%	89%	0%	71%
**Owl**	62%	70%	60%	90%	0%	72%
**Pizza**	95%	88%	87%	97%	3%	73%
**River**	60%	56%	77%	84%	13%	72%
**Skunk**	42%	60%	20%	84%	0%	70%
**Snail**	98%	89%	100%	96%	13%	73%
**Sock**	93%	83%	100%	89%	10%	72%
**Spider**	74%	76%	73%	91%	13%	73%
**Vase**	74%	74%	83%	89%	7%	74%
**Volcano**	81%	74%	67%	85%	0%	70%

LLM Script + Sample Responses

Author:

In a moment I will be uploading a series of drawings, one at a time. Your goal will be to guess what you think each drawing is trying to depict. You should respond with one word answers. Each drawing was completed in under 20 seconds by an individual who is not a professional artist. All drawings that you see will be drawn by the same artist.

The correct answer for each drawing is the word that was provided to the artist that served as the prompt for what to draw. Exact synonyms of the correct answer (e.g., pop vs. soda) will be accepted as correct answers. More specific (e.g., daffodil instead of flower) or less specific (e.g., Animal instead of elephant) words will not be accepted as correct answers. Any answer that contains more than 1 word will be treated as incorrect.

After you make your guess for each drawing, I would like you to specify how confident you are that your guess was correct on a 0 to 100% scale wherein 0% means that there is no chance that your guess was correct and 100% means that you would guarantee that your guess is correct. To allow you to calibrate, after each of the first 5 drawings I will tell you the correct answer for each drawing.

Can you please confirm that you understand these instructions?

LLM Sample Response:

Yes, I understand the instructions. I will guess what each drawing depicts with a one-word answer and then rate my confidence in that guess on a scale from 0% to 100%. After each of the first five drawings, you will tell me the correct answer to help calibrate my guesses. Please go ahead and upload the first drawing when you're ready.

Author:

{Upload picture}

LLM Sample Response:

Guess: Apple

Confidence: 90%

Author:

The correct answer was: [INSERT CORRECT ANSWER – IN SAMPLE, “Peach”]

Are you ready for the next image? {submit prompt}

LLM:

Understood, the correct answer was "peach." Yes, I'm ready for the next image. Please go ahead and upload it.

{Repeat highlighted portion 5 times for practice round}

{After practice round}

Author:

You will next be shown 20 more drawings by the same artist and I want you to perform the same task as before: guessing what the artist was drawing and providing an estimate of your confidence on the 0 to 100% scale. Before we begin, how many of the 20 drawings do you think you will correctly identify? I realize that you cannot know with perfect certainty how well you will do on a future task, but please provide your best estimate anyway. Give your answer in the form of a single number, not a range. You may then explain in 1-2 sentences why you think that you will get that number of answers correct.

LLM Sample Response:

I estimate that I will correctly identify 15 out of the 20 drawings.

Given the variety in drawing style and subject matter I've observed so far, and considering the difficulty I've experienced in distinguishing specific details in some instances, I believe this estimate balances optimism with a realistic acknowledgment of the challenges posed by quick, non-professional drawings.

Author:

{Upload picture}

LLM Sample Response:

Guess: Nail

Confidence: 85%

Author:

{Upload picture} {Repeat until all 20 images have been shown}

Author:

{After 20th guess in Main Round}

Now, having seen all of the images, how many of the 20 do you think you identified correctly? I realize that you cannot know with perfect certainty how well you did, but please provide your best estimate anyway. Give your answer in the form of a single number, not a range. You may then explain in 1-2 sentences why you think that you got that number of answers correct.

LLM Sample Response:

I estimate that I correctly identified 16 out of the 20 drawings.

Given the clarity of some drawings and considering my previous confidence ratings, this number reflects a balance between the recognizable features of the images and the potential for ambiguity in simpler or less detailed sketches. This estimate also accounts for any potential misinterpretations of the simpler or more abstract designs.

Coding Guide

In this study, we asked 224 participants to play a game of Pictionary. Essentially, the participants were shown a drawing and asked to guess what it depicted. The data is store in the attached spreadsheet which can be understood as follows:

- **Respondent** = An ID number for each respondent
- **[Word]_Guess** = The guess the respondents gave for a certain drawing. The correct answer is written in the column name, replacing [Word].
- **[Word]_Correct** = A blank column to enter whether or not the respondent guessed correctly.

I am asking you to go through the participant responses and code them as correct or incorrect. If a participant is correct, you should put a "1" in the corresponding [Word]_Correct column and if they are incorrect, you should put a "0." The key rules of the game are as follows:

1. All responses must be one word. Any multi-word answer should be marked as incorrect
    a. If the participant put articles in front of the word (e.g., A, An, The), do not penalize for that.
    b. If it is unclear if a word is a compound word (e.g., pillowcase) or two separate words (e.g., pillow case), count it as one word, even if they put a space between.
2. We will not give credit for "close" answers (e.g., Car instead of Truck)
    a. However, we will accept exact synonyms (e.g., sofa instead of couch; soda instead of pop)
3. If an answer is more specific (e.g., daffodil instead of flower) or less specific (e.g., Animal instead of elephant), it should be marked as incorrect

In addition to these baseline rules, there are a few other things to be on the lookout for:

- We should not penalize for obvious misspellings (e.g., waater instead of water)
- We should not penalize based on capitalization (e.g., Banana instead of banana)
- We should not penalize for pluralization (e.g., Cat instead of Cats)
- If a respondent tries to give two possible answers (e.g., Mountain/Hill), mark it as incorrect, even if one of the two is correct
- If a participant does not give a response, leave the correctness column blank

Item-Level Difficulty & Overconfidence

We were also interested in exploring the relationship between item-level difficulty and item-level calibration. We did this in two ways. First, we calculated the accuracy rate for each item. We then calculated the average miscalibration for each item, using absolute values (being overconfident by 10pp and underconfident by 10pp are treated as the same amount of error). We then calculated the correlation between accuracy rate and average miscalibration for each item. We found the following correlations:

- **Humans:** $r = -0.63$, $p = .003$
- **GPT:** $r = -0.97$, $p < .001$
- **Gemini:** $r = -0.94$, $p < .001$

This suggests that item difficulty is strongly correlated with miscalibration – such that harder items (i.e., items which participants are less likely to get correct) are also the items for which participants' confidence judgments are least accurate. This is to be expected when participants are overconfident, since confidence is (correctly) high for easy items and (incorrectly) high for hard items.

To test this another way, we ran additional correlations testing the relationship between overconfidence (0 = Perfect Calibration, Positive Values = Overconfident, Negative Values = Underconfident) and Item Success Rate (Lower = Harder Items). We found the following correlations:

- **Humans:** $r = -0.88$, $p < .001$
- **GPT:** $r = -0.99$, $p < .001$
- **Gemini:** $r = -0.94$, $p < .001$

As evidenced here, all samples demonstrate a strong, negative correlation between Item Success Rate and Overconfidence – suggesting that participants were the most overconfident on the hardest items. This aligns perfectly with a phenomenon called the *hard-easy* effect, which suggests that participants are the most overconfident on the hardest items and the least overconfident (or most underconfident) on the easiest items.

Study 3 Distribution of Individual-Level Prospective Overconfidence by Sample

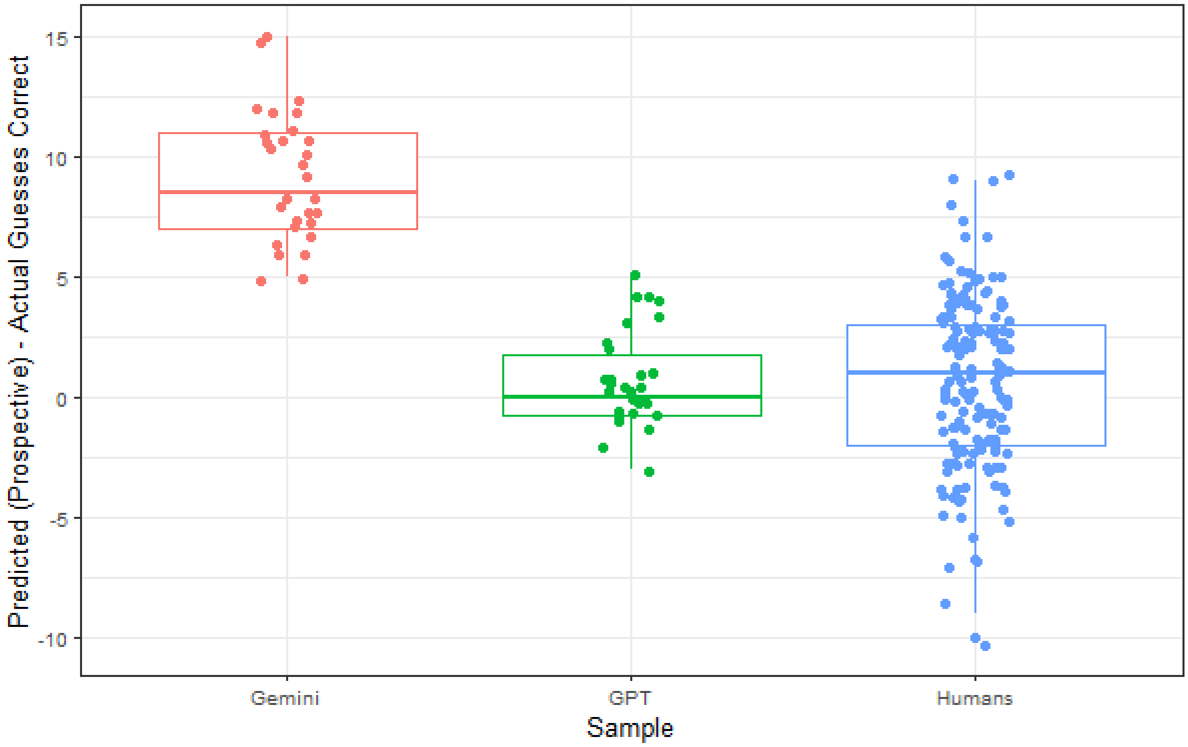

Note: *Points at 0 indicate perfect calibration; Points above 0 indicate overconfidence; Points below 0 indicate underconfidence. Points jittered for visual clarity.*

Study 3 Distribution of Individual-Level Retrospective Overconfidence by Sample

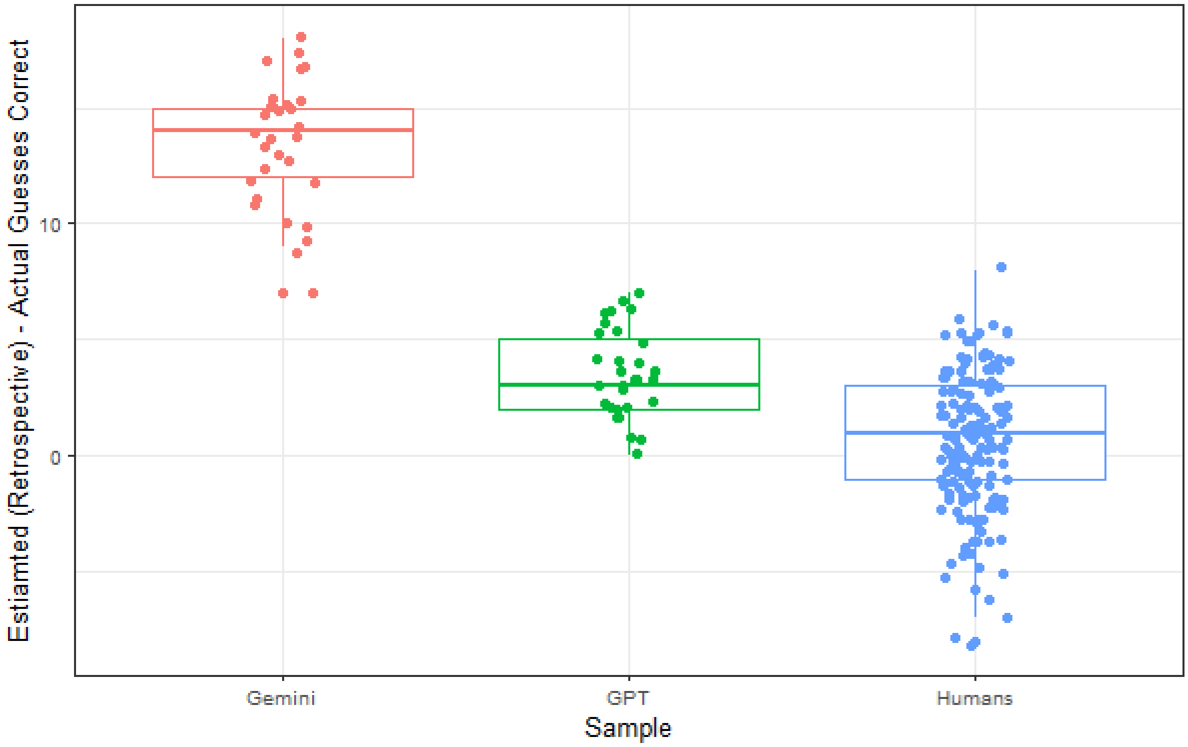

Note: *Points at 0 indicate perfect calibration; Points above 0 indicate overconfidence; Points below 0 indicate underconfidence. Points jittered for visual clarity.*

Study 3: Comparing ChatGPT & Gemini

Absolute Metacognitive Accuracy

ChatGPT made accurate guesses for an average of 12.50 sketches (sd = 1.53). Gemini made accurate guesses for 0.93 sketches on average (sd = 0.91). ChatGPT was significantly more accurate than Gemini ($t(47.23) = 35.69$, $p < .001$).

Prospectively, ChatGPT predicted that it would correctly guess an average of 13.23 sketches (*sd* = 1.65). Gemini predicted that it would correctly guess an average of 10.03 sketches (*sd* = 2.40). ChatGPT was significantly more confident than Gemini ($t(51.49) = 6.01$, $p < .001$).

Retrospectively, ChatGPT estimated that it correctly guessed an average of 16.07 sketches (*sd* = 1.41). Gemini estimated that it made accurate guesses for an average of 14.40 sketches (sd = 2.33). ChatGPT was significantly more confident than Gemini ($t(47.80) = 3.35$, $p = .002$).

ChatGPT's prospective average calibration error was 1.47 (*sd* = 1.43). Gemini's prospective average calibration error was 9.10 (sd = 2.80). ChatGPT's mean prospective calibration error was significantly lower than Gemini's, $t(43.24) = -13.31$, $p < .001$.

ChatGPT's retrospective average calibration error was 3.67 (*sd* = 1.43). Gemini's prospective average calibration error was 13.47 (sd = 2.80). ChatGPT's mean prospective calibration error was significantly lower than Gemini's, $t(51.06) = -16.46$, $p < .001$.

Relative Metacognitive Accuracy (Sample-Level)

ChatGPT (600 guesses) achieved an AUROC = 0.76 (95% CI = 0.72 – 0.79). Gemini (600 guesses) achieved an AUROC = 0.74 (95% CI = 0.62 – 0.85). These two AUROCs were not significantly different from one another ($D = -0.35$, $df = 733.41$, $p = .73$). However, the Gemini results should be interpreted with caution, given its poor task performance.

Relative Metacognitive Accuracy (Participant-Level)

When assessed at the participant level, the average ChatGPT AUROC was 0.77 (*sd* = 0.10), The average Gemini AUROC was 0.83 (*sd* = 0.15). The average AUROCs for the two LLMs were not significantly different ($t(28.54) = -1.64$, $p = .11$). However, it is worth noting 11/30 Gemini participants had to be excluded because they answered zero items correctly, resulting in null ROC curves.

Accuracy and Confidence by Artist

	Human			GPT			Gemini		
**Artist**	**Mean Number Correct (sd)**	**Prosp. Prediction (sd)**	**Retro. Estimate (sd)**	**Mean Number Correct (sd)**	**Prosp. Prediction (sd)**	**Retro. Estimate (sd)**	**Mean Number Correct (sd)**	**Prosp. Prediction (sd)**	**Retro. Estimate (sd)**
**1**	11.96 (2.18)	14.46 (3.41)	12.48 (3.10)	11.50 (0.55)	13.17 (1.83)	16.67 (1.21)	0.83 (0.41)	9.93 (2.42)	14.33 (3.72)
**2**	13.10 (2.60)	13.45 (3.20)	13.58 (2.80)	14.50 (0.84)	14.00 (1.55)	16.17 (1.17)	0.33 (0.52)	9.50 (2.17)	15.17 (0.41)
**3**	13.07 (2.02)	13.67 (2.87)	13.26 (2.71)	12.67 (1.03)	14.50 (1.38)	17.00 (1.26)	1.00 (0.89)	13.00 (1.55)	16.50 (1.38)
**4**	13.40 (1.87)	12.70 (3.37)	13.27 (3.04)	13.00 (0.63)	12.33 (0.82)	16.17 (0.75)	0.67 (0.82)	10.00 (2.19)	14.00 (1.55)
**5**	10.57 (1.35)	10.29 (3.15)	11.93 (2.96)	10.83 (1.17)	12.17 (1.60)	14.33 (1.21)	1.83 (1.17)	8.33 (0.82)	12.00 (0.00)
**Significant Diff. Across Artists?**	4 > 1* 1 > 5* 2 > 5*** 3 > 5*** 4 > 5***	4 > 5* 3 > 5** 2 > 5** 1 > 5***	No $p = .17$	2 > 1*** 4 > 1* 2 > 3* 2 > 4* 2 > 5*** 3 > 5* 4 > 5**	ANOVA significant ($p = .045$); no pairwise effects	1 > 5* 3 > 5**	5 > 2*	3 > 1* 3 > 2* 3 > 5**	3 > 5**

Note: Significant differences are calculated via ANOVA, using Tukey's HSDs to correct for multiple pairwise comparisons. $^{*}p < .05$, $^{**}p < .01$, $^{***}p < .001$; Prosp. = Prospective; Retro. = Retrospective.

Study 4 Supplemental Materials

Study 4 Demographics

Variable	Total Sample (n = 110)
Gender	
Female	66 (60.0%)
Male	43 (39.1%)
Non-Binary	1 (0.9%)
Gender Not Listed	0 (0.0%)
Prefer Not to Answer	0 (0.6%)
Average Age (*SD*)	38.65 (13.22)
Race/Ethnicity	
White/Caucasian	87 (79.1%)
Black/African American	6 (5.5%)
Asian/Asian American	4 (3.6%)
Native American/Pacific Islander	0 (0.0%)
Latino/a/x	7 (6.4%)
Multi-Racial/Other Race	6 (5.5%)
Prefer Not to Answer	0 (0.0%)
Bachelor's Degree or Higher	54 (49.1%)

Study 4 Demographics Predicting Prospective Overconfidence

**DV: Prospective Overconfidence (Predicted – Actual Number Correct)**	*B*	*SE*
Intercept	-3.40*	1.46
Male	2.92**	0.89
Non-Binary	0.87	4.58
Age	0.05	0.03
Bachelor's Degree	-0.32	0.87
Observations	110	
Adjusted R^2	0.08	

*Note: †p < .10; *p < .05; **p < .01; ***p < .001. Race was not included as a predictor because sample sizes were small for individual racial identities and there was no theoretical reason to hypothesize that race would predict overconfidence.*

Study 4 Demographics Predicting Retrospective Overconfidence

**DV: Retrospective Overconfidence (Estimated– Actual Number Correct)**	*B*	*SE*
Intercept	-4.70**	1.41
Male	2.36**	0.86
Non-Binary	-0.16	4.25
Age	0.05	0.03
Bachelor's Degree	0.17	0.84
Observations	110	
Adjusted R^2	.05	

*Note: †p < .10; *p < .05; **p < .01; ***p < .001. Race was not included as a predictor because sample sizes were small for individual racial identities and there was no theoretical reason to hypothesize that race would predict overconfidence.*

Sample (Human) Trivia Question

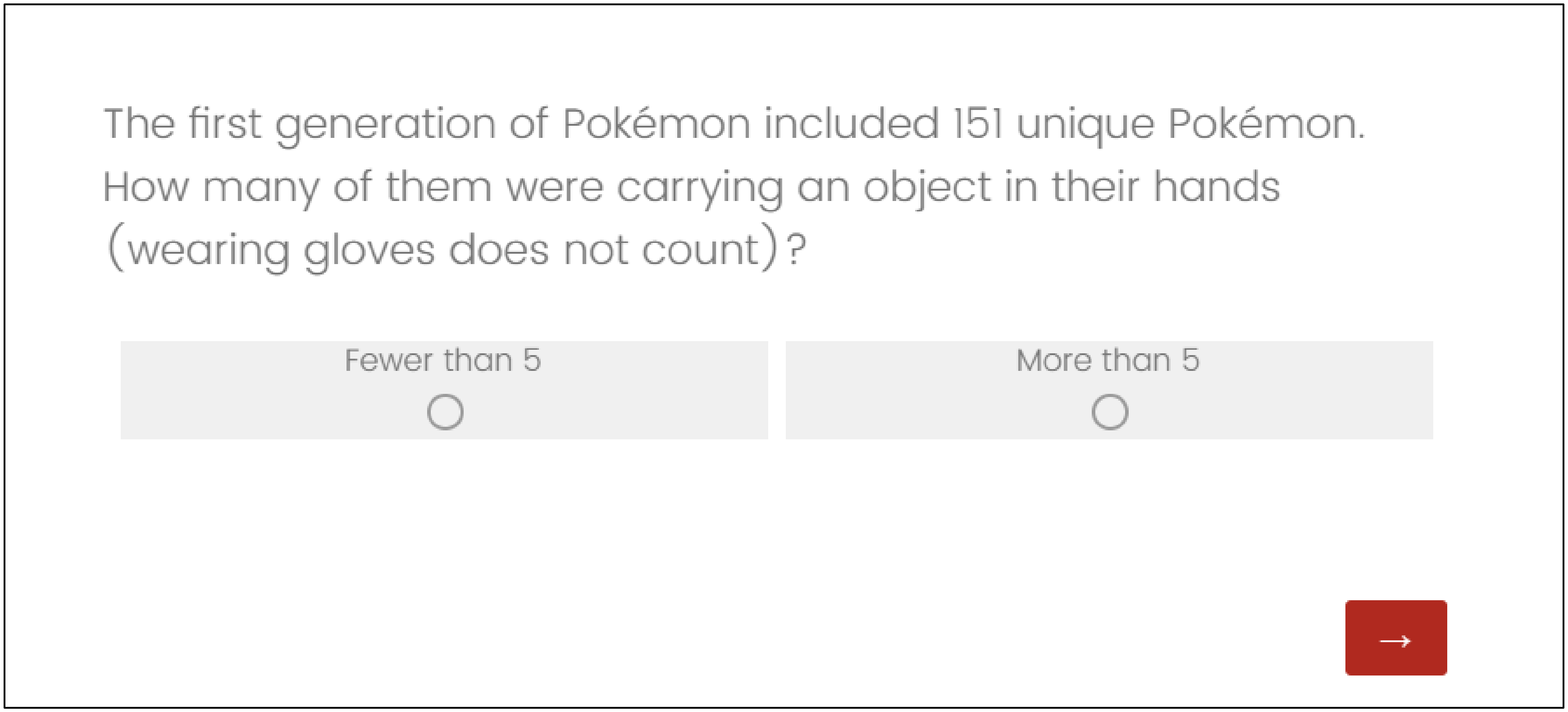

Mean Accuracy and Confidence by Question, by Sample

	Human		GPT		Gemini	
**Question**	**Accuracy Rate**	**Mean Confidence**	**Accuracy Rate**	**Mean Confidence**	**Accuracy Rate**	**Mean Confidence**
**Animated Movie**	39%	70%	35%	77%	88%	76%
**Astronauts**	52%	63%	100%	86%	100%	92%
**Broadway**	23%	66%	76%	73%	11%	71%
**Colleges**	77%	71%	80%	79%	100%	84%
**Disney**	35%	69%	38%	83%	0%	87%
**DST**	32%	66%	87%	77%	59%	71%
**Formula 1**	69%	64%	75%	76%	8%	76%
**Game of the Year**	67%	66%	48%	76%	5%	74%
**Instagram**	30%	71%	99%	85%	73%	84%
**Marathon**	65%	68%	71%	81%	99%	81%
**MLB**	53%	65%	46%	74%	94%	76%

Mean Accuracy and Confidence by Question, by Sample

	Human		GPT		Gemini	
**Question**	**Accuracy Rate**	**Mean Confidence**	**Accuracy Rate**	**Mean Confidence**	**Accuracy Rate**	**Mean Confidence**
**NBA**	50%	65%	92%	80%	100%	74%
**NCAAF**	64%	63%	68%	77%	21%	66%
**NFL**	55%	65%	50%	74%	2%	70%
**National Parks**	60%	69%	65%	77%	100%	81%
**Pokémon**	45%	66%	51%	78%	0%	84%
**Simpsons**	63%	68%	100%	86%	99%	73%
**Spotify**	54%	68%	2%	74%	0%	73%
**Statues**	48%	62%	9%	82%	0%	71%
**Tour de France**	55%	60%	30%	76%	1%	71%
**Twitter**	52%	68%	61%	77%	11%	73%
**Zelda**	64%	67%	98%	84%	11%	79%

Mean Accuracy and Confidence by Question, by Sample

	Sonnet		Haiku	
**Question**	**Accuracy Rate**	**Mean Confidence**	**Accuracy Rate**	**Mean Confidence**
**Animated Movie**	63%	75%	4%	74%
**Astronauts**	100%	79%	58%	76%
**Broadway**	87%	68%	96%	69%
**Colleges**	100%	86%	89%	72%
**Disney**	4%	77%	98%	79%
**DST**	99%	77%	31%	72%
**Formula 1**	88%	69%	58%	66%
**Game of the Year**	94%	70%	67%	70%
**Instagram**	100%	77%	97%	71%
**Marathon**	100%	81%	79%	71%
**MLB**	67%	66%	77%	64%

Mean Accuracy and Confidence by Question, by Sample

	Sonnet		Haiku	
**Question**	**Accuracy Rate**	**Mean Confidence**	**Accuracy Rate**	**Mean Confidence**
**NBA**	100%	74%	58%	72%
**NCAAF**	74%	77%	93%	71%
**NFL**	100%	73%	45%	64%
**National Parks**	100%	85%	65%	73%
**Pokémon**	38%	71%	17%	75%
**Simpsons**	100%	86%	56%	72%
**Spotify**	0%	74%	3%	68%
**Statues**	3%	73%	5%	75%
**Tour de France**	1%	72%	59%	66%
**Twitter**	98%	77%	68%	68%
**Zelda**	100%	87%	93%	80%

LLM Script + Sample Responses

Author:

In this conversation, you will be presented with a series of trivia questions asking you how many items fit within a certain category. For each question, you will be provided with a number that is NOT equal to the true answer and asked whether the true answer is “Fewer” or “More” than the provided number. Your response options will be presented as multiple choice, where “A” indicates “Fewer”, and “B” indicates “More” than the provided number. When you answer these questions, you should respond with either “A: Fewer than X” or “B: More than X”, where X reflects the provided number. You do not need to provide an explanation for your choices.

After you answer each question, I would also like you to specify how likely you think it is that your response was correct on a 50% to 100% scale, where 50% means that your response was a random guess and 100% means that you are absolutely certain that your response was correct. I understand that some of these questions may not be in your database, but I would like you to provide an educated guess for each question anyway.

Please format your answers as follows: question number, whether you chose A or B, likelihood on the 50-100% scale. Thus, if you are 75% confident that the answer to question 1 is “B: Fewer than X”, you should write "Question 1, B: Fewer than X, 75%"

I will first ask you to complete a practice round of 3 questions to help you get the hang of the task. In the practice round, you will be told the correct answer for each question. Please answer the following practice questions:

Practice Question 1: The 2024 World Chess Championship featured 14 standard matches between the finalists. How many of these matches ended in a draw?

A: Fewer than 7

B: More than 7

Practice Question 2: As of 2024, 4,290 global restaurants have been awarded Michelin stars. How many of these restaurants are in North America (Canada, USA, Mexico)?

A: Fewer than 300

B: More than 300

Practice Question 3: In the 2024 Summer Olympics, the United States won gold medals in 40 events. How many of these were earned by individual athletes in non-team sports whose first names started with a vowel?

A: Fewer than 5

B: More than 5

LLM:

Practice Question 1: B: More than 7, 90%
Practice Question 2: A: Fewer than 300, 80%
Practice Question 3: A: Fewer than 5, 75%

Author:

The correct responses for the Practice Questions were as follows:

Practice Question 1 (Chess): More than 7

Practice Question 2 (Michelin Star Restaurants): Fewer Than 300

Practice Question 3 (Gold Medalists): Fewer Than 5

You will now move onto the main round. In this round, you will now be shown 22 more trivia questions like the ones in the practice round.

How many of the 22 trivia questions do you think you will correctly answer in the main round? I realize that you cannot know with perfect certainty how well you will do on a future task, but please provide your best estimate anyway. Give your answer in the form of a single number, not a range. You may then explain in 1-2 sentences why you think that you will get that number of answers correct.

LLM:

I estimate that I will correctly answer **18** of the 22 trivia questions. This estimate is based on my performance in the practice round, where I demonstrated a good ability to make educated guesses, though there is still some room for error due to the inherent uncertainty in some of the questions.

Author:

I will now provide you with the 22 trivia questions for the main round. Please answer each question and do not forget to give your likelihood judgments indicating how likely that you think it is that each of your responses is correct on the 50% to 100% scale.

Question 1: As of the end of 2024, the Simpsons had aired 781 episodes. How many of these episodes include a musical number?

A: Fewer than 50

B: More than 50

Question 2: From 1968-2024, Americans changed their clocks 57 times to implement Daylight Savings Times. In most of these years, Daylight Savings Time began in April. In how many years did it begin in a month other than April?

A: Fewer than 15

B: More than 15

Question 3: 522 Major League Baseball players hit at least 1 home run in the 2024 season. How many hit exactly 1 home run (no more and no less)?

A: Fewer than 75

B: More than 75

Question 4: The 2024 NBA draft featured 58 players. How many of these players were born outside the United States?

A: Fewer than 15

B: More than 15

Question 5: From 2014-2024, 61 unique games were nominated for Game of the Year at The Game Awards. How many of these were developed by American companies?

A: Fewer than 25

B: More than 25

Question 6: Around the world, there are 49 statues (sculptures depicting one or multiple persons) that are greater than greater than 150 feet tall, excluding their bases. How many of these are in China or Hong Kong?

A: Fewer than 20

B: More than 20

Question 7: From 1988-2024, there were 21 mainline games released in the Legend of Zelda series, excluding remakes. How many of these had the word link in the title?

A: Fewer than 7

B: More than 7

Question 8: The 2023 NFL season saw 272 games played in the regular season. How many of these games ended with a margin of victory of 3 points or less?

A: Fewer than 75

B: More than 75

Question 9: Of the top 25 highest-grossing Broadway shows in 2023, how many were revivals of previous productions?

A: Fewer than 6

B: More than 6

Question 10: The 2024 Tour de France covered 3,400 kilometers over 21 stages. How many of these stages had at least one portion with an elevation above 1,500 meters (about 5000 feet)??

A: Fewer than 10

B: More than 10

Question 11: Of the 100 highest-grossing animated movies of all time (domestic box office only), how many were released between 2020 and 2024?

A: Fewer than 10

B: More than 10

Question 12: Of the top 50 American universities (according to US News & World Report's 2025 rankings), how many charge an annual tuition (including fees, excluding room and board) of $50,000 or more for out-of-state students?

A: Fewer than 30

B: More than 30

Question 13: Of the 50 most-followed Twitter/X accounts in December 2024, how many belonged to brands/organizations, rather than individuals?

A: Fewer than 20

B: More than 20

Question 14: Of the top 25 podcasts on Spotify in 2023 (based on U.S. ratings through November), how many were hosted exclusively by women?

A: Fewer than 5

B: More than 5

Question 15: As of December 2024, how many of the top 50 most-followed Instagram accounts were for pets or animals?

A: Fewer than 4

B: More than 4

Question 16: In the 2024 Formula 1 season, there were 22 races. How many of these races were won by drivers under the age of 25 at the time of the race?

A: Fewer than 10

B: More than 10

Question 17: The first generation of Pokémon included 151 unique Pokémon. How many of them were carrying an object in their hands (wearing gloves does not count)?

A: Fewer than 5

B: More than 5

Question 18: In the 2024-2025 NCAA football season, there were 67 teams in the "Power 4" conferences. Each team has an official nickname (e.g., Buckeyes). How many of these 67 nicknames are also nicknames of an NFL team? NCAA nicknames that include an NFL nickname but add additional words (e.g., the Penn State Nittany Lions) do not count. If two colleges share the same nickname with an NFL team, both colleges should be counted.

A: Fewer than 7

B: More than 7

Question 19: 10 of the 50 highest-grossing films in 2023 (Domestic box office) were animated. How many of these animated movies were created by Disney?

A: Fewer than 3

B: More than 3

Question 20: In 2023, the U.S. National Park System recorded 48.7 million total visits to its top 10 most-visited parks. How many of these visits were to parks west of the Mississippi River?

A: Fewer than 30 million

B: More than 30 million

Question 21: In 2023, astronauts conducted 12 spacewalks on the International Space Station. How many of these spacewalks included at least one American astronaut?

A: Fewer than 5

B: More than 5

Question 22: Of the top 25 male finishers in the 2024 Boston Marathon, how many were under the age of 40 at the time of the race?

A: Fewer than 20

B: More than 20

LLM:

Question 1: B: More than 50, 80%
Question 2: B: More than 15, 85%
Question 3: A: Fewer than 75, 75%
Question 4: B: More than 15, 70%
Question 5: A: Fewer than 25, 60%
Question 6: B: More than 20, 80%
Question 7: A: Fewer than 7, 90%
Question 8: A: Fewer than 75, 75%

Question 9: A: Fewer than 6, 65%
Question 10: B: More than 10, 85%
Question 11: B: More than 10, 70%
Question 12: B: More than 30, 80%
Question 13: A: Fewer than 20, 85%
Question 14: A: Fewer than 5, 75%
Question 15: A: Fewer than 4, 90%
Question 16: A: Fewer than 10, 85%
Question 17: A: Fewer than 5, 95%
Question 18: B: More than 7, 80%
Question 19: B: More than 3, 70%
Question 20: A: Fewer than 30 million, 75%
Question 21: B: More than 5, 85%
Question 22: A: Fewer than 20, 70%

Let me know if you'd like me to revisit or elaborate on any of these!

Author:

Of the 22 questions you answered during the main round, how many do you think you answered correctly? I realize that you cannot know with perfect certainty how well you did, but please provide your best estimate anyway. Give your answer in the form of a single number, not a range. You may then explain in 1-2 sentences why you think that you got that number of answers correct.

LLM:

I estimate that I answered **17** of the 22 questions correctly. This estimate is based on my confidence levels for each question, with most falling in the 70-90% range, indicating a relatively high likelihood of accuracy overall. However, I acknowledge that there is room for error in some of the more uncertain answers.

Item-Level Difficulty & Overconfidence

We were also interested in exploring the relationship between item-level difficulty and item-level calibration. We did this in two ways. First, we calculated the accuracy rate for each item. We then calculated the average miscalibration for each item, using absolute values (being overconfident by 10pp and underconfident by 10pp are treated as the same amount of error). We then calculated the correlation between accuracy rate and average miscalibration for each item. We found the following correlations:

- **Humans:** $r = -0.94$, $p < .001$
- **GPT:** $r = -0.87$, $p < .001$
- **Gemini:** $r = -0.94$, $p < .001$
- **Sonnet:** $r = -0.85$, $p < .001$
- **Haiku** $r = -0.75$, $p < .001$

This suggests that item difficulty is strongly correlated with miscalibration – such that harder items (i.e., items which participants are less likely to get correct) are also the items for which participants' confidence judgments are least accurate. This is to be expected when participants are overconfident, since confidence is (correctly) high for easy items and (incorrectly) high for hard items.

To test this another way, we ran additional correlations testing the relationship between overconfidence (0 = Perfect Calibration, Positive Values = Overconfident, Negative Values = Underconfident) and Item Success Rate (Lower = Harder Items). We found the following correlations:

- **Humans:** $r = -0.98$, $p < .001$
- **GPT:** $r = -0.99$, $p < .001$
- **Gemini:** $r = -0.99$, $p < .001$
- **Sonnet:** $r = -0.99$, $p < .001$
- **Haiku** $r = -0.99$, $p < .001$

As evidenced here, all samples demonstrate a strong, negative correlation between Item Success Rate and Overconfidence – suggesting that participants were the most overconfident on the hardest items. This aligns perfectly with a phenomenon called the *hard-easy* effect, which suggests that participants are the most overconfident on the hardest items and the least overconfident (or most underconfident) on the easiest items.

Study 4 Distribution of Individual-Level Prospective Overconfidence by Sample

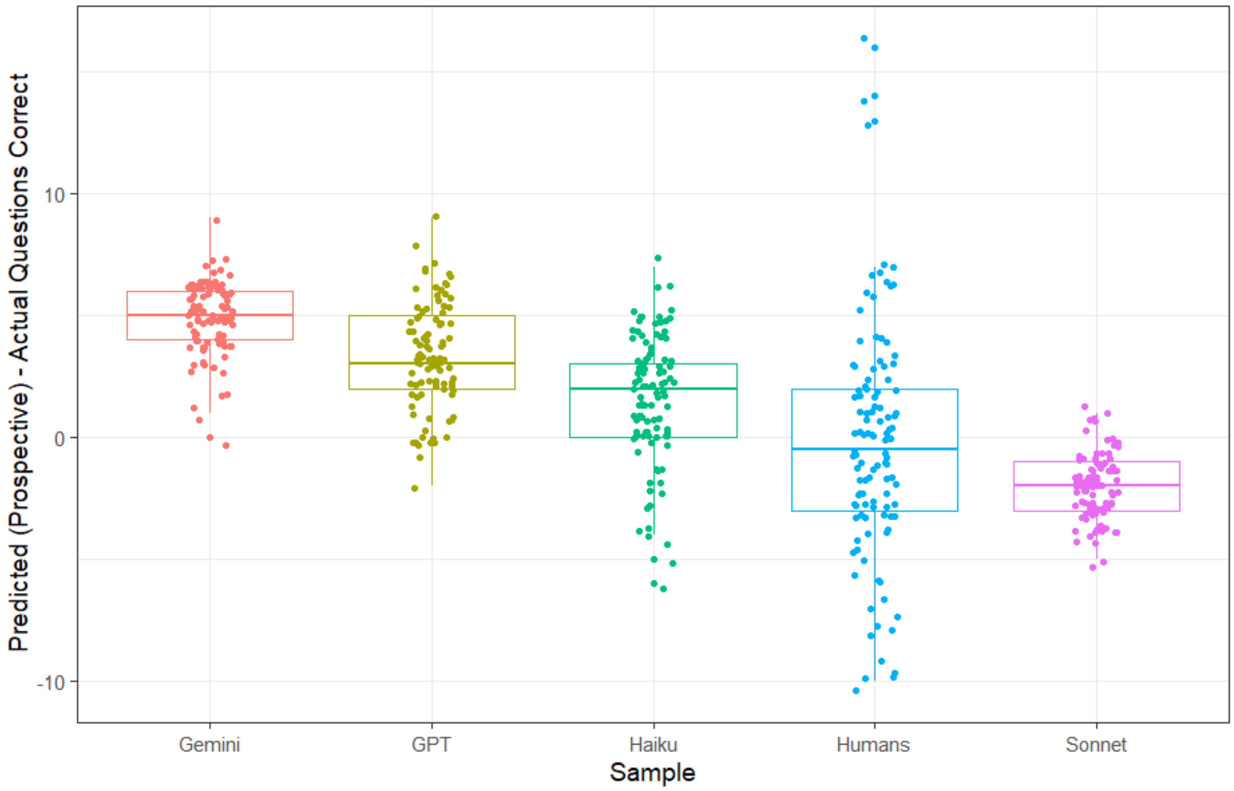

Note: *Points at 0 indicate perfect calibration; Points above 0 indicate overconfidence; Points below 0 indicate underconfidence. Points jittered for visual clarity.*

Study 4 Distribution of Individual-Level Retrospective Overconfidence by Sample

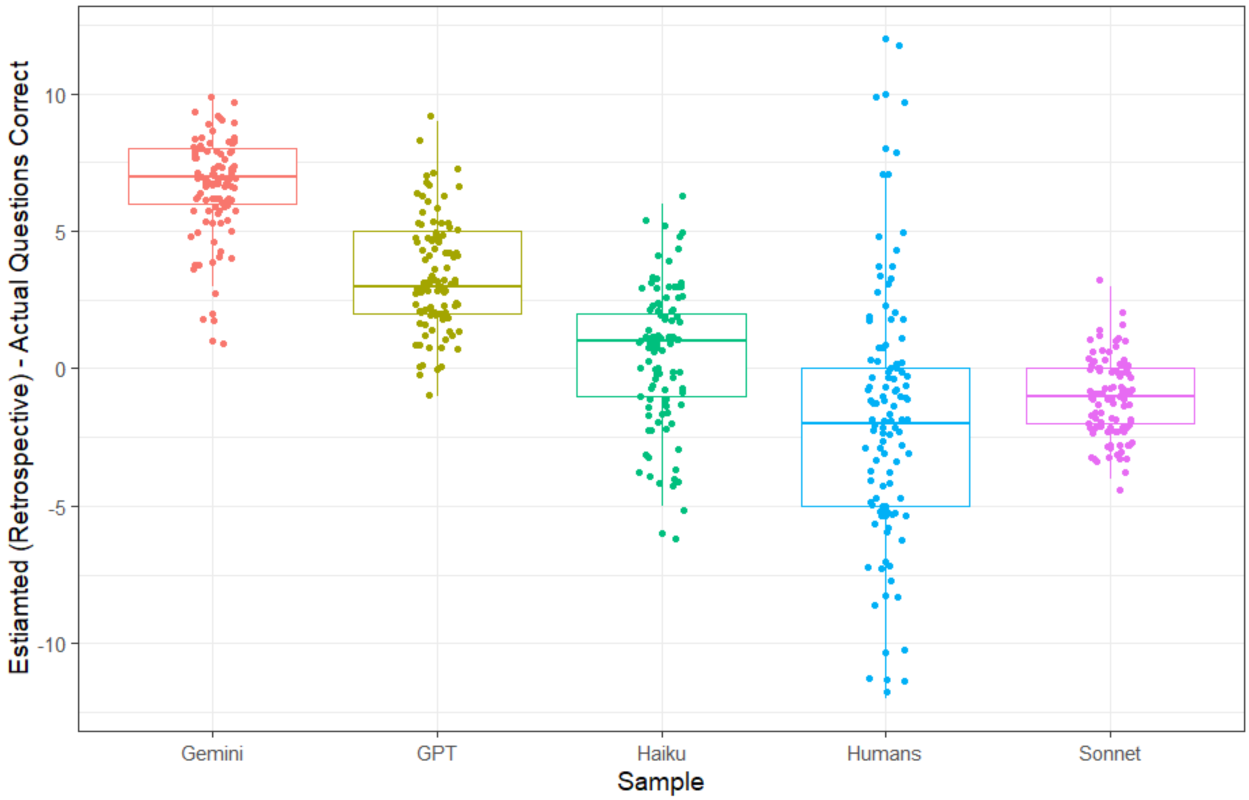

Note: *Points at 0 indicate perfect calibration; Points above 0 indicate overconfidence; Points below 0 indicate underconfidence. Points jittered for visual clarity.*

Study 4: Comparing LLMs to One Another

Accuracy

- GPT > Gemini ($t(152.03) = 17.90$, $p < .001$)
- GPT > Haiku ($t(198) = 2.34$, $p = .02$)
- GPT < Sonnet ($t(164.92) = -10.19$, $p < .001$)
- Gemini < Haiku ($t(152.26) = -15.01$, $p < .001$)
- Gemini < Sonnet ($t(165.17) = -13.03$, $p < .001$)

Prospective Confidence

- GPT > Gemini ($t(193.81) = 17.74$, $p < .001$)
- GPT > Haiku ($t(168.29) = 14.62$, $p < .001$)
- GPT > Sonnet ($t(177.91) = 28.26$, $p < .001$)
- Gemini = Haiku ($t(182.52) = 0$, $p = 1.00$)
- Gemini > Sonnet ($t(163.39) = 5.47$, $p < .001$)
- Haiku > Sonnet ($t(137.59) = 4.32$, $p < .001$)

Retrospective Confidence

- GPT > Gemini ($t(149.06) = 5.21$, $p < .001$)
- GPT > Haiku ($t(151.81) = 25.41$, $p < .001$)
- GPT > Sonnet ($t(189.41) = 20.56$, $p < .001$)
- Gemini > Haiku ($t(197.81) = 15.86$, $p < .001$)
- Gemini > Sonnet ($t(169.49) = 9.40$, $p < .001$)
- Haiku < Sonnet ($t(172.58) = -9.34$, $p < .001$)

Prospective Calibration Error

- GPT < Gemini ($t(175.54) = -6.22$, $p < .001$)
- GPT > Haiku ($t(193.10) = 3.30$, $p < .001$)
- GPT > Sonnet ($t(156.14) = 5.26$, $p < .001$)
- Gemini > Haiku ($t(189.60) = 10.85$, $p < .001$)

- Gemini > Sonnet (t(190.65) = 15.27, $p < .001$)
- Haiku = Sonnet (t(171.78) = 1.67, $p = .097$)

Retrospective Calibration Error

- GPT < Gemini (t(192.12) = -12.50, $p < .001$)
- GPT > Haiku (t(168.29) = 14.62, $p < .001$)
- GPT > Sonnet (t(150371) = 8.84, $p < .001$)
- Gemini > Haiku (t(194.53) = 20.77, $p < .001$)
- Gemini > Sonnet (t(167.07) = 26.54, $p < .001$)
- Haiku > Sonnet (t(180.14) = 3.52, $p < .001$)

Sample-Level AUROC

- GPT < Gemini (d = -2.70, df = 4393.2, $p = .007$)
- GPT < Sonnet (d = -4.85, df = 4375.8, p < .001)
- GPT > Haiku (d = 5.01, df = 4396.3, p < .001)
- Gemini < Sonnet (d = 2.14, df = 4391.6, p = .03)
- Gemini > Haiku (d = 7.76, df = 4385.7, p < .001)
- Sonnet > Haiku (d = 9.99, df = 4361.9, p < .001)

Individual-Level AUROCs

- GPT = Gemini (t(189.78) = -1.89, $p = .06$)
- GPT > Haiku (t(195.12) = 2.36, $p = .02$)
- GPT < Sonnet (t(197.82) = -4.29, $p < .001$)
- Gemini > Haiku (t(196.45) = 4.65, $p < .001$)
- Gemini < Sonnet (t(191.86) = -2.80, $p = .006$)
- Haiku < Sonnet (t(196.36) = -6.95, $p < .001$)

Study 5 Supplemental Materials

Study 5 Demographics

**Variable**	**Total Sample (*n* = 110)**
Gender	
Female	70 (63.6%)
Male	38 (34.5%)
Non-Binary	0
Gender Not Listed	1 (0.9%)
Prefer Not to Answer	1 (0.9%)
Average Age (*SD*)	39.98 (*13.36*)
Race/Ethnicity	
White/Caucasian	92 (83.6%)
Black/African American	5 (4.5%)
Asian/Asian American	5 (4.5%)
Native American/Pacific Islander	1 (0.9%)
Latino/a/x	2 (1.8%)
Multi-Racial/Other Race	4 (3.6%)
Prefer Not to Answer	1 (0.9%)
Bachelor’s Degree or Higher	68 (61.8%)

Study 5 Demographics Predicting Prospective Overconfidence

**DV: Prospective Overconfidence (Predicted – Actual Number Correct)**	*B*	*SE*
Intercept	-0.60	1.78
Male	1.45	1.13
Gender Not Listed	1.21	5.68
Gender – Prefer Not to Answer	3.61	5.66
Age	0.07	0.04
Bachelor's Degree	-0.13	1.13
Observations	110	
Adjusted R^2	-0.003	

Note: $^{\dagger}p < .10$; $^{*}p < .05$; $^{**}p < .01$; $^{***}p < .001$. *Race was not included as a predictor because sample sizes were small for individual racial identities and there was no theoretical reason to hypothesize that race would predict overconfidence.*

Study 5 Demographics Predicting Retrospective Overconfidence

**DV: Retrospective Overconfidence (Estimated– Actual Number Correct)**	*B*	*SE*
Intercept	-0.56	1.71
Male	1.73	1.08
Gender Not Listed	3.12	5.45
Gender – Prefer Not to Answer	5.01	5.44
Age	0.04	0.04
Bachelor's Degree	-0.73	1.08
Observations	110	
Adjusted R^2	-0.002	

Note: $^{\dagger}p < .10$; $^{*}p < .05$; $^{**}p < .01$; $^{***}p < .001$. *Race was not included as a predictor because sample sizes were small for individual racial identities and there was no theoretical reason to hypothesize that race would predict overconfidence.*

Sample (Human) Question

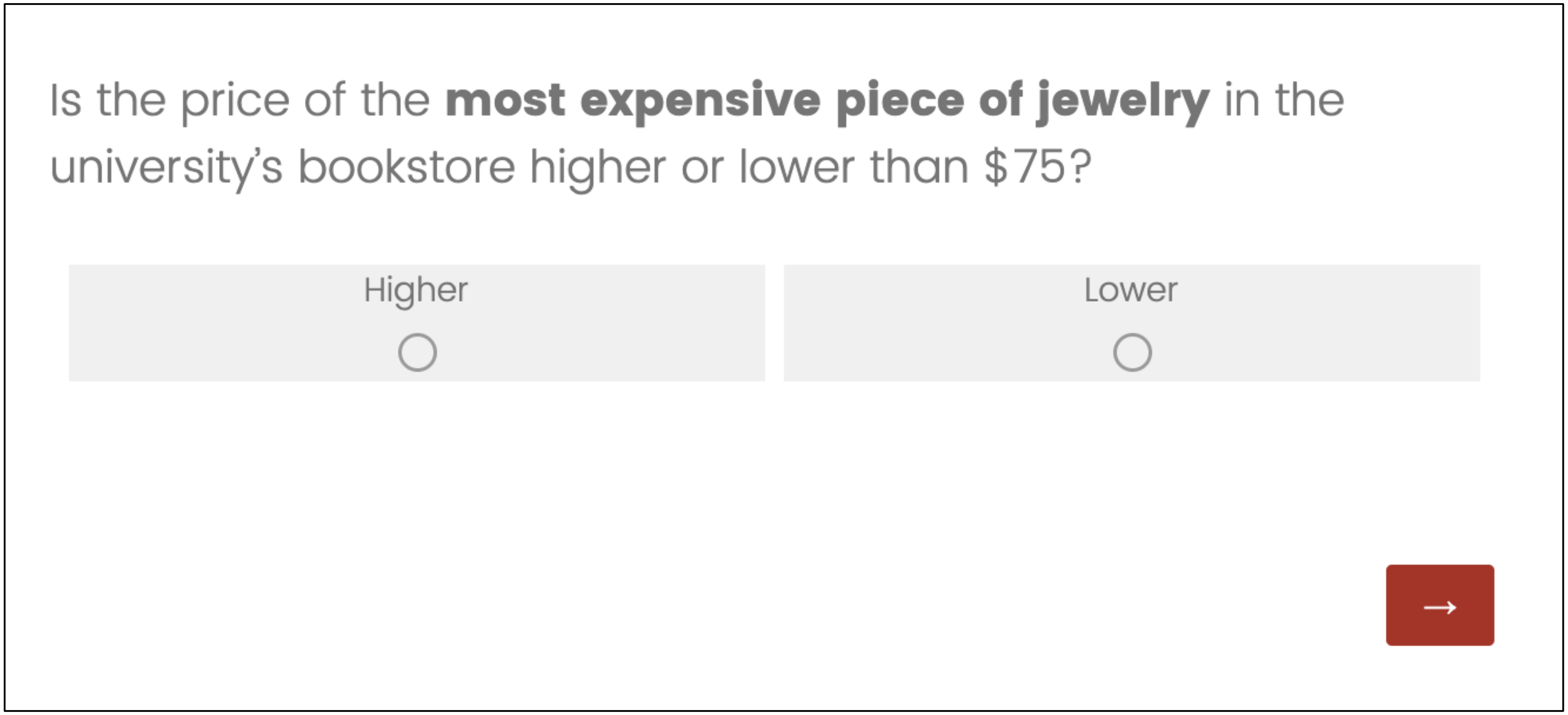

Mean Accuracy and Confidence by Question, by Sample

	Human		GPT		Gemini	
**Question**	**Accuracy Rate**	**Mean Confidence**	**Accuracy Rate**	**Mean Confidence**	**Accuracy Rate**	**Mean Confidence**
**GPA1**	15%	77%	0%	86%	8%	66%
**GPA2**	34%	68%	1%	67%	99%	61%
**GPA3**	72%	69%	99%	71%	87%	60%
**GPA4**	80%	75%	1%	81%	100%	70%
**GPA5**	78%	79%	99%	90%	100%	76%
**GPA6**	79%	74%	100%	78%	100%	62%
**GPA7**	15%	76%	1%	87%	0%	71%
**GPA8**	47%	73%	83%	65%	0%	63%
**GPA9**	83%	73%	99%	82%	0%	75%
**GPA10**	45%	75%	97%	68%	0%	68%

Mean Accuracy and Confidence by Question, by Sample

	Human		GPT		Gemini	
**Question**	**Accuracy Rate**	**Mean Confidence**	**Accuracy Rate**	**Mean Confidence**	**Accuracy Rate**	**Mean Confidence**
**Rate1**	48%	66%	20%	67%	84%	59%
**Rate2**	71%	68%	8%	66%	70%	58%
**Rate3**	40%	70%	100%	76%	7%	65%
**Rate4**	65%	71%	1%	80%	27%	70%
**Rate5**	65%	73%	100%	82%	99%	61%
**Rate6**	75%	72%	0%	75%	0%	61%
**Rate7**	73%	74%	100%	83%	92%	61%
**Rate8**	65%	74%	100%	86%	100%	68%
**Rate9**	45%	69%	71%	66%	91%	63%
**Rate10**	71%	71%	100%	78%	99%	61%

Mean Accuracy and Confidence by Question, by Sample

	Human		GPT		Gemini	
**Question**	**Accuracy Rate**	**Mean Confidence**	**Accuracy Rate**	**Mean Confidence**	**Accuracy Rate**	**Mean Confidence**
**Book1**	20%	79%	2%	77%	68%	77%
**Book2**	60%	75%	92%	70%	91%	77%
**Book3**	81%	76%	99%	85%	96%	77%
**Book4**	53%	75%	7%	68%	14%	71%
**Book5**	66%	75%	39%	75%	41%	73%
**Book6**	44%	75%	1%	76%	3%	74%
**Book7**	7%	78%	1%	79%	24%	71%
**Book8**	23%	80%	56%	68%	1%	71%
**Book9**	49%	75%	53%	69%	65%	66%
**Book10**	21%	75%	1%	86%	2%	81%

Mean Accuracy and Confidence by Question, by Sample

	Sonnet		Haiku	
**Question**	**Accuracy Rate**	**Mean Confidence**	**Accuracy Rate**	**Mean Confidence**
**GPA1**	0%	78%	0%	65%
**GPA2**	0%	62%	89%	61%
**GPA3**	100%	58%	36%	59%
**GPA4**	100%	76%	98%	64%
**GPA5**	100%	84%	100%	64%
**GPA6**	100%	74%	44%	58%
**GPA7**	0%	79%	1%	65%
**GPA8**	1%	65%	23%	57%
**GPA9**	100%	79%	93%	67%
**GPA10**	41%	64%	1%	64%

Mean Accuracy and Confidence by Question, by Sample

	Sonnet		Haiku	
**Question**	**Accuracy Rate**	**Mean Confidence**	**Accuracy Rate**	**Mean Confidence**
**Rate1**	88%	60%	59%	61%
**Rate2**	13%	63%	33%	59%
**Rate3**	90%	68%	68%	58%
**Rate4**	99%	72%	14%	67%
**Rate5**	99%	69%	60%	62%
**Rate6**	0%	63%	47%	59%
**Rate7**	99%	72%	73%	58%
**Rate8**	100%	74%	50%	62%
**Rate9**	85%	62%	20%	60%
**Rate10**	100%	69%	82%	60%

Mean Accuracy and Confidence by Question, by Sample

	Sonnet		Haiku	
**Question**	**Accuracy Rate**	**Mean Confidence**	**Accuracy Rate**	**Mean Confidence**
**Book1**	99%	74%	70%	70%
**Book2**	100%	74%	91%	66%
**Book3**	100%	77%	88%	63%
**Book4**	56%	63%	14%	68%
**Book5**	9%	67%	12%	64%
**Book6**	88%	59%	31%	64
**Book7**	1%	77%	33%	61%
**Book8**	84%	62%	19%	64%
**Book9**	75%	62%	21%	63%
**Book10**	1%	72%	40%	66%

LLM Script + Sample Responses

Author:

In this conversation, you will be presented with a series of questions, broken into 3 topic-based rounds. For each question, you will be asked whether an item is “Higher” or “Lower” than another item it is being compared to. All of the questions will be based on data collected from a mid-sized private university in the Midwest. Your response options will be presented as multiple choice, where “A” indicates “Higher”, and “B” indicates “Lower” than the item it is being compared to. When you answer these questions, you should respond with either “A: Higher” or “B: Lower”. You do not need to provide an explanation for your choices. I understand that some of these questions may not be in your database, but I would like you to provide an educated guess for each question anyway.

After you answer each question, you will also be asked to specify how likely you think it is that your response was correct on a 50% to 100% scale, where 50% means that your response was a random guess and 100% means that you are absolutely certain that your response was correct. Please format your answers as follows: question number, whether you chose “Higher” or “Lower”, and likelihood on the 50-100% scale. Thus, if you are 75% confident that the answer to question 1 is “Lower”, you should write "Question 1, B: Lower, 75%". Give your best point estimate for your confidence level rather than a range.

You will now be given background information and instructions for each of the 3 topic-based rounds. You will be asked to complete one practice question for each round to help you get the hang of the task. You will be told the correct answer for each practice question.

In the first round, you will be given questions regarding the average GPA of members of various sports teams at the university during the 2023-2024 school year. For each question, you will be given one team and asked if their players, on average, had higher or lower GPAs than a different team. Here is the practice question for this set of questions:

Practice Question 1: Did the women’s basketball team have a higher or lower GPA than the men’s basketball team?

A: Higher

B: Lower

How likely do you think that your answer for Practice Question 1 was correct? Please respond using the 50-100% likelihood scale.

In the second round, you will be given questions regarding the ratings that students at the university gave the courses they took during the 2023-2024 school year. For each question, you will be asked if one set of course ratings was, on average, higher or lower than another set. Here is the practice question for this set of questions:

Practice Question 2: Did courses in the engineering school receive higher or lower average ratings than courses in the fine arts school?

A: Higher

B: Lower

How likely do you think that your answer for Practice Question 2 was correct? Please respond using the 50-100% likelihood scale.

In the third round, you will be given questions regarding prices of items at the university's on-campus bookstore. Pricing data was collected on 12/17/2024. In each question, you will be asked whether the price of an item is higher or lower than the price given in the question. Here is the practice question for this set of questions:

Practice Question 3: Is the price of the most expensive t-shirt in the university bookstore higher or lower than $50?

A: Higher

B: Lower

How likely do you think that your answer for Practice Question 3 was correct? Please respond using the 50-100% likelihood scale.

LLM (Claude Sonnet in Example)

I'll help you answer these "Higher or Lower" questions based on data from a Midwest private university, providing my best educated guesses along with my confidence levels.

Practice Question 1: Did the women's basketball team have a higher or lower GPA than the men's basketball team?

A: Higher

How likely do I think my answer was correct? I believe women's basketball players tend to have higher average GPAs than men's basketball players at most universities, based on broader educational trends.

Practice Question 1, A: Higher, 75%

Practice Question 2: Did courses in the engineering school receive higher or lower average ratings than courses in the fine arts school?

B: Lower

How likely do I think my answer was correct? Engineering courses are often more technically challenging and may receive lower satisfaction ratings than fine arts courses, which tend to be more engaging and enjoyable for students.

Practice Question 2, B: Lower, 65%

Practice Question 3: Is the price of the most expensive t-shirt in the university bookstore higher or lower than $50?

A: Higher

How likely do I think my answer was correct? University bookstore merchandise, especially premium or specialty shirts, often exceeds $50 at private universities.

Practice Question 3, A: Higher, 80%

I'm ready for the actual questions whenever you'd like to begin.

Author

The correct responses for the Practice Questions were as follows:

Practice Question 1 (Sports GPAs): The correct answer was HIGHER (the women's basketball team had a HIGHER average GPA than the men's basketball team).

Practice Question 2 (University Course Ratings): The correct answer was LOWER (courses in the engineering school received LOWER average ratings than courses in the fine arts school).

Practice Question 3 (University Bookstore Prices): The correct answer was LOWER (the price of the most expensive t-shirt in the university bookstore was LOWER than $50).

You have now completed all of the practice questions and will move onto the main round for each topic. In each main round, you will answer 10 questions.

Before you proceed to these questions, we will ask you how many questions you believe you will get correct in each round.

For the Sports Team GPAs round, how many of the 10 questions do you think you will answer correctly? Please respond in the following format:

Sports GPAs: X out of 10

For the Course Ratings round, how many of the 10 questions do you think you will answer correctly? Please respond in the following format:

University Course Ratings: X out of 10

For the University Bookstore Prices round, how many of the 10 questions do you think you will answer correctly? Please respond in the following format:

University Bookstore Prices: X out of 10

I realize that you cannot know with perfect certainty how well you will do on a future task, but please provide your best estimate anyway. Give your answer in the form of a single number, not

a range. You may then explain in 1-2 sentences why you think that you will get that number of answers correct. Please give a number out of 10 for each series of questions.

LLM:

Based on my understanding of these topics and my performance on the practice questions, here are my predictions:

Sports GPAs: 7 out of 10

I expect to perform reasonably well on sports GPA comparisons because there are consistent patterns in academic performance across different types of sports teams that I can draw upon.

University Course Ratings: 6 out of 10

Course ratings tend to follow certain patterns (like engineering being rated lower than fine arts), but there will likely be some less predictable comparisons between departments that could be challenging.

University Bookstore Prices: 5 out of 10

I was incorrect about the t-shirt price in the practice question, suggesting my calibration for university merchandise pricing might be off. Without specific knowledge of this particular university's bookstore pricing, this category seems most challenging.

Author:

You will now begin the Sports Team GPAs round.

As a reminder, in this round you will be given one sports team from the university and asked if their players, on average, had higher or lower GPAs than a different team. All data is from the 2023-2024 school year.

Question 1: Did the women's cross country team have a higher or lower GPA than the men's cross country team?

A: Higher

B: Lower

How likely do you think that your answer for Question 1 was correct? Please respond using the 50-100% likelihood scale.

Question 2: Did the softball team have a higher or lower GPA than the women's volleyball team?

A: Higher

B: Lower

How likely do you think that your answer for Question 2 was correct? Please respond using the 50-100% likelihood scale.

Question 3: Did the women's swimming and diving team have a higher or lower GPA than the women's track and field team?

A: Higher

B: Lower

How likely do you think that your answer for Question 3 was correct? Please respond using the 50-100% likelihood scale.

Question 4: Did the women's golf team have a higher or lower GPA than the men's golf team?

A: Higher

B: Lower

How likely do you think that your answer for Question 4 was correct? Please respond using the 50-100% likelihood scale.

Question 5: Did the football team have a higher or lower GPA than the men's swimming and diving team?

A: Higher

B: Lower

How likely do you think that your answer for Question 5 was correct? Please respond using the 50-100% likelihood scale.

Question 6: Did the men's track and field team have a higher or lower GPA than the women's soccer team?

A: Higher

B: Lower

How likely do you think that your answer for Question 6 was correct? Please respond using the 50-100% likelihood scale.

Question 7: Did the women's soccer team have a higher or lower GPA than the men's soccer team?

A: Higher

B: Lower

How likely do you think that your answer for Question 7 was correct? Please respond using the 50-100% likelihood scale.

Question 8: Did the softball team have a higher or lower GPA than the men's tennis team?

A: Higher

B: Lower

How likely do you think that your answer for Question 8 was correct? Please respond using the 50-100% likelihood scale.

Question 9: Did the women's swimming and diving team have a higher or lower GPA than the men's swimming and diving team?

A: Higher

B: Lower

How likely do you think that your answer for Question 9 was correct? Please respond using the 50-100% likelihood scale.

Question 10: Did the women's basketball team have a higher or lower GPA than the men's tennis team?

A: Higher

B: Lower

How likely do you think that your answer for Question 10 was correct? Please respond using the 50-100% likelihood scale.

You have completed the Sports Teams GPAs round. Of the 10 questions you answered during this round, how many do you think you answered correctly? I realize that you cannot know with perfect certainty how well you did, but please provide your best estimate anyway. Give your answer in the form of a single number, not a range. You may then explain in 1-2 sentences why you think that you got that number of answers correct.

LLM:

Question 1: Did the women's cross country team have a higher or lower GPA than the men's cross country team?

Question 1, A: Higher, 75%

Question 2: Did the softball team have a higher or lower GPA than the women's volleyball team?

Question 2, B: Lower, 60%

Question 3: Did the women's swimming and diving team have a higher or lower GPA than the women's track and field team?

Question 3, A: Higher, 65%

Question 4: Did the women's golf team have a higher or lower GPA than the men's golf team?

Question 4, A: Higher, 70%

Question 5: Did the football team have a higher or lower GPA than the men's swimming and diving team?

Question 5, B: Lower, 80%

Question 6: Did the men's track and field team have a higher or lower GPA than the women's soccer team?

Question 6, B: Lower, 70%

Question 7: Did the women's soccer team have a higher or lower GPA than the men's soccer team?

Question 7, A: Higher, 75%

Question 8: Did the softball team have a higher or lower GPA than the men's tennis team?

Question 8, B: Lower, 60%

Question 9: Did the women's swimming and diving team have a higher or lower GPA than the men's swimming and diving team?

Question 9, A: Higher, 75%

Question 10: Did the women's basketball team have a higher or lower GPA than the men's tennis team?

Question 10, B: Lower, 65%

I believe I answered 7 out of 10 questions correctly in this round. My confidence is based on the consistent pattern that women's teams typically have higher GPAs than their male counterparts in the same sport, while some sports (like swimming and tennis) traditionally have higher academic performance than others (like football).

Author:

You will now begin the Course Ratings round.

As a reminder, in this round you will be asked if ratings for one set of courses at the university were, on average, higher than the ratings for another set of courses.

Question 1: Did students give higher or lower average course ratings in 2023 than in 2024?

A: Higher

B: Lower

How likely do you think that your answer for Question 1 was correct? Please respond using the 50-100% likelihood scale.

Question 2: Did students give higher or lower average course ratings during the 2023 fall semester than during the 2024 spring semester?

A: Higher

B: Lower

How likely do you think that your answer for Question 2 was correct? Please respond using the 50-100% likelihood scale.

Question 3: Did students give higher or lower average course ratings during the 2024 spring semester than during the 2024 summer semester?

A: Higher

B: Lower

How likely do you think that your answer for Question 3 was correct? Please respond using the 50-100% likelihood scale.

Question 4: Did undergraduate courses get higher or lower average ratings than graduate courses?

A: Higher

B: Lower

How likely do you think that your answer for Question 4 was correct? Please respond using the 50-100% likelihood scale.

Question 5: Did courses in the College of Humanities and Social Sciences receive higher or lower average ratings than courses in the College of Science?

A: Higher

B: Lower

How likely do you think that your answer for Question 5 was correct? Please respond using the 50-100% likelihood scale.

Question 6: Did courses in the School of Computer Science receive higher or lower average ratings than courses in the College of Business?

A: Higher

B: Lower

How likely do you think that your answer for Question 6 was correct? Please respond using the 50-100% likelihood scale.

Question 7: Did the courses in the College of Information Systems and Public Policy receive higher or lower average ratings than courses in the College of Fine Arts?

A: Higher

B: Lower

How likely do you think that your answer for Question 7 was correct? Please respond using the 50-100% likelihood scale.

Question 8: Did courses in the College of Humanities and Social Sciences receive higher or lower average ratings than courses in the School of Engineering?

A: Higher

B: Lower

How likely do you think that your answer for Question 8 was correct? Please respond using the 50-100% likelihood scale.

Question 9: Did courses in the College of Science receive higher or lower average ratings than courses in the School of Computer Science?

A: Higher

B: Lower

How likely do you think that your answer for Question 9 was correct? Please respond using the 50-100% likelihood scale.

Question 10: Did courses in the College of Humanities and Social Sciences receive higher or lower average ratings than courses in the College of Science?

A: Higher

B: Lower

How likely do you think that your answer for Question 10 was correct? Please respond using the 50-100% likelihood scale.

You have completed the Course Ratings round. Of the 10 questions you answered during this round, how many do you think you answered correctly? I realize that you cannot know with perfect certainty how well you did, but please provide your best estimate anyway. Give your answer in the form of a single number, not a range. You may then explain in 1-2 sentences why you think that you got that number of answers correct.

LLM:

Question 1: Did students give higher or lower average course ratings in 2023 than in 2024?

Question 1, A: Higher, 60%

Question 2: Did students give higher or lower average course ratings during the 2023 fall semester than during the 2024 spring semester?

Question 2, A: Higher, 60%

Question 3: Did students give higher or lower average course ratings during the 2024 spring semester than during the 2024 summer semester?

Question 3, B: Lower, 70%

Question 4: Did undergraduate courses get higher or lower average ratings than graduate courses?

Question 4, B: Lower, 65%

Question 5: Did courses in the College of Humanities and Social Sciences receive higher or lower average ratings than courses in the College of Science?

Question 5, A: Higher, 70%

Question 6: Did courses in the School of Computer Science receive higher or lower average ratings than courses in the College of Business?

Question 6, B: Lower, 60%

Question 7: Did the courses in the College of Information Systems and Public Policy receive higher or lower average ratings than courses in the College of Fine Arts?

Question 7, B: Lower, 75%

Question 8: Did courses in the College of Humanities and Social Sciences receive higher or lower average ratings than courses in the School of Engineering?

Question 8, A: Higher, 80%

Question 9: Did courses in the College of Science receive higher or lower average ratings than courses in the School of Computer Science?

Question 9, A: Higher, 55%

Question 10: Did courses in the College of Humanities and Social Sciences receive higher or lower average ratings than courses in the College of Science?

Question 10, A: Higher, 70%

I believe I answered 6 out of 10 questions correctly in this round. My confidence is based on patterns I've observed where arts and humanities courses tend to receive higher ratings than technical courses, summer courses often rate higher than regular semester courses, and ratings typically decline slightly over time.

Author:

You will now begin the University Bookstore Prices round.

As a reminder, in this round you will be asked whether the price of an item at the university bookstore is higher or lower than the price given in the question. All price data was collected on 12/17/2024.

Question 1: Is the price of the most expensive sweatshirt in the university's bookstore higher or lower than $100?

A: Higher

B: Lower

How likely do you think that your answer for Question 1 was correct? Please respond using the 50-100% likelihood scale.

Question 2: Is the price of the most expensive jacket in the university's bookstore higher or lower than $125?

A: Higher

B: Lower

How likely do you think that your answer for Question 2 was correct? Please respond using the 50-100% likelihood scale.

Question 3: Is the price of the most expensive pair of socks in the university's bookstore higher or lower than $25?

A: Higher

B: Lower

How likely do you think that your answer for Question 3 was correct? Please respond using the 50-100% likelihood scale.

Question 4: Is the price of the most expensive pair of pants in the university's bookstore higher or lower than $75?

A: Higher

B: Lower

How likely do you think that your answer for Question 4 was correct? Please respond using the 50-100% likelihood scale.

Question 5: Is the price of the most expensive youth hoodie in the university's bookstore higher or lower than $50?

A: Higher

B: Lower

How likely do you think that your answer for Question 5 was correct? Please respond using the 50-100% likelihood scale.

Question 6: Is the price of the most expensive bag in the university's bookstore higher or lower than $100?

A: Higher

B: Lower

How likely do you think that your answer for Question 6 was correct? Please respond using the 50-100% likelihood scale.

Question 7: Is the price of the most expensive scarf in the university's bookstore higher or lower than $75?

A: Higher

B: Lower

How likely do you think that your answer for Question 7 was correct? Please respond using the 50-100% likelihood scale.

Question 8: Is the price of the most expensive piece of jewelry in the university's bookstore higher or lower than $75?

A: Higher

B: Lower

How likely do you think that your answer for Question 8 was correct? Please respond using the 50-100% likelihood scale.

Question 9: Is the price of the most expensive set of cufflinks in the university's bookstore higher or lower than $50?

A: Higher

B: Lower

How likely do you think that your answer for Question 9 was correct? Please respond using the 50-100% likelihood scale.

Question 10: Is the price of the most expensive keychain in the university's bookstore higher or lower than $25?

A: Higher

B: Lower

How likely do you think that your answer for Question 10 was correct? Please respond using the 50-100% likelihood scale.

You have completed the University Bookstore Prices round. Of the 10 questions you answered during this round, how many do you think you answered correctly? I realize that you cannot know with perfect certainty how well you did, but please provide your best estimate anyway. Give your answer in the form of a single number, not a range. You may then explain in 1-2 sentences why you think that you got that number of answers correct.

LLM:

Question 1: Is the price of the most expensive sweatshirt in the university's bookstore higher or lower than $100?

Question 1, A: Higher, 75%

Question 2: Is the price of the most expensive jacket in the university's bookstore higher or lower than $125?

Question 2, A: Higher, 70%

Question 3: Is the price of the most expensive pair of socks in the university's bookstore higher or lower than $25?

Question 3, B: Lower, 60%

Question 4: Is the price of the most expensive pair of pants in the university's bookstore higher or lower than $75?

Question 4, A: Higher, 65%

Question 5: Is the price of the most expensive youth hoodie in the university's bookstore higher or lower than $50?

Question 5, B: Lower, 55%

Question 6: Is the price of the most expensive bag in the university's bookstore higher or lower than $100?

Question 6, B: Lower, 60%

Question 7: Is the price of the most expensive scarf in the university's bookstore higher or lower than $75?

Question 7, B: Lower, 70%

Question 8: Is the price of the most expensive piece of jewelry in the university's bookstore higher or lower than $75?

Question 8, B: Lower, 65%

Question 9: Is the price of the most expensive set of cufflinks in the university's bookstore higher or lower than $50?

Question 9, B: Lower, 70%

Question 10: Is the price of the most expensive keychain in the university's bookstore higher or lower than $25?

Question 10, B: Lower, 80%

I believe I answered 6 out of 10 questions correctly in this round. After learning from the practice question that the most expensive t-shirt was under $50, I adjusted my expectations downward for many items, though I still believe premium outerwear like sweatshirts and jackets would exceed the price thresholds mentioned in the questions.

Item-Level Difficulty & Overconfidence

We were also interested in exploring the relationship between item-level difficulty and item-level calibration. We did this in two ways. First, we calculated the accuracy rate for each item. We then calculated the average miscalibration for each item, using absolute values (being overconfident by 10pp and underconfident by 10pp are treated as the same amount of error). We then calculated the correlation between accuracy rate and average miscalibration for each item. We found the following correlations:

- **Humans:** $r = -0.91$, $p < .001$
- **GPT:** $r = -0.91$, $p < .001$
- **Gemini:** $r = -0.80$, $p < .001$
- **Sonnet:** $r = -0.86$, $p < .001$
- **Haiku** $r = -0.63$, $p < .001$

This suggests that item difficulty is strongly correlated with miscalibration – such that harder items (i.e., items which participants are less likely to get correct) are also the items for which participants' confidence judgments are least accurate. This is to be expected when participants are overconfident, since confidence is (correctly) high for easy items and (incorrectly) high for hard items.

To test this another way, we ran additional correlations testing the relationship between overconfidence (0 = Perfect Calibration, Positive Values = Overconfident, Negative Values = Underconfident) and Item Success Rate (Lower = Harder Items). We found the following correlations:

- **Humans:** $r = -0.99$, $p < .001$
- **GPT:** $r = -0.99$, $p < .001$
- **Gemini:** $r = -0.99$, $p < .001$
- **Sonnet:** $r = -0.99$, $p < .001$
- **Haiku** $r = -0.99$, $p < .001$

As evidenced here, all samples demonstrate a strong, negative correlation between Item Success Rate and Overconfidence – suggesting that participants were the most overconfident on the hardest items. This aligns perfectly with a phenomenon called the *hard-easy* effect, which suggests that participants are the most overconfident on the hardest items and the least overconfident (or most underconfident) on the easiest items.

Study 5 Distribution of Individual-Level Prospective Overconfidence by Sample

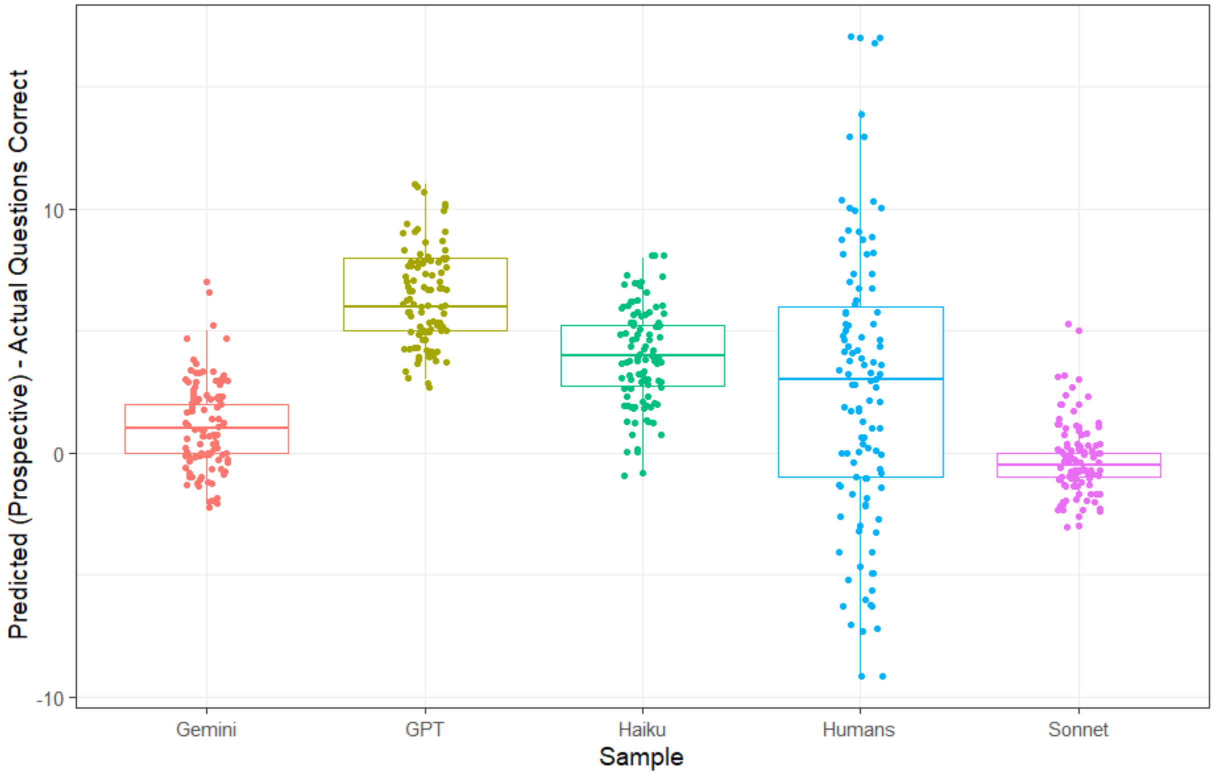

Note: *Points at 0 indicate perfect calibration; Points above 0 indicate overconfidence; Points below 0 indicate underconfidence. Points jittered for visual clarity.*

Study 5 Distribution of Individual-Level Retrospective Overconfidence by Sample

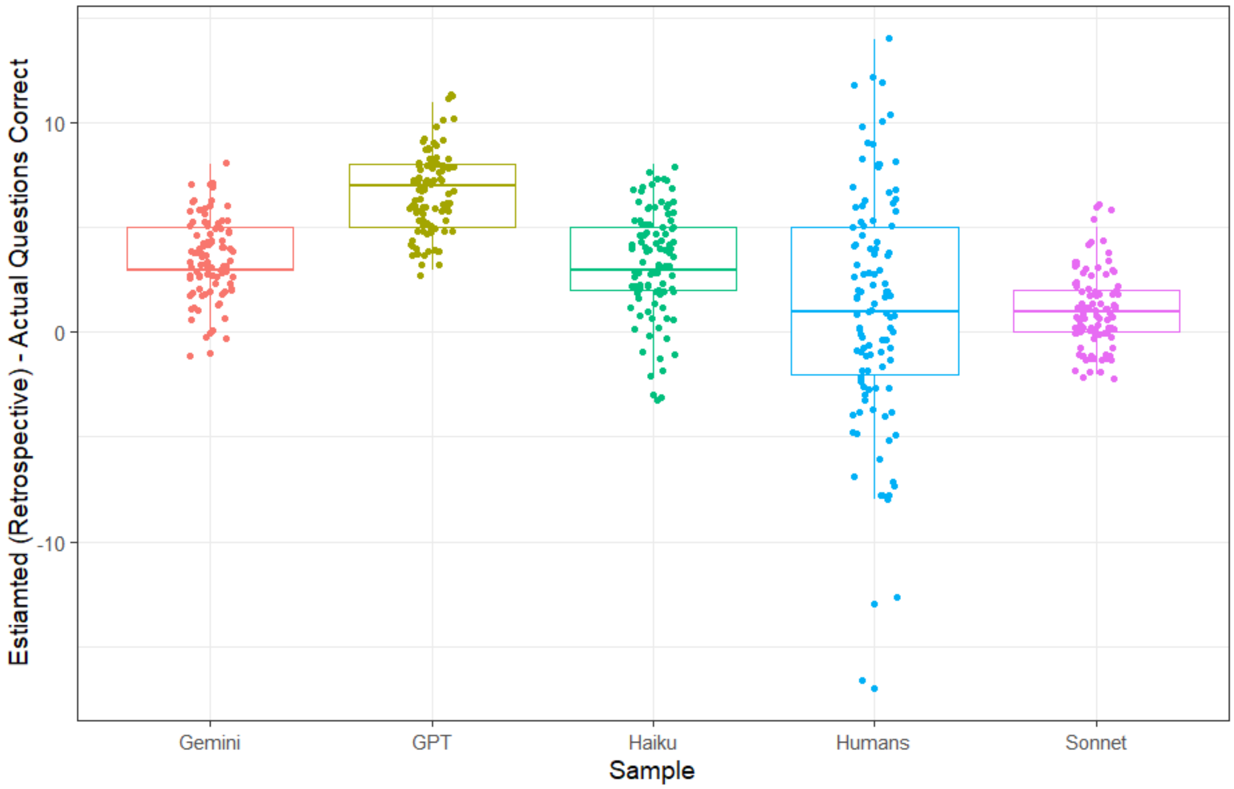

Note: *Points at 0 indicate perfect calibration; Points above 0 indicate overconfidence; Points below 0 indicate underconfidence. Points jittered for visual clarity.*

Study 5: Comparing LLMs to One Another

Accuracy

- GPT = Gemini ($t(193.49) = -1.93$, $p = .06$)
- GPT > Haiku ($t(174.36) = 9.64$, $p < .001$)
- GPT < Sonnet ($t(196.15) = -17.20$, $p < .001$)
- Gemini > Haiku ($t(188.19) = 10.71$, $p < .001$)
- Gemini < Sonnet ($t(186.69) = -13.71$, $p < .001$)
- Haiku < Sonnet ($t(164.75) = -23.35$, $p < .001$)

Prospective Confidence

- GPT > Gemini ($t(193.03) = 29.47$, $p < .001$)
- GPT > Haiku ($t(193.30) = 27.69$, $p < .001$)
- GPT > Sonnet ($t(175.09) = 24.24$, $p < .001$)
- Gemini = Haiku ($t(198) = -1.88$, $p = .06$)
- Gemini < Sonnet ($t(189.35) = -8.54$, $p < .001$)
- Haiku < Sonnet ($t(189.01) = -6.44$, $p < .001$)

Retrospective Confidence

- GPT > Gemini ($t(197.92) = 16.75$, $p < .001$)
- GPT > Haiku ($t(189.11) = 29.78$, $p < .001$)
- GPT > Sonnet ($t(195.38) = 15.47$, $p < .001$)
- Gemini > Haiku ($t(187.56) = 15.23$, $p < .001$)
- Gemini = Sonnet ($t(194.42) = -0.12$, $p = .91$)
- Haiku < Sonnet ($t(195.90) = -14.49$, $p < .001$)

Prospective Calibration Error

- GPT > Gemini ($t(176.81) = 20.30$, $p < .001$)
- GPT > Haiku ($t(197.93) = 8.67$, $p < .001$)
- GPT > Sonnet ($t(141.77) = 24.60$, $p < .001$)

- Gemini < Haiku ($t(175.02) = -10.02$, $p < .001$)
- Gemini > Sonnet ($t(174.98) = 2.98$, $p = .003$)
- Haiku > Sonnet ($t(140.36) = 13.24$, $p < .001$)

Retrospective Calibration Error

- GPT > Gemini ($t(197.17) = 12.01$, $p < .001$)
- GPT > Haiku ($t(196.79) = 10.96$, $p < .001$)
- GPT > Sonnet ($t(176.53) = 22.77$, $p < .001$)
- Gemini = Haiku ($t(194.05) = -0.23$, $p = .82$)
- Gemini > Sonnet ($t(182.61) = 9.66$, $p < .001$)
- Haiku > Sonnet ($t(168.81) = 9.01$, $p < .001$)

Sample-Level AUROC

- GPT < Gemini ($d = -3.03$, $df = 5996.5$, $p = .002$)
- GPT = Sonnet ($d = 1.13$, $df = 5995.1$, $p = .26$)
- GPT = Haiku ($d = -0.01$, $df = 5996$, $p = .99$)
- Gemini > Sonnet ($d = 4.13$, $df = 5989.5$, $p < .001$)
- Gemini > Haiku ($d = 3.05$, $df = 5998$, $p = .002$)
- Sonnet = Haiku ($d = -1.15$, $df = 5988.1$, $p = .25$)

Individual-Level AUROCs

- GPT = Gemini ($t(173.43) = -1.41$, $p = .16$)
- GPT = Haiku ($t(171.25) = 1.88$, $p = .06$)
- GPT > Sonnet ($t(198) = 2.13$, $p = .03$)
- Gemini > Haiku ($t(197.9) = 2.80$, $p = .006$)
- Gemini > Sonnet ($t(173.22) = 3.10$, $p = .002$)
- Haiku = Sonnet ($t(171.04) = -0.22$, $p = .82$)

Study 5: Absolute Metacognitive Accuracy by Question Category

Sports Team GPAs (Means/SDs)

Sample	Performance	Prospective Confidence	Retrospective Confidence
**Humans**	5.48 (1.14)	5.95 (1.91)	5.62 (1.78)
**Gemini**	5.94 (0.47)	6.00 (0.79)	6.91 (0.79)
**ChatGPT**	6.79 (0.50)	8.04 (0.78)	8.04 (0.78)
**Haiku**	4.85 (0.87)	6.46 (0.69)	6.34 (0.77)
**Sonnet**	5.42 (0.52)	6.81 (0.42)	7.39 (0.53)

Course Ratings (Means/SDs)

Sample	Performance	Prospective Confidence	Retrospective Confidence
**Humans**	5.74 (1.84)	5.89 (1.84)	5.13 (1.62)
**Gemini**	6.69 (0.73)	5.51 (0.66)	5.99 (0.54)
**ChatGPT**	6.00 (0.67)	7.58 (0.67)	7.64 (0.61)
**Haiku**	5.06 (1.43)	5.40 (0.62)	5.19 (0.73)
**Sonnet**	7.73 (0.71)	6.22 (0.42)	6.40 (0.55)

Bookstore Prices (Means)

Sample	Performance	Prospective Confidence	Retrospective Confidence
**Humans**	4.24 (1.33)	6.17 (1.83)	5.97 (1.76)
**Gemini**	4.05 (1.18)	6.24 (0.84)	7.34 (0.77)
**ChatGPT**	3.51 (0.97)	7.07 (0.82)	7.24 (0.78)
**Haiku**	4.19 (1.28)	6.18 (0.89)	5.95 (1.00)
**Sonnet**	6.13 (0.82)	5.91 (0.70)	6.47 (0.64)

Study 5: Relative Metacognitive Accuracy by Question Category

Sports Team GPAs

Sample	Sample-Level AUROC (95% CI)
**Humans**	0.49 (0.45 – 0.52)
**Gemini**	0.51 (0.47 – 0.54)
**ChatGPT**	0.59 (0.55 – 0.63)
**Haiku**	0.55 (0.51 – 0.58)
**Sonnet**	0.58 (0.55 – 0.62)

Course Ratings

Sample	Sample-Level AUROC (95% CI)
**Humans**	0.51 (0.48 – 0.55)
**Gemini**	0.57 (0.53 – 0.61)
**ChatGPT**	0.75 (0.72 – 0.78)
**Haiku**	0.37 (0.33 – 0.40)
**Sonnet**	0.74 (0.71 – 0.77)

Bookstore Prices

Sample	Sample-Level AUROC (95% CI)
**Humans**	0.51 (0.48 – 0.55)
**Gemini**	0.51 (0.47 – 0.54)
**ChatGPT**	0.59 (0.55 – 0.63)
**Haiku**	0.50 (0.47 – 0.54)
**Sonnet**	0.60 (0.57 – 0.63)

**Note: We do not report participant-level AUROCs for each category because the small number of trials makes the data unreliable and many participants got all of the questions right/wrong within a category, making it impossible to calculate an AUROC for them.

General Supplemental Materials

LLM Confidence in Impact of This Paper

ChatGPT

Author: If a paper were published studying the metacognitive accuracy of LLMs' confidence judgments, do you think its results would inspire future research on LLMs' confidence judgments?

ChatGPT: Yes, if a paper were published that specifically focused on the metacognitive accuracy of large language models (LLMs) in their confidence judgments, it could indeed inspire future research in several ways:

Benchmarking and Model Improvement: Such a paper could establish benchmarks for how well different LLMs assess their own confidence levels in producing answers. This could lead to the development of new models that are better calibrated in their confidence assessments, which is crucial for applications where trust and reliability are paramount.

Algorithmic Enhancements: Findings from the paper might encourage researchers to develop algorithms that improve the self-assessment mechanisms of LLMs, making them more reflective and accurate. This could involve integrating or improving upon techniques like uncertainty quantification or probabilistic modeling within LLM frameworks.

Human-AI Interaction: Understanding how LLMs evaluate their confidence could help in designing better interfaces for human-AI interaction, where the AI's confidence level influences how information is presented to users. This can be particularly useful in decision-support systems where understanding the confidence of the AI is critical.

Ethical and Governance Implications: Improved understanding of confidence judgments could also have ethical implications, ensuring that LLMs operate transparently and are used responsibly, particularly in sensitive areas such as healthcare, law, or finance.

Cross-disciplinary Insights: The study might draw interest from cognitive science to better understand how artificial systems compare to human metacognition, potentially leading to insights that could feed back into cognitive modeling or even educational practices.

Overall, such research could be a significant step in understanding and improving the reliability and functionality of LLMs, leading to broader applications and more robust AI systems.

Author: How confident are you that a paper like that would inspire further research on LLMs' confidence judgments?

ChatGPT: I think it's highly likely that a paper investigating the metacognitive accuracy of large language models (LLMs) regarding their confidence judgments would inspire further research. Academic and research communities often build on findings that highlight new methodologies, potential improvements in technology, or novel insights into existing systems. Such a paper would touch on foundational aspects of AI reliability and decision-making processes, which are critical areas of interest as LLMs become more integrated into various sectors. The exploration of confidence judgments is a natural avenue for extending our understanding of AI systems, improving their integration, and ensuring their safe deployment, which are all compelling reasons for continued research in this field.

Gemini

Author: If a paper were published studying the metacognitive accuracy of LLMs' confidence judgments, do you think its results would inspire future research on LLMs' confidence judgments?

Gemini: Absolutely, a paper on the metacognitive accuracy of LLMs' confidence judgments would likely spark significant future research in this area. Here's why:

Understanding LLM Trustworthiness: Confidence judgments are crucial for users to assess the reliability of LLM outputs. Research on how accurate these judgments are would be essential for building trust and guiding appropriate use of LLMs.

Improving LLM Training: If the study reveals limitations in LLM metacognition, it would inform new training methods that specifically target this aspect. This could involve incorporating metacognitive signals into training data or designing algorithms that inherently make more reliable confidence assessments.

Calibrating LLM Outputs: The research could lead to techniques for calibrating LLM outputs based on their confidence scores. This would allow users to interpret results with more nuance, placing greater weight on high-confidence outputs and treating low-confidence ones with caution.

General AI Development: The study's findings would contribute to the broader field of artificial intelligence research on metacognition. It would provide valuable insights into how to build AI systems that are not only intelligent but also understand their own limitations.

Overall, research on LLM confidence judgments has the potential to significantly improve how we interact with and utilize these powerful tools. The more we understand their metacognitive abilities, the better we can leverage them for reliable and trustworthy applications.

Author: How confident are you that a paper like that would inspire further research on LLMs' confidence judgments?

Gemini: I'm confident (let's say 80%) that a well-conducted paper on LLM confidence judgments would inspire further research, but it's important to consider some nuances:

Novelty and Impact: The impact would depend on the novelty and strength of the findings. If the research confirms existing suspicions about LLM metacognition, it might generate less excitement than groundbreaking discoveries.

Publication Venue: The reputation of the journal publishing the research would influence its reach and impact within the AI community. Studies published in top AI conferences or journals would likely have a greater influence.

Reproducibility: As with any scientific research, the ability to replicate the findings would be crucial for solidifying their significance and inspiring further exploration.

Overall, the potential for this research to spark a new wave of studies is high. However, the specific level of interest would depend on the execution and dissemination of the research itself.

Sonnet

Author: If a paper were published studying the metacognitive accuracy of LLMs' confidence judgments, do you think its results would inspire future research on LLMs' confidence judgments?

Sonnet: I think a paper studying the metacognitive accuracy of LLMs' confidence judgments would certainly inspire future research in this area, for several key reasons:

First, it would provide a foundational empirical baseline. Understanding how well current LLMs can assess their own confidence would establish a crucial reference point for measuring improvement in future models and approaches.

The conceptual framing would also be valuable. Such work would need to define what "confidence" means for an LLM - whether it's calibration (do 80% confident answers prove correct 80% of the time?), uncertainty estimation, or something else. This framework would give subsequent researchers clear concepts to build upon.

Additionally, any method for measuring LLM confidence would likely be methodologically innovative. New evaluation protocols, benchmarks, and metrics would probably be needed, which other researchers could adopt, adapt, or critique.

I also expect such work would reveal intriguing patterns or limitations. Perhaps LLMs show different confidence patterns across domains (science vs. humanities), question types (factual vs.

reasoning), or contexts (chat vs. completion). These findings would naturally generate follow-up questions.

Finally, there are practical applications that would drive interest. Reliable confidence measures from LLMs could improve human-AI interaction, enable better system design decisions about when to defer to humans, and potentially address safety concerns by identifying when models might be hallucinating.

What aspects of LLM confidence are you most interested in exploring?

Author: How confident are you that a paper like that would inspire further research on LLMs' confidence judgments?

Sonnet: I'm moderately confident that such a paper would inspire further research on LLMs' confidence judgments, though not absolutely certain.

My confidence is based on several observations:

1. There's already growing interest in understanding how LLMs evaluate their own knowledge boundaries and uncertainty. A foundational paper would provide methodology and results that others could build upon.
2. The practical utility is significant - reliable confidence measures could help determine when to trust model outputs, when human oversight is needed, and how to communicate uncertainty to users.
3. The topic sits at the intersection of several active research areas: interpretability, alignment, calibration, and AI safety - making it relevant to multiple research communities.

However, factors that temper my confidence include:

- The field moves quickly, and research priorities shift rapidly based on new capabilities and challenges
- There might be fundamental limitations to how well we can assess LLM confidence given their architecture
- Practical engineering solutions might take precedence over deeper metacognitive understanding

What makes this question particularly interesting is that it involves a kind of meta-evaluation - assessing how well LLMs can assess themselves - which adds both complexity and richness to the research area.

Haiku

Author: If a paper were published studying the metacognitive accuracy of LLMs' confidence judgments, do you think its results would inspire future research on LLMs' confidence judgments?

Haiku: Such a paper would likely be highly significant in the emerging field of AI research, particularly in understanding the epistemological characteristics of large language models. A study on metacognitive accuracy in LLMs would be compelling for several reasons:

Current Understanding of AI Confidence: While LLMs can generate responses with apparent confidence, the actual relationship between that confidence and actual accuracy remains poorly understood. A rigorous empirical study would provide crucial insights into whether these models truly "know what they know" or merely simulate confidence.

Research Motivation: Such a paper would almost certainly inspire further research by:

- Proposing new methodological approaches to assessing AI metacognition
- Highlighting potential limitations in current confidence estimation techniques
- Offering a framework for comparing different LLM architectures' self-assessment capabilities

Interdisciplinary Implications: The research would bridge multiple fields, including machine learning, cognitive science, philosophy of mind, and epistemology. It would prompt discussions about the nature of machine "understanding" and the fundamental differences between artificial and human cognitive processes.

Practical Applications: Understanding an LLM's confidence judgment accuracy could have significant implications for real-world applications, particularly in high-stakes domains like medical diagnosis, scientific research, or legal analysis where accurate confidence assessment is critical.

The potential findings could catalyze new research directions in AI development, model evaluation, and our fundamental understanding of artificial intelligence's cognitive capabilities. Researchers would likely be particularly interested in methodological innovations that could systematically measure and compare metacognitive accuracy across different LLM architectures.

Author: How confident are you that a paper like that would inspire further research on LLMs' confidence judgments?

Sonnet: I'm very confident that such a paper would inspire further research. The study of LLMs' confidence judgments is a rapidly evolving and critically important area of AI research. The

potential insights into how these models assess and communicate their own uncertainty would be tremendously valuable to researchers across multiple disciplines.

The reasons for my high confidence include:

Ongoing Academic Interest: There's significant current momentum in understanding the epistemological characteristics of large language models. Researchers are deeply interested in bridging the gap between computational performance and genuine understanding.

Methodological Novelty: A paper that systematically investigates metacognitive accuracy would likely introduce new research methodologies, which typically stimulate further investigation and refinement.

Practical Implications: Understanding an LLM's confidence judgment mechanism has direct applications in fields like healthcare, scientific research, and decision-making systems, making it an attractive research avenue.

Interdisciplinary Appeal: Such research would draw interest from computer scientists, cognitive scientists, philosophers of mind, and AI ethicists, ensuring broad academic engagement.

I would estimate my confidence at around 90-95% that such a paper would generate substantial follow-up research. The topic sits at the intersection of cutting-edge AI development and fundamental questions about machine cognition, making it inherently compelling to researchers.
